# An Interactive, Case-Based Workshop on the Patient Experience for Internal Medicine Residents

**DOI:** 10.15766/mep_2374-8265.11442

**Published:** 2024-10-01

**Authors:** Julian Swanson, Doris Lin, Kristen A. Staggers, Priti Dangayach

**Affiliations:** 1 Assistant Professor, Department of Medicine, Baylor College of Medicine; 2 Associate Professor, Department of Medicine, Baylor College of Medicine; 3 Biostatistician, Institute for Clinical and Translational Research, Baylor College of Medicine

**Keywords:** Bedside Manner, HCAHPS, Patient Experience, Patient Interview, Patient Satisfaction, Quality of Care, Case-Based Learning, Communication Skills, Internal Medicine, Professionalism, Quality Improvement/Patient Safety

## Abstract

**Introduction:**

The technological revolution has narrowed the information gap between physician and patient. This has led to an evolution in medicine from paternalistic to patient-centric, with health care systems now prioritizing patient experience to achieve higher satisfaction scores. Therefore, it is imperative to start early in educating trainees on how to best address the holistic needs of the patient while also delivering high-quality care.

**Methods:**

We implemented a 1-hour workshop that was repeated weekly over 8 weeks to capture all internal medicine residents in our program. During the workshop, we reviewed the historical evolution of patient care from paternalistic to patient-centered, presented the Hospital Consumer Assessment of Healthcare Providers and Systems survey questions, and discussed evidence-based strategies for physicians to improve their patients’ experience utilizing four case-based scenarios.

**Results:**

Over the 8-week period, a total of 195 residents participated in the workshop. One hundred thirty-nine residents (71%) completed the pre- and postsession survey. Results demonstrated significant knowledge improvement (*p* < .001) in all of the topics discussed. Additionally, the majority of residents felt the workshop would be useful in their clinical practice and found the clinical scenarios useful.

**Discussion:**

Given the evolution towards patient-centered care, it is important to take a proactive approach in providing residents with the tools to best address their patients’ needs. Early understanding of patient satisfaction surveys and the impacts they have on hospital metrics can help trainees in their careers as practicing physicians.

## Educational Objectives

By the end of this activity, learners will be able to:

1. Identify how health care systems measure and track the patient experience.
2. Summarize the questions asked on the national standardized patient experience survey in reference to the physician-patient interaction.
3. Explain how hospitals use patient experience survey data for comparative performance.
4. Apply evidence-based tips to improve patient experience and the physician-patient relationship.

## Introduction

Over the past few decades, medical care has evolved from a paternalistic to a patient-centric model due to widespread access to health information through social media and the internet.^1^ The patient-centered model has now been adopted as the standard of care and emphasizes patient preferences and values at the forefront.^1^ The Agency for Healthcare Research and Quality has established patient-centered care as one of the pillars of health care quality for the 21st century.^2^ This change has been important for respecting patient autonomy and emphasizing communication through shared decision-making. In 1985, the health care industry began utilizing surveys as the main source of measuring whether the care provided met acceptable standards.^3^ The Hospital Consumer Assessment of Healthcare Providers and Systems (HCAHPS), the first national standardized survey, was developed in 2002 and is still currently distributed to most patients who have been admitted over 24 hours in participating hospitals.^3,4^

With the change in the doctor-patient dynamic, it is imperative to train physicians to address the growing needs of patients as their input is essential to improving the quality of care they receive.^5^ Studies have demonstrated that improving patient experience can lead to improved comfort in managing medical conditions, quality of life, medication compliance, follow-up, and abstinence from drugs, as well as a reduction in disease progression and pain.^6,7^ Higher patient satisfaction scores also correlate to lower physician burnout and improved physician satisfaction.^8,9^ Additionally, data from these surveys significantly affect hospital metrics and have a direct financial impact on reimbursement.^10^

In academic settings, residents are often the frontline providers and have increased face-to-face interaction with patients, likely leading many patients to consider the resident as the primary physician. This can significantly impact the way patients fill out satisfaction surveys.^11^ A cross-sectional study has found that a large percentage of residents are not educated and/or not aware of how patient experience is measured.^12^ Therefore, it is imperative to start early in educating trainees on how their interaction can affect survey responses.

With Accreditation Council for Graduate Medical Education duty-hour restrictions, there is currently limited time in residency training to incorporate lengthy formal didactics. We sought to condense high-value patient experience material into a concise 1-hour workshop. *MedEdPORTAL* has published curricula on patient experience, with one focusing on communication skills^13^ and another on satisfaction related to the emergency department experience.^14^ Both are lengthy, multisession curricula. Our curriculum differs in that we include a historical perspective on patient experience to help residents better understand why this training is fundamental to a career that is patient centric. In addition, our workshop is unique in that it engages the audience using both PowerPoint didactics and interactive, real-life, case-based scenarios to achieve our educational objectives.

The main purpose of this resource is to address a current deficiency in residency education by teaching trainees a topic of prime importance to their careers as practicing physicians and to health care systems as a whole. Our aims are to improve resident knowledge of (1) how hospitals measure and track patient satisfaction, (2) the HCAHPS survey questions, (3) how hospitals use HCAHPS survey data to compare to one another, and (4) practical strategies to improve patient experience.

## Methods

Our internal medicine residency program had a Wednesday School dedicated to formal didactics. We implemented the workshop and repeated it weekly over an 8-week period from October to November of 2022 to capture all the residents in the program. Given significant time constraints in graduate medical education, we included what we thought were the most salient educational aspects of the patient experience in a single session. Two of the authors, who had prior knowledge of patient experience and satisfaction surveys, developed the PowerPoint presentation and case scenarios. Each participant completed a pre- and postsession online survey (Appendices A and B) through QR codes. These QR codes were displayed on the initial PowerPoint slide prior to the start of the workshop and the final PowerPoint slide upon completion of the workshop (Appendix C). The QR codes linked the participants to the electronic version of the survey. Two minutes were allotted to complete the presurvey and 5 minutes to complete the postsurvey. Of note, these QR codes are not included in this publication. The Baylor College of Medicine Institutional Review Board approved the survey.

After participants had finished the preworkshop survey (Appendix A), the presenters facilitated a discussion on what the trainees valued from their own experience with patients. Then, the presenters began the slide presentation (Appendix C), which reviewed the historical evolution of patient care from paternalistic to patient-centric and current ways hospitals measure patient experience, with specific focus on the HCAHPS survey questions that pertain to the doctor-patient interaction. (Future facilitators could easily review the PowerPoint slides, educational content, and structure prior to the presentation without necessarily having extensive prior knowledge or experience on this topic.) In seven of the eight workshops, an invited guest expert in patient experience from a partnering hospital helped answer questions regarding details of posthospitalization survey distribution and the data-collection process. Although an optional component of the workshop, it was helpful to have a guest expert available to answer questions using actual raw patient data and to discuss how hospital systems receive and use the data.

The second half of the workshop was an interactive brainstorming exercise. In preparation, we printed hard copies of the four difficult patient interaction scenarios (Appendix D), making enough copies for each pair of residents to receive one of the four scenarios. The topics of the scenarios focused on pain management, medical errors, adjustment, and communication. Each pair of residents received one scenario. Two minutes were given for each pair to share, review, and discuss the scenario. After the pair-share, we opened up the discussion to the entire group (about 3 minutes per case) as follows: We first asked a volunteer to read the scenario out loud for everyone, then pairs who had been given that scenario made suggestions on how to improve the patient experience in each case, followed by an open forum for others to chime in with their thoughts and input.

In the final part of the workshop, we concluded with evidence-based strategies all physicians could utilize to improve the doctor-patient interaction and, ultimately, the patient experience.

To keep within our time limit and to maintain the audience's attention, we set time goals for completion of each section. For the introduction and preworkshop survey, we aimed for 5 minutes. For the presentation of historical content, we aimed for 15 minutes, followed by the HCAHPS survey for approximately 15 minutes. Next came the interactive session with the pair-share, which we broke down into 5 minutes between pairs, followed by the whole-group discussion of each difficult scenario at 15 minutes. We also gave 5 minutes for the postsurvey (Appendix B).

We wanted to make the session interactive. Doing the workshop in a larger group setting (approximately 25 residents at a time), we were cautious about role-playing and putting residents on the spot in front of their peers. Instead, we opted for a pair-share brainstorming session on how to improve the patient's experience in case-based difficult patient situations (Appendix D), followed by an open forum for participants to share their thoughts with the larger group. This allowed each individual an opportunity to share out loud with the balance of perspective from a colleague, then the opportunity for the entire group to benefit from each pair-share discussion.

We assessed knowledge improvement of workshop topics using a 5-point Likert scale (1 = *strongly disagree,* 5 = *strongly agree*); pre- and postsession survey scores were compared using the Wilcoxon signed rank test. Survey responses were summarized by median with 25th and 75th percentiles or by frequency with percentage. We also included two test questions as a more objective assessment of knowledge improvement. Each test question offered five potential answer choices worth 1 point each, so the total that could be earned was 10 points.

## Results

Out of 195 internal medicine residents in attendance, 151 completed the preworkshop survey, and 180 completed the postworkshop survey. Some residents arrived after the workshop began and therefore completed only the postsurvey. At the end of all eight sessions, a total of 139 PGY 1-PGY 4 residents had completed both surveys. Of the 139 residents, 71% (*n* = 99) had never had prior training on this topic while 10% (*n* = 14) had had two or more prior sessions.

The primary aim of the workshop was to assess knowledge improvement on curriculum topics. Participants demonstrated significant knowledge improvement (*p* < .001) on the questions asked on the HCAHPS survey, how hospitals track patient experience, how hospitals use survey data to compare to one another, and methods participants could implement to improve patient experience. Additionally, resident understanding of how improving patient experience impacted health outcomes, belief that patient experience was an integral part of health care, and belief that this training was an important aspect for a practicing physician improved (*p* < .001; Table).

**Table.**
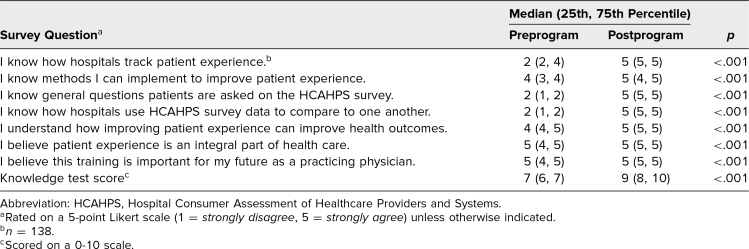
Responses to Self-Assessment Survey (*n* = 139)

We also incorporated two knowledge test questions on the postsession survey (Appendix B). The first test question asked the participant to “select the questions patients are asked on the postdischarge HCAHPS survey specifically related to their interaction with their physician,” and the second question asked the participant to “select the methods you, as the physician, can do to improve patient experience.” Participants demonstrated significant improvement in these knowledge test scores (*p* < .001) after completion of the workshop (Table).

Upon completion of the session, 96% of residents (*n* = 134) felt the workshop would be useful in their clinical practice, 87% (*n* = 120 out of 138) found the review of the HCAHPS survey useful, and 97% (*n* = 134 out of 138) found brainstorming through the clinical scenarios useful. Moreover, 96% (*n* = 133) found the presentation and slide content useful.

## Discussion

The preworkshop survey demonstrated that 71% of internal medicine residents at various levels of training had not had any formal didactics on patient experience. This reveals an important educational gap in the training of the next generation of physicians. We developed and implemented an innovative and time-conscious trainee workshop to overcome a current gap in graduate medical education and the limitation in adding didactics to an already compressed schedule. Results show that this workshop was effective in achieving its goals of not only educating residents regarding patient experience but also increasing the perceived importance and centrality of patient satisfaction in the medical field.

Our goal was to create a workshop to help residents understand the patient experience and how to improve it and meet their patients’ needs. Although our objectives were clear, it was difficult to then try to understand the entire system of patient experience and how hospitals use surveys as a reflection of patient perception and as motivation for change. It was extremely helpful to have a representative from our hospital's patient experience office on our team to help explain the nuances and inner workings of the hospital's response to the patient experience survey results.

We recognize a few limitations to the project design. First, our educational project lacked assessment of whether there was an actual change in real-life patient experience survey scores. Even though we provided an effective learning experience on how to improve the patient experience, it was beyond the scope of the project to assess real-life patient experience survey scores. Second, given the 1-hour time limitation allotted during the resident Wednesday School, we were unable to take a deeper dive into other material encompassing the patient experience. This includes topics such as racial and ethnic disparities, the continuum of health literacy, and structural/historical medical injustice leading to mistrust in the health care system. Third, our surveys primarily assessed self-reported knowledge improvement using a Likert scale, though we tried to remedy this by having two knowledge-check questions: one on identifying actual HCAHPS questions and the second on methods to improve the physician-patient interaction.

Other topics pertaining to the patient experience include improving the survey, the negative impact of unfavorable responses, and how the survey may be tied to physician compensation. As the objectives of this workshop are narrowly aimed at how to improve the patient experience, these additional topics could be developed into additional extensions of the current workshop.

We plan to use lessons learned from this educational project to improve future iterations. Although our results show improvement in knowledge regarding patient experience and specific patient experience survey questions, further studies are needed to assess whether this workshop influences patient experience scores beyond the classroom. For future iterations, we plan to include direct observation and individualized feedback for a smaller cohort of trainees based on their bedside interactions after completing the workshop and whether HCAHPS scores improve for this group, as well as identifying which strategies residents utilize more often to improve the interaction. Although we have included two test questions to objectively measure knowledge, we plan to incorporate more test questions in the future to better assess knowledge improvement, including questions regarding the outpatient Clinician & Group Consumer Assessment of Healthcare Providers and Systems survey as well.^15^

Our workshop provides historical background on the evolution to patient-centric care, presents HCAHPS survey questions with specific focus on the physician-patient interaction, and provides evidence-based strategies to improve patient experience and satisfaction scores. Medical residents spend a significant amount of time interacting with patients in academic hospitals but are not aware of the implications of their interaction on hospital metrics. Early understanding of these implications can make residents more likely to apply improvement strategies in their daily practice. Although we implemented this curriculum during dedicated educational time outside of clinical duties, the concise workshop can be easily incorporated into any residency program. It can also be used for faculty development to teach physicians at all levels the importance of this topic. This curriculum addresses a current deficiency in residency training and a topic of paramount importance to health care systems.

## Appendices


Preworkshop Survey.docxPostworkshop Survey.docxPatient Experience Workshop.pptxClinical Scenarios.docx
*All appendices are peer reviewed as integral parts of the Original Publication.*
