# Supplementary material for: An Interactive, Case-Based Workshop on the Patient Experience for Internal Medicine Residents
Source: MedEdPORTAL. 2024 Oct 1;20:11442. doi: 10.15766/mep_2374-8265.11442 (PMC11442592; doi:10.15766/mep_2374-8265.11442)
Supplement: Supplementary file 1 — Preworkshop Survey.docxPostworkshop Survey.docxPatient Experience Workshop.pptxClinical Scenarios.docx [file mep_2374-8265.11442-s001.zip › C. Patient Experience Workshop.pptx]

## Slide 1
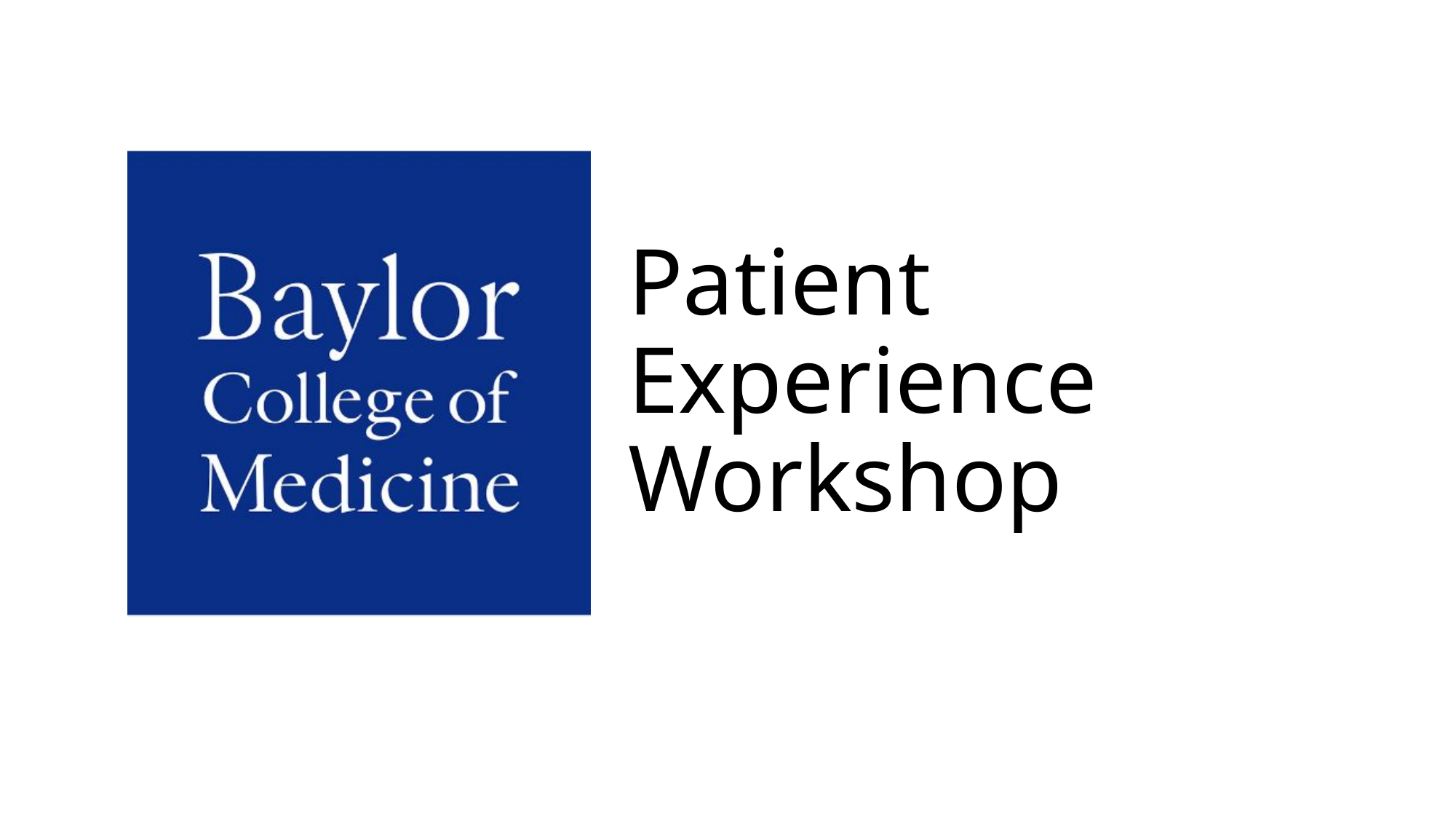

# Patient Experience Workshop

## Slide 2
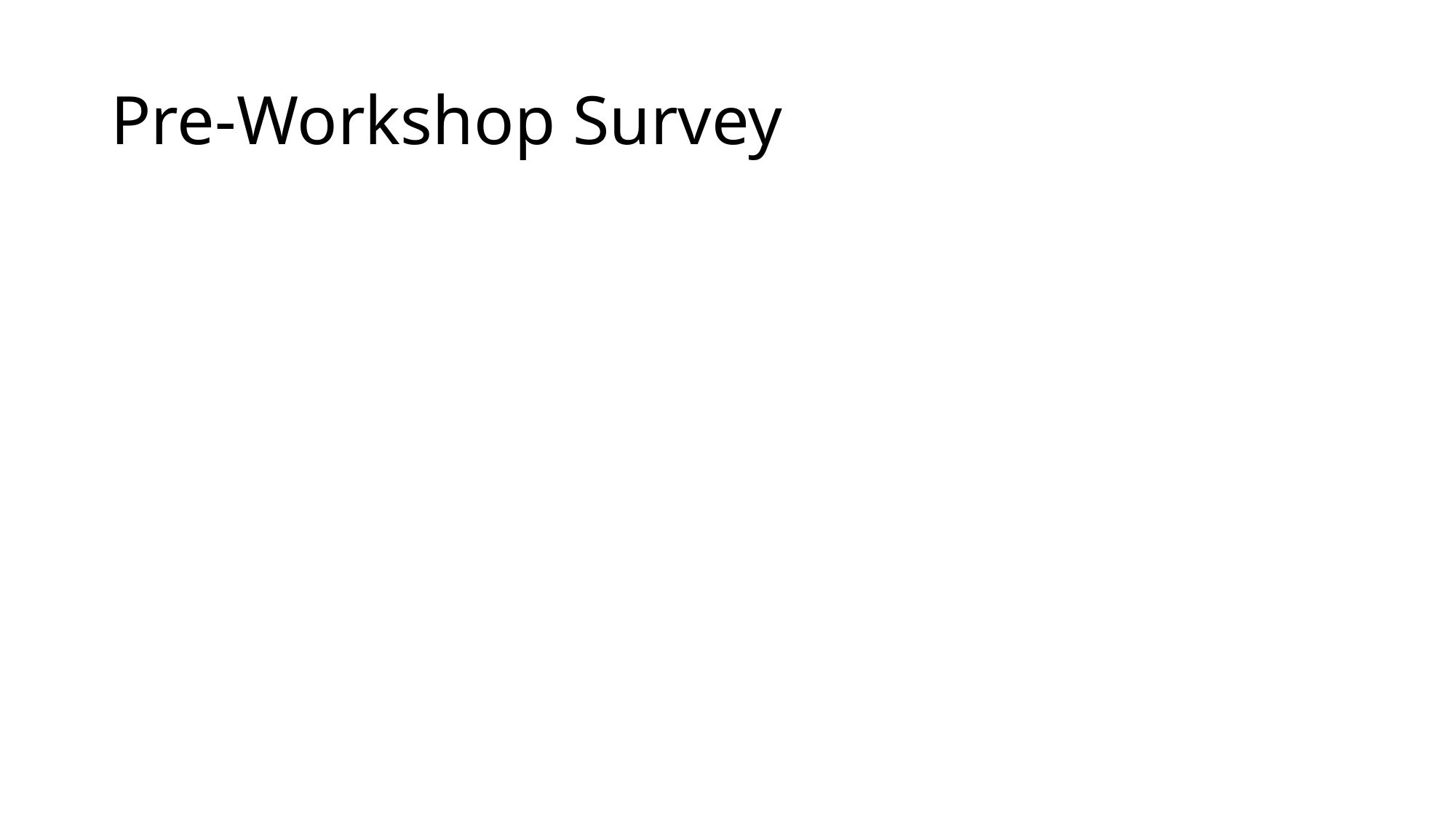

# Pre-Workshop Survey

## Slide 3
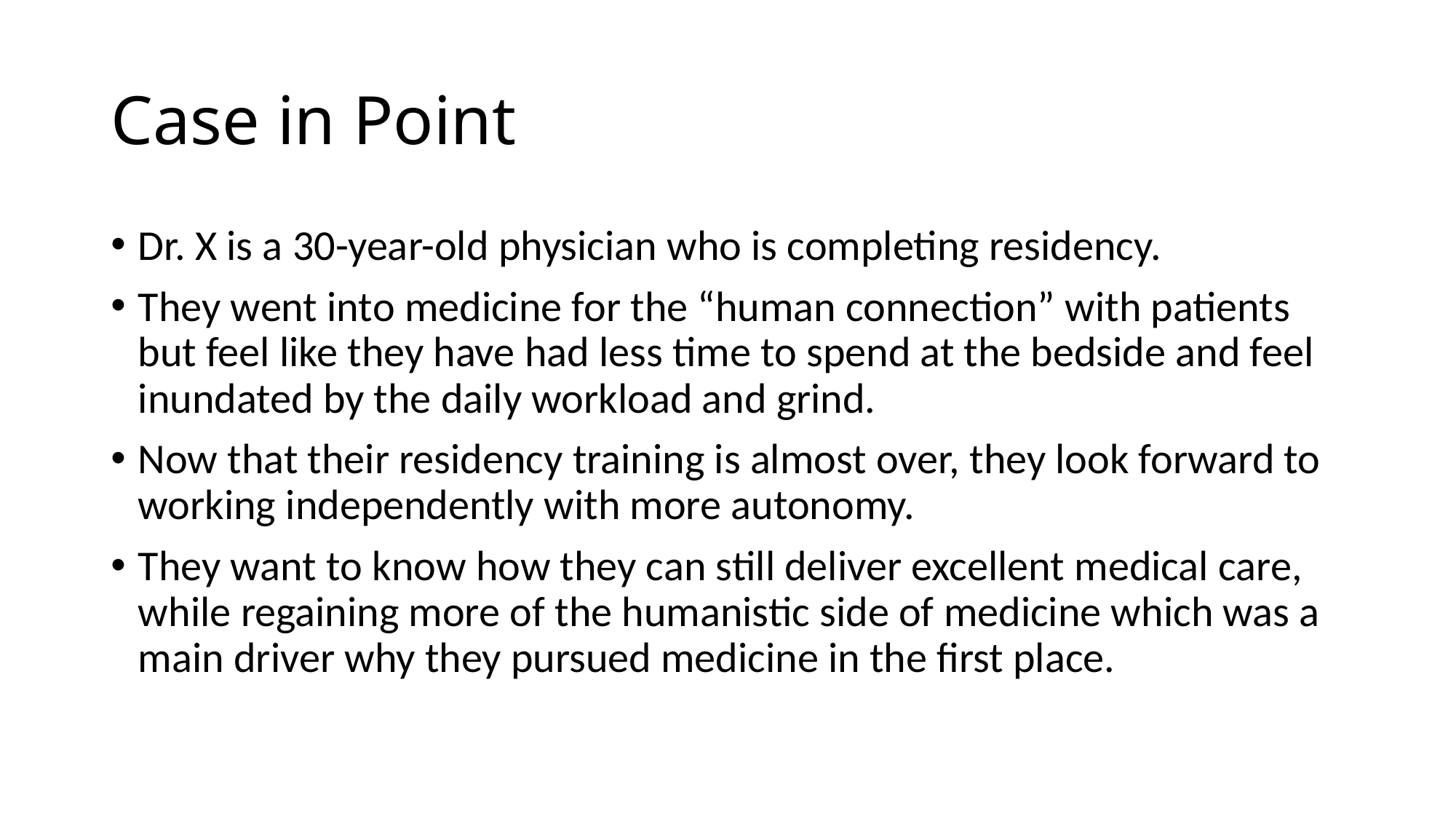

# Case in Point
Dr. X is a 30-year-old physician who is completing residency.
They went into medicine for the “human connection” with patients but feel like they have had less time to spend at the bedside and feel inundated by the daily workload and grind.
Now that their residency training is almost over, they look forward to working independently with more autonomy.
They want to know how they can still deliver excellent medical care, while regaining more of the humanistic side of medicine which was a main driver why they pursued medicine in the first place.

## Slide 4
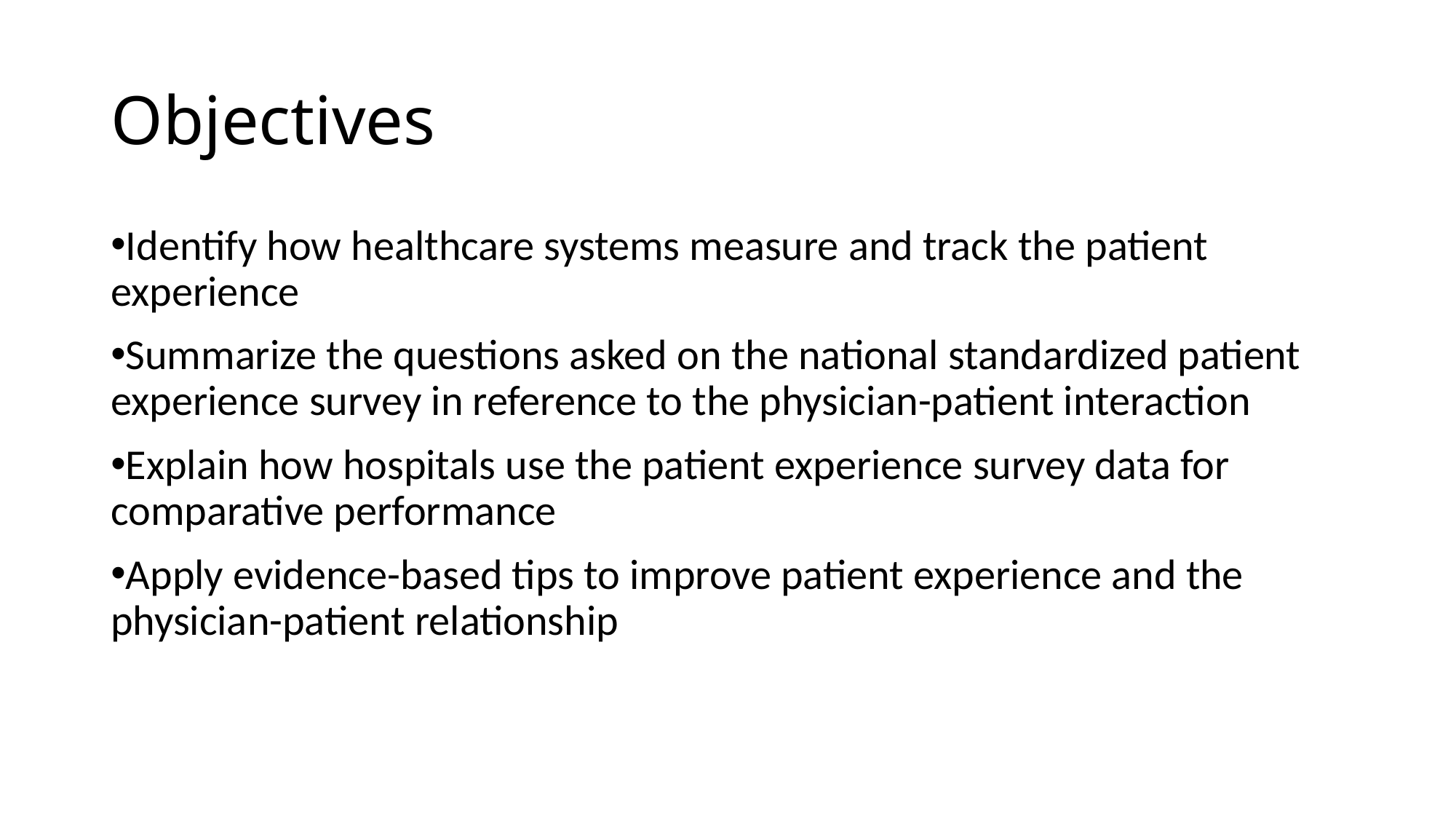

# Objectives
Identify how healthcare systems measure and track the patient experience
Summarize the questions asked on the national standardized patient experience survey in reference to the physician-patient interaction
Explain how hospitals use the patient experience survey data for comparative performance
Apply evidence-based tips to improve patient experience and the physician-patient relationship

## Slide 5
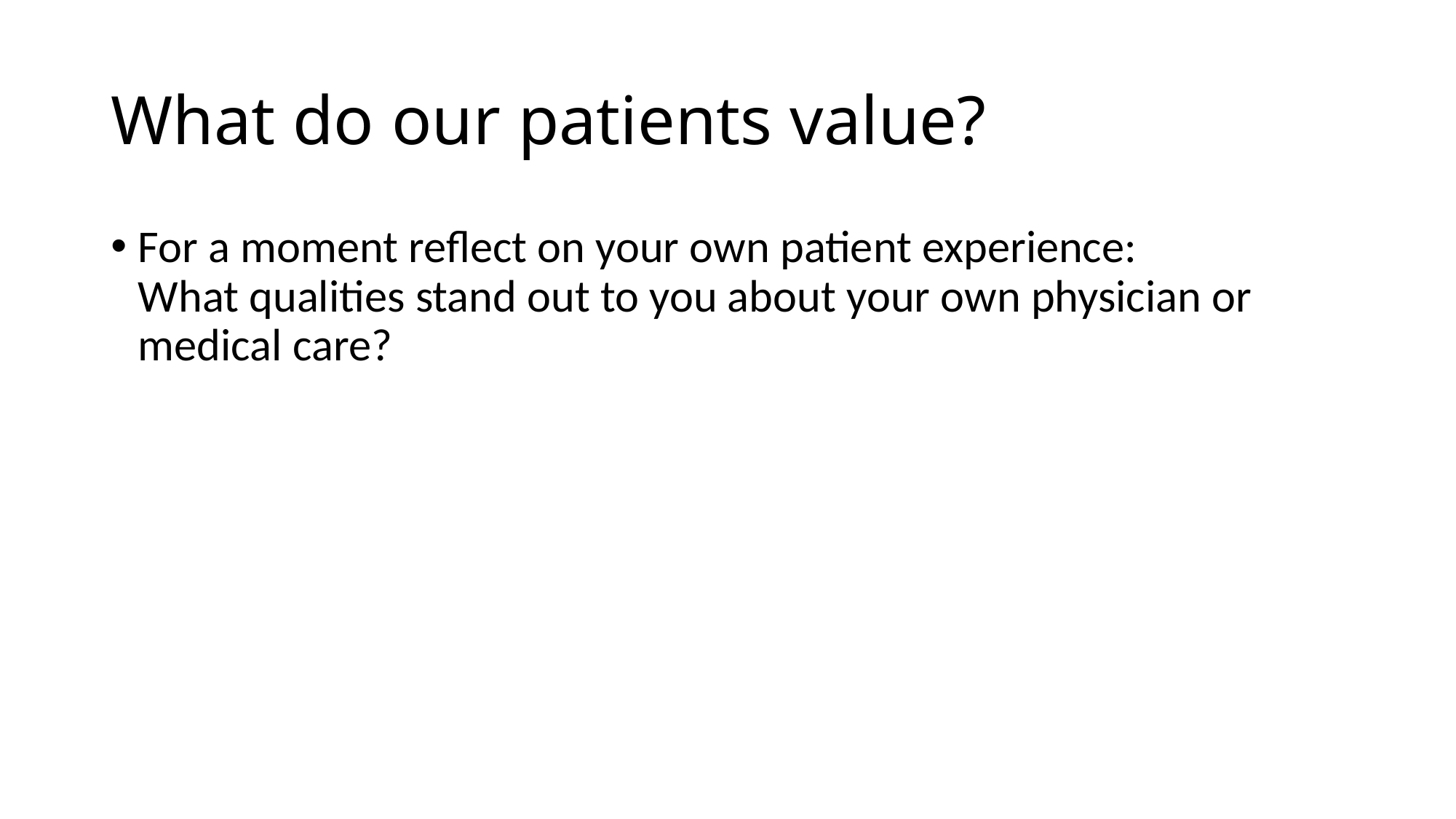

# What do our patients value?
For a moment reflect on your own patient experience: What qualities stand out to you about your own physician or medical care?

## Slide 6
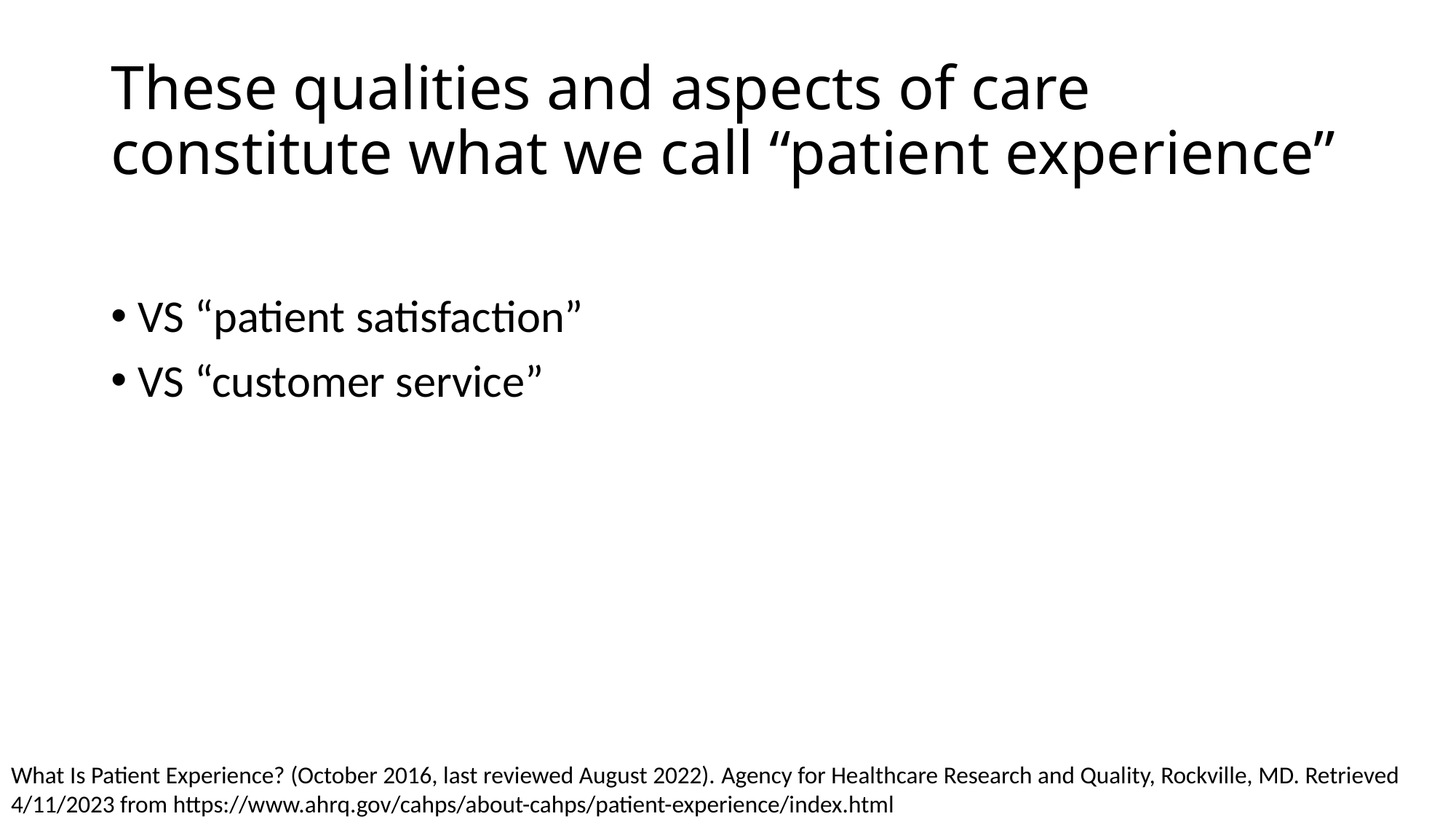

# These qualities and aspects of care constitute what we call “patient experience”
VS “patient satisfaction”
VS “customer service”
What Is Patient Experience? (October 2016, last reviewed August 2022). Agency for Healthcare Research and Quality, Rockville, MD. Retrieved 4/11/2023 from https://www.ahrq.gov/cahps/about-cahps/patient-experience/index.html

## Slide 7
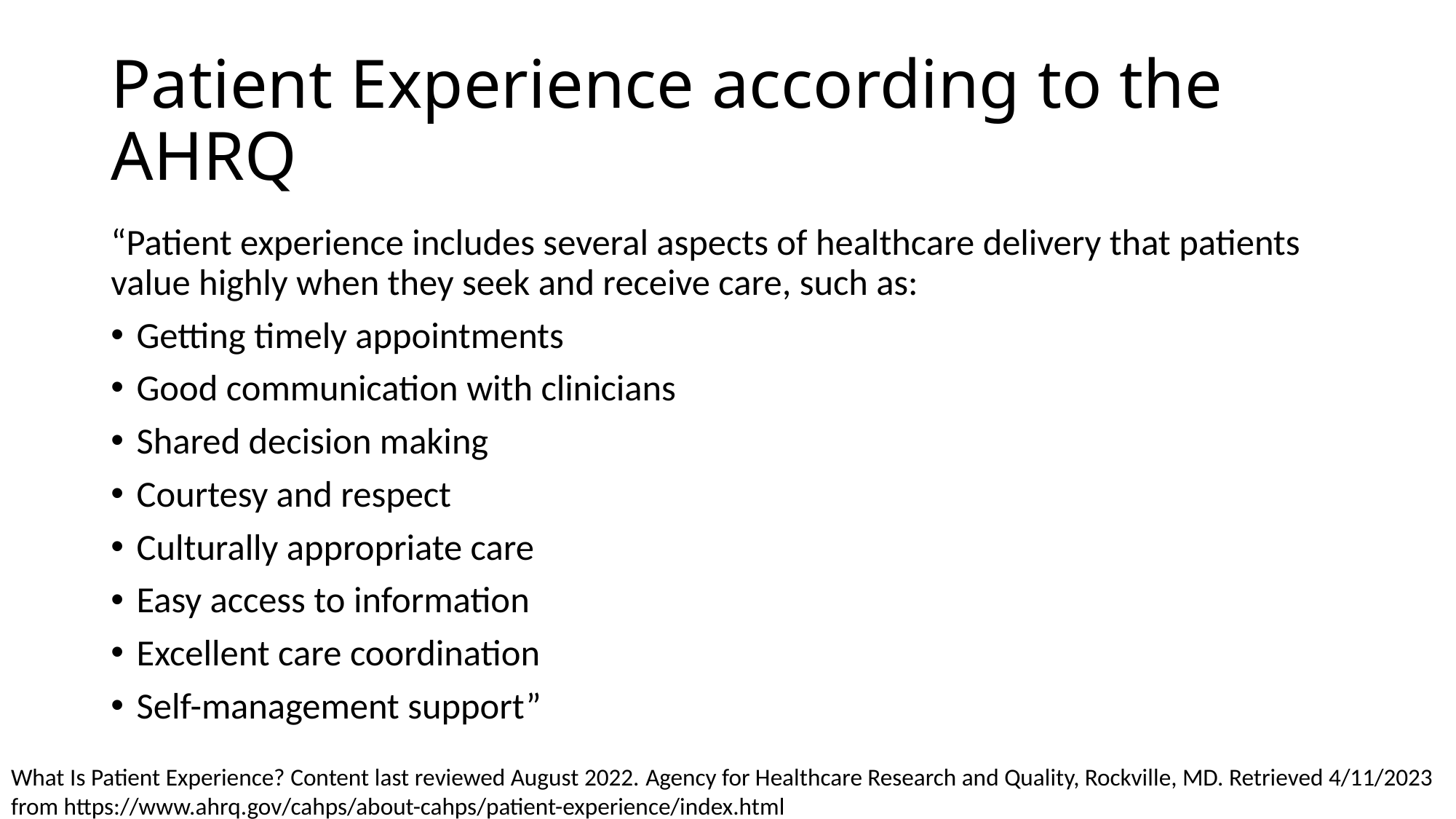

# Patient Experience according to the AHRQ
“Patient experience includes several aspects of healthcare delivery that patients value highly when they seek and receive care, such as:
Getting timely appointments
Good communication with clinicians
Shared decision making
Courtesy and respect
Culturally appropriate care
Easy access to information
Excellent care coordination
Self-management support”
What Is Patient Experience? Content last reviewed August 2022. Agency for Healthcare Research and Quality, Rockville, MD. Retrieved 4/11/2023 from https://www.ahrq.gov/cahps/about-cahps/patient-experience/index.html

## Slide 8
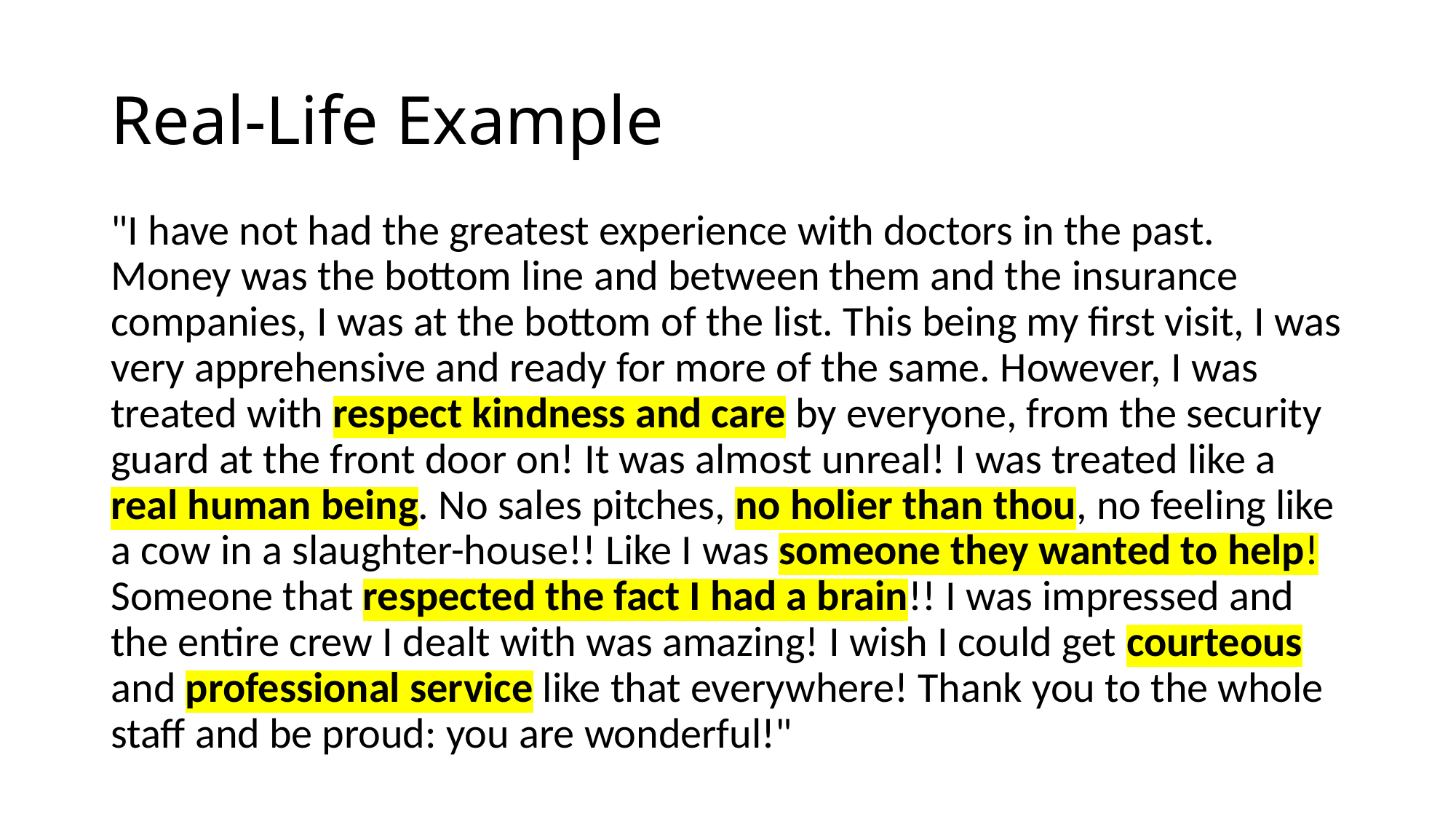

# Real-Life Example
"I have not had the greatest experience with doctors in the past. Money was the bottom line and between them and the insurance companies, I was at the bottom of the list. This being my first visit, I was very apprehensive and ready for more of the same. However, I was treated with respect kindness and care by everyone, from the security guard at the front door on! It was almost unreal! I was treated like a real human being. No sales pitches, no holier than thou, no feeling like a cow in a slaughter-house!! Like I was someone they wanted to help! Someone that respected the fact I had a brain!! I was impressed and the entire crew I dealt with was amazing! I wish I could get courteous and professional service like that everywhere! Thank you to the whole staff and be proud: you are wonderful!"

## Slide 9
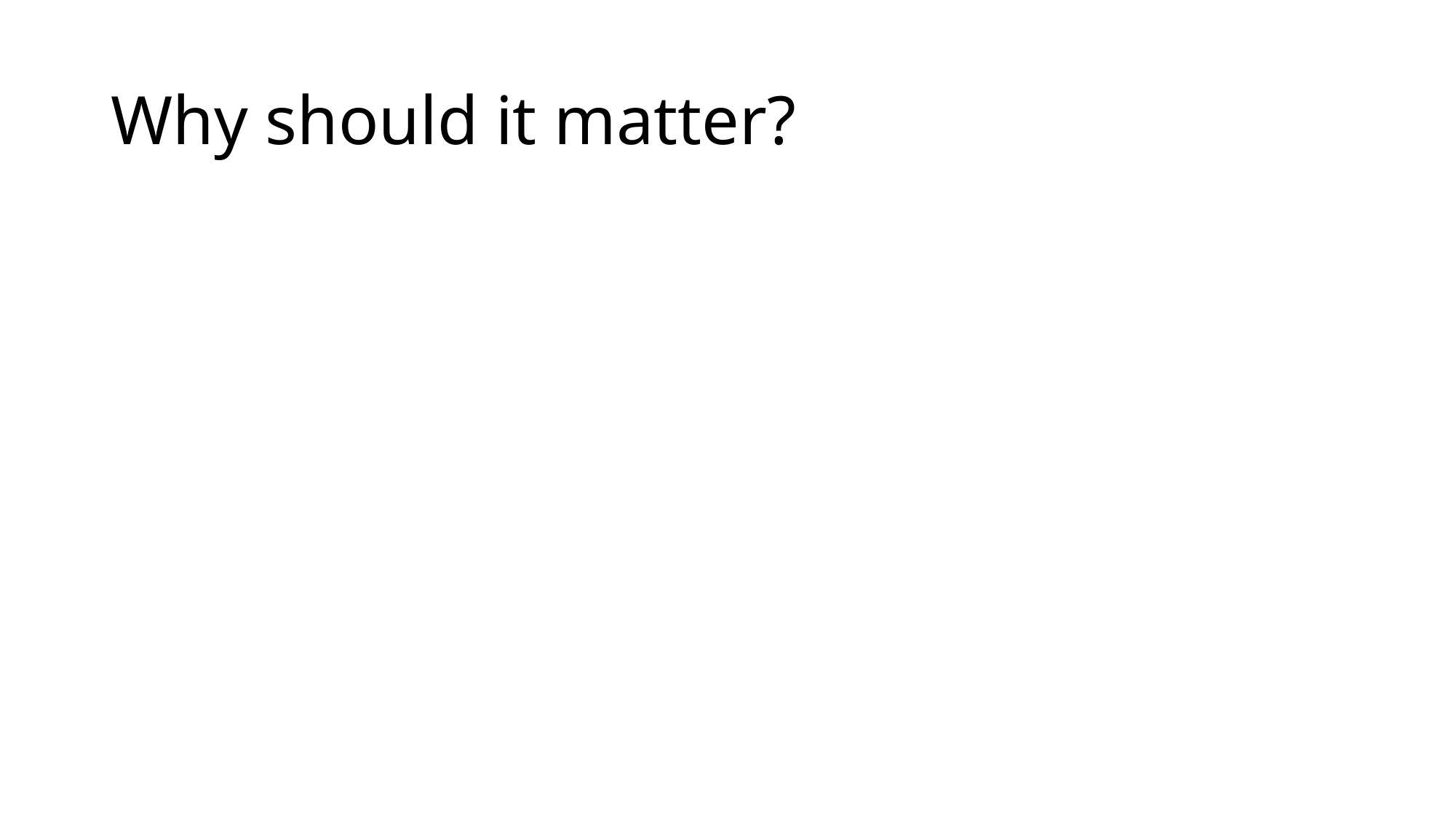

# Why should it matter?

## Slide 10
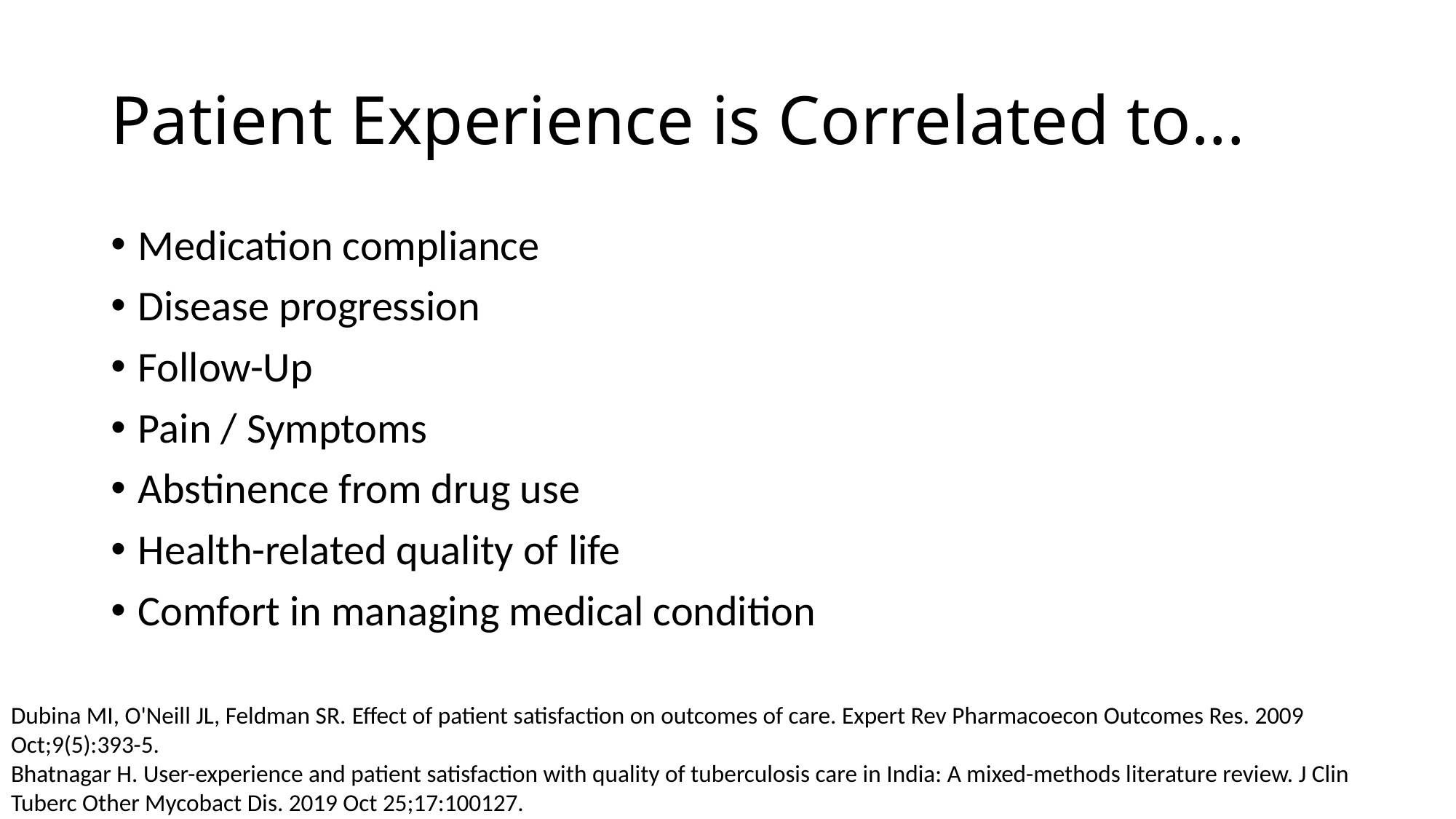

# Patient Experience is Correlated to...
Medication compliance
Disease progression
Follow-Up
Pain / Symptoms
Abstinence from drug use
Health-related quality of life
Comfort in managing medical condition
Dubina MI, O'Neill JL, Feldman SR. Effect of patient satisfaction on outcomes of care. Expert Rev Pharmacoecon Outcomes Res. 2009 Oct;9(5):393-5.
Bhatnagar H. User-experience and patient satisfaction with quality of tuberculosis care in India: A mixed-methods literature review. J Clin Tuberc Other Mycobact Dis. 2019 Oct 25;17:100127.

## Slide 11
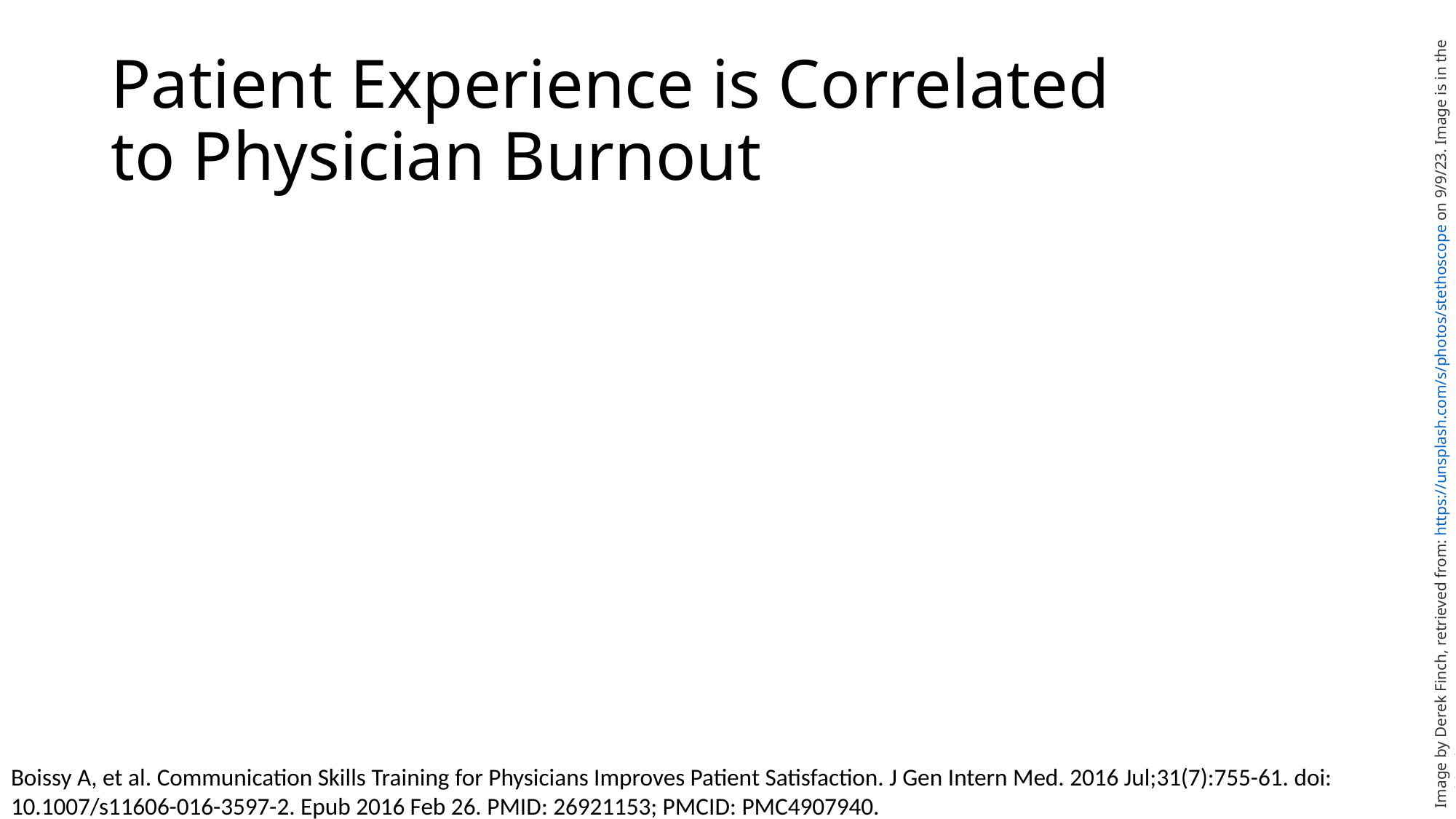

# Patient Experience is Correlated to Physician Burnout
Image by Derek Finch, retrieved from: https://unsplash.com/s/photos/stethoscope on 9/9/23. Image is in the public domain.
Boissy A, et al. Communication Skills Training for Physicians Improves Patient Satisfaction. J Gen Intern Med. 2016 Jul;31(7):755-61. doi: 10.1007/s11606-016-3597-2. Epub 2016 Feb 26. PMID: 26921153; PMCID: PMC4907940.

## Slide 12
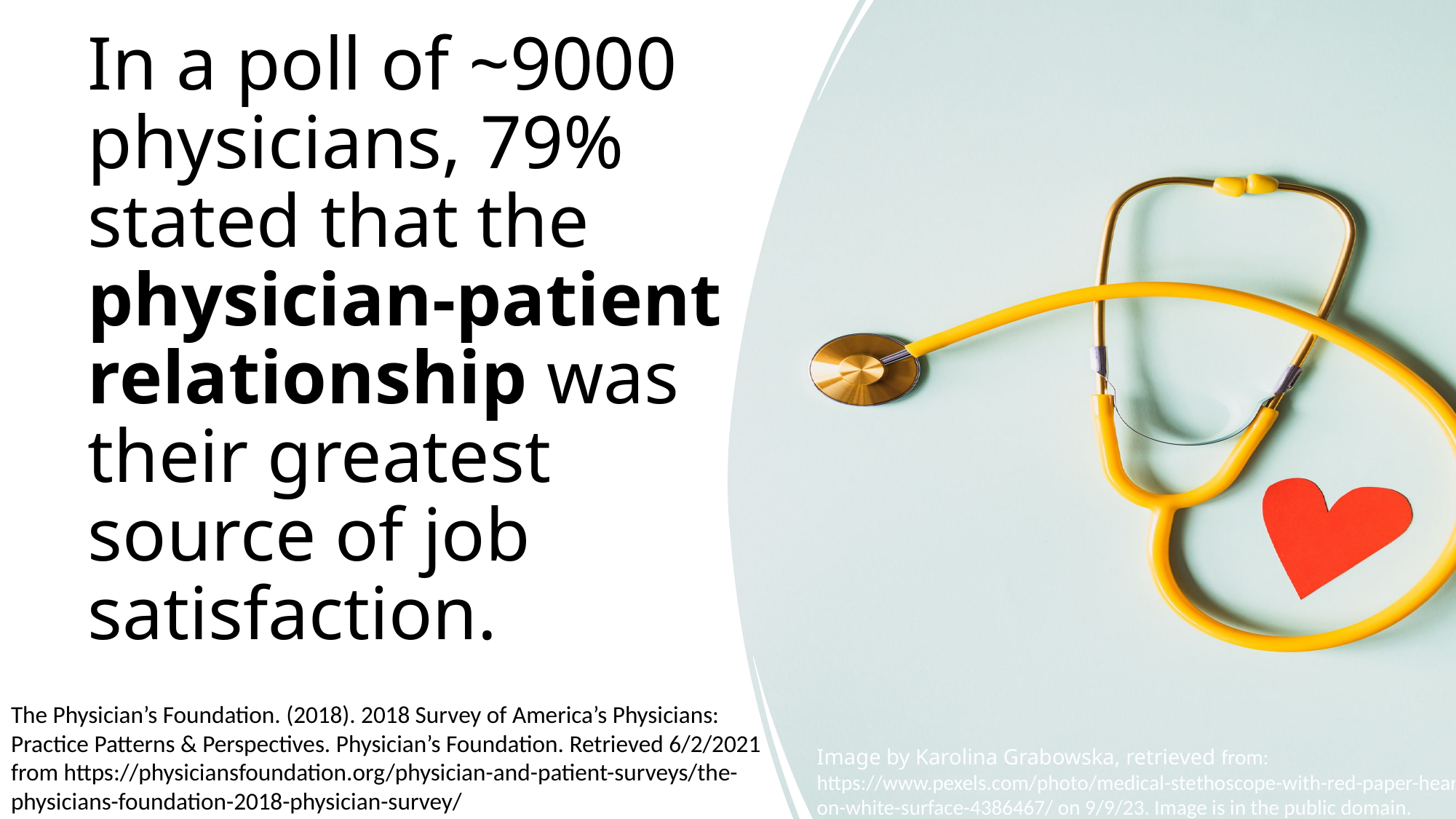

# In a poll of ~9000 physicians, 79% stated that the physician-patient relationship was their greatest source of job satisfaction.
The Physician’s Foundation. (2018). 2018 Survey of America’s Physicians: Practice Patterns & Perspectives. Physician’s Foundation. Retrieved 6/2/2021 from https://physiciansfoundation.org/physician-and-patient-surveys/the-physicians-foundation-2018-physician-survey/
Image by Karolina Grabowska, retrieved from: https://www.pexels.com/photo/medical-stethoscope-with-red-paper-heart-on-white-surface-4386467/ on 9/9/23. Image is in the public domain.

## Slide 13
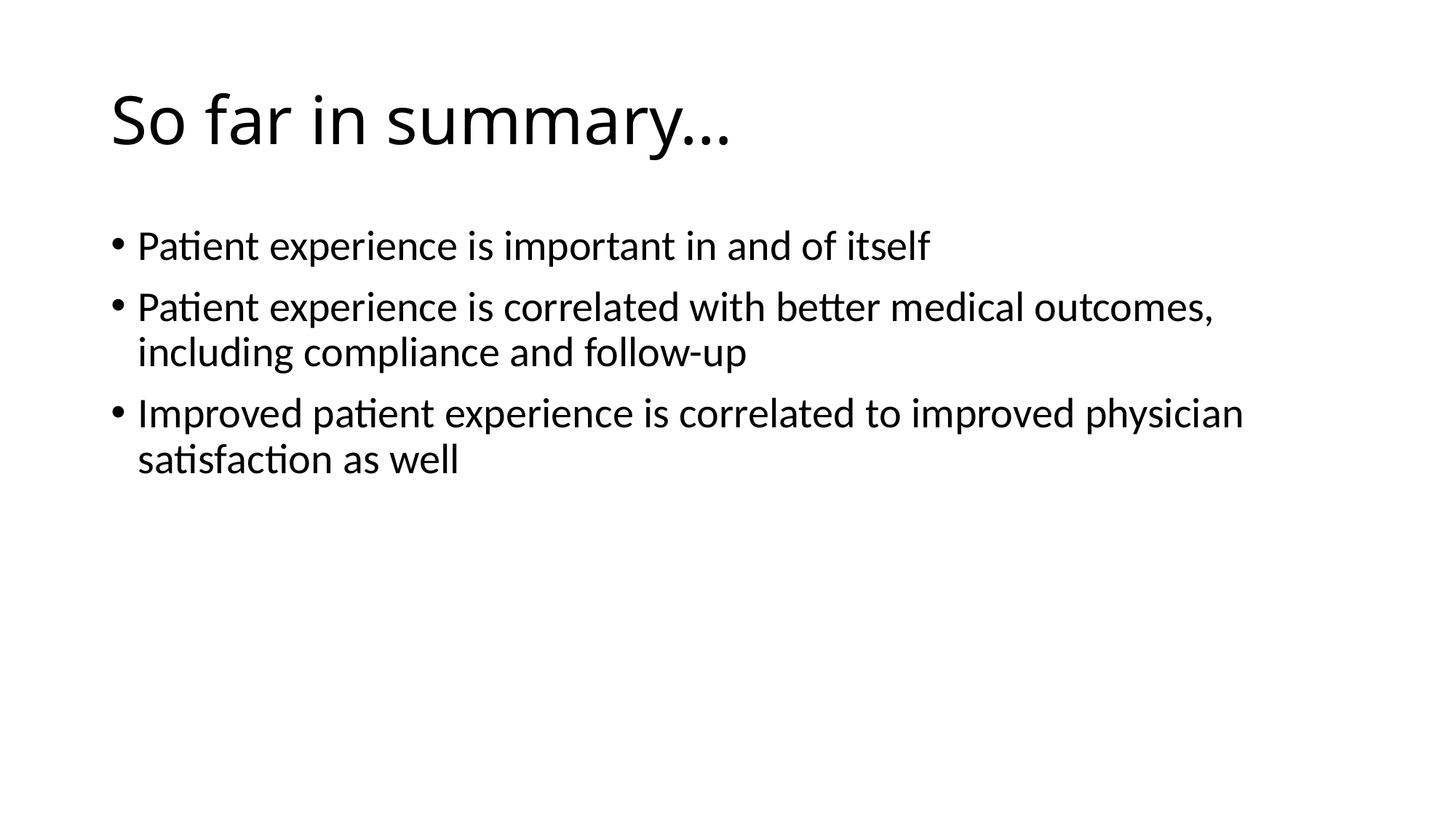

# So far in summary…
Patient experience is important in and of itself
Patient experience is correlated with better medical outcomes, including compliance and follow-up
Improved patient experience is correlated to improved physician satisfaction as well

## Slide 14
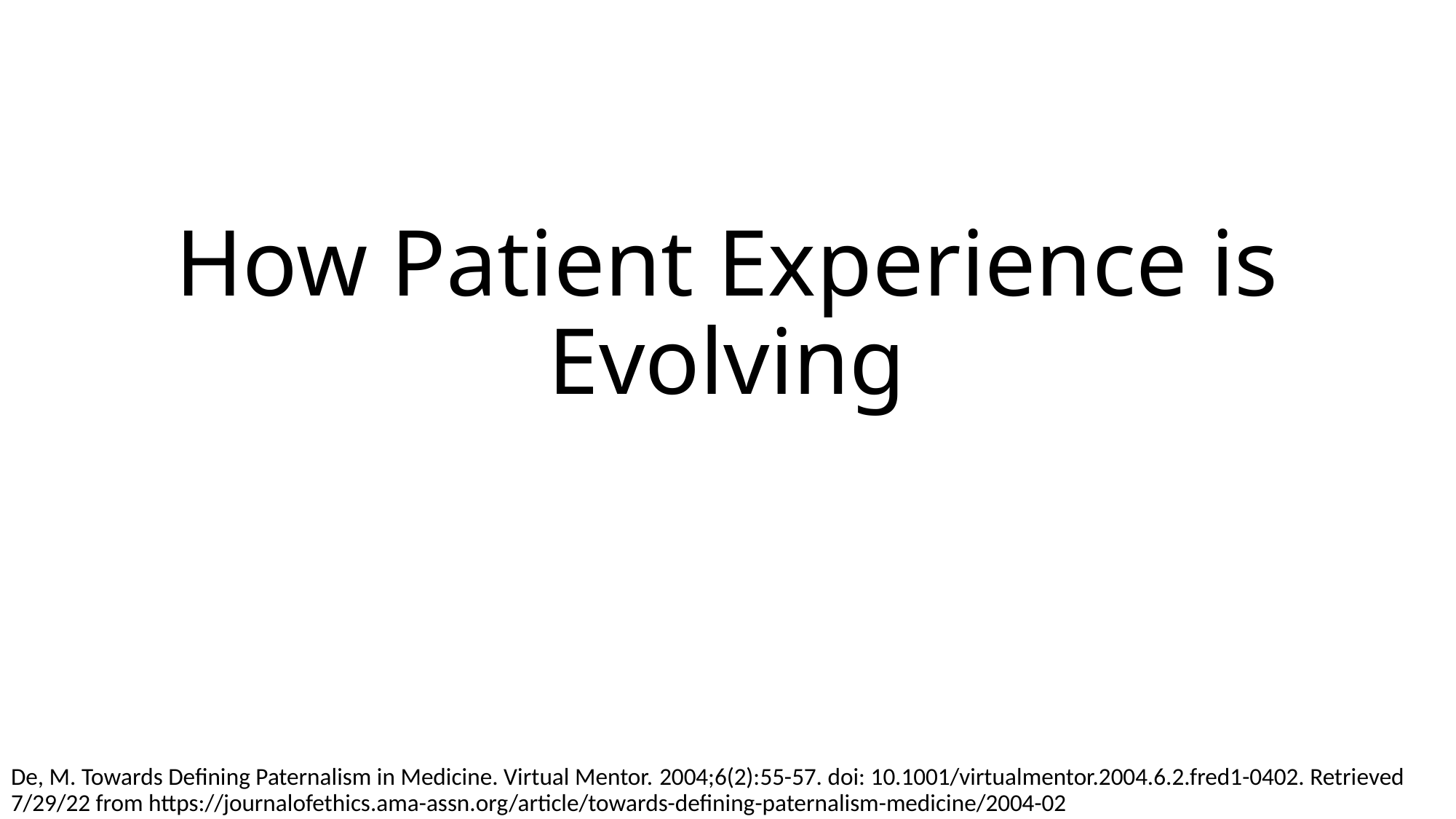

# How Patient Experience is Evolving
De, M. Towards Defining Paternalism in Medicine. Virtual Mentor. 2004;6(2):55-57. doi: 10.1001/virtualmentor.2004.6.2.fred1-0402. Retrieved 7/29/22 from https://journalofethics.ama-assn.org/article/towards-defining-paternalism-medicine/2004-02

## Slide 15
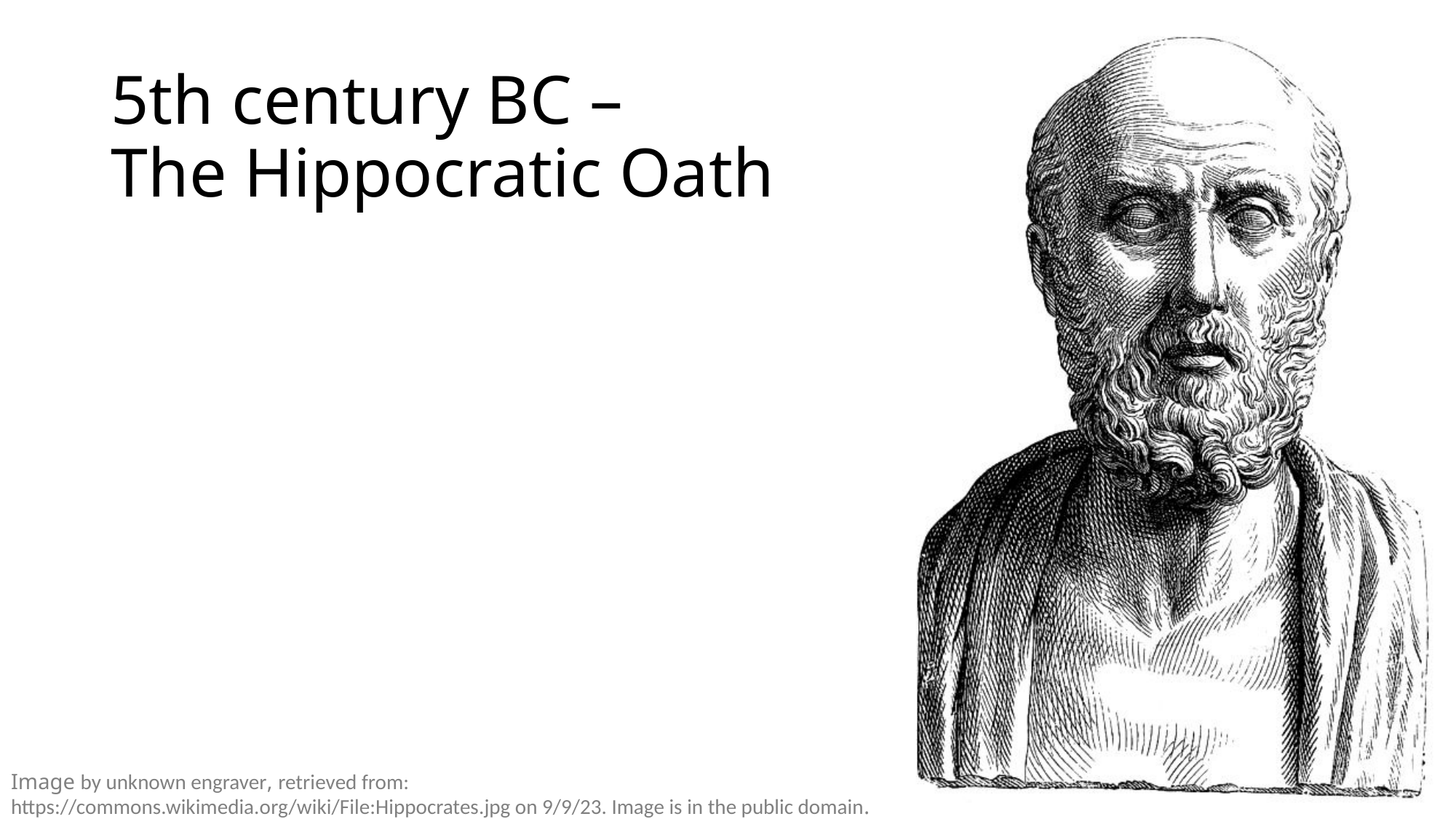

# 5th century BC – The Hippocratic Oath
Image by unknown engraver, retrieved from: https://commons.wikimedia.org/wiki/File:Hippocrates.jpg on 9/9/23. Image is in the public domain.

## Slide 16
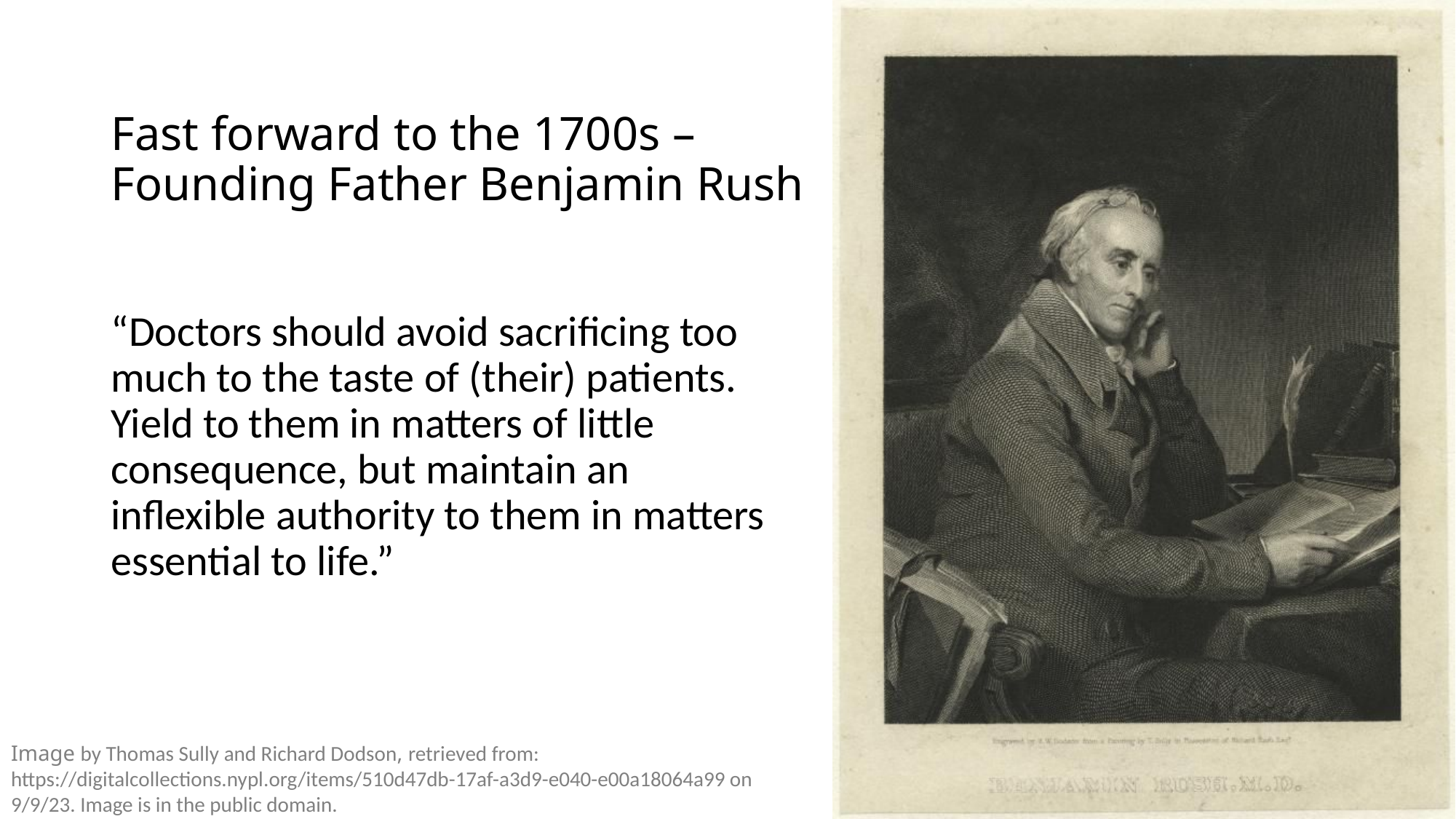

# Fast forward to the 1700s – Founding Father Benjamin Rush
“Doctors should avoid sacrificing too much to the taste of (their) patients. Yield to them in matters of little consequence, but maintain an inflexible authority to them in matters essential to life.”
Image by Thomas Sully and Richard Dodson, retrieved from: https://digitalcollections.nypl.org/items/510d47db-17af-a3d9-e040-e00a18064a99 on 9/9/23. Image is in the public domain.

## Slide 17
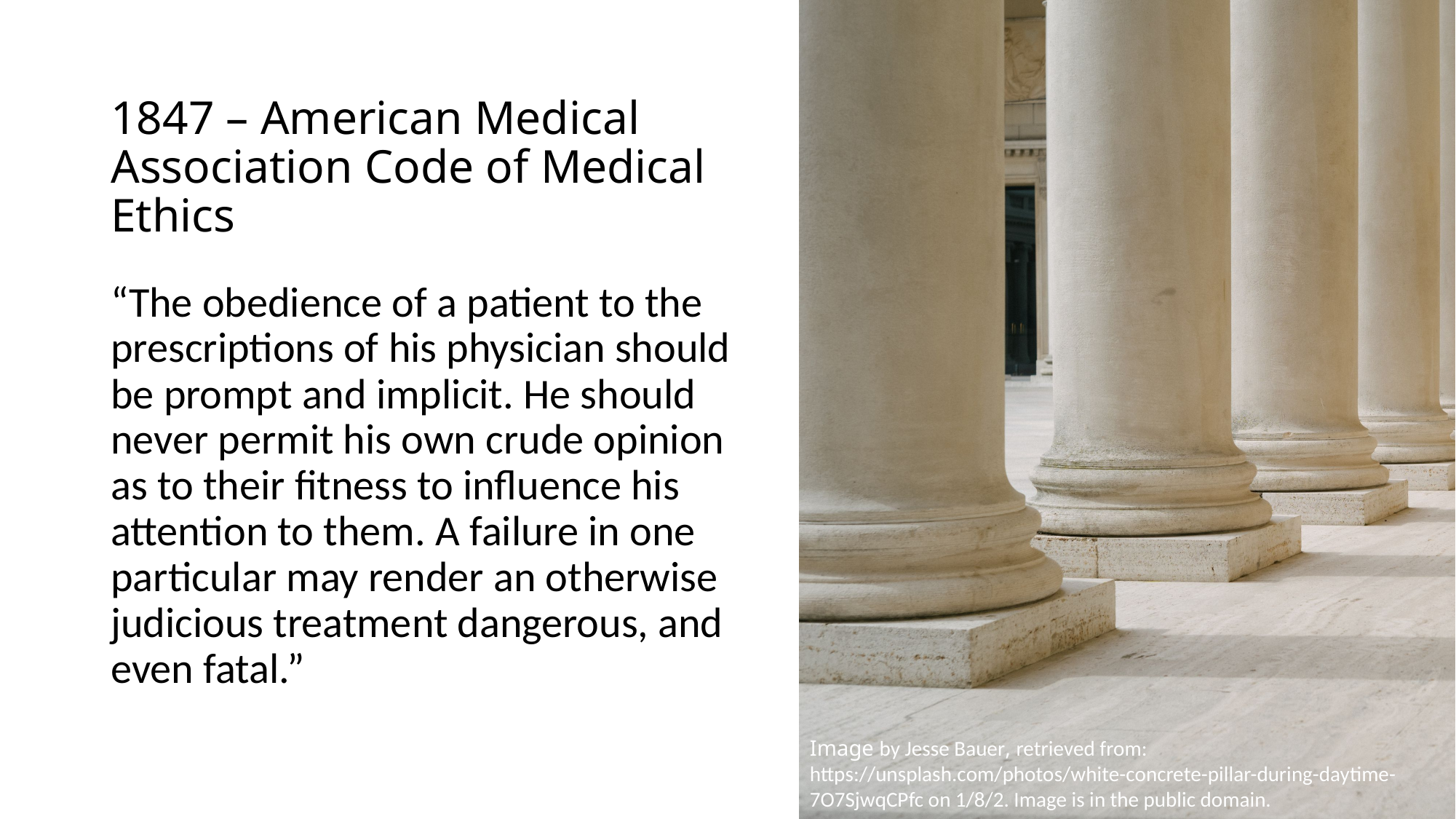

# 1847 – American Medical Association Code of Medical Ethics
“The obedience of a patient to the prescriptions of his physician should be prompt and implicit. He should never permit his own crude opinion as to their fitness to influence his attention to them. A failure in one particular may render an otherwise judicious treatment dangerous, and even fatal.”
Image by Jesse Bauer, retrieved from: https://unsplash.com/photos/white-concrete-pillar-during-daytime-7O7SjwqCPfc on 1/8/2. Image is in the public domain.

## Slide 18
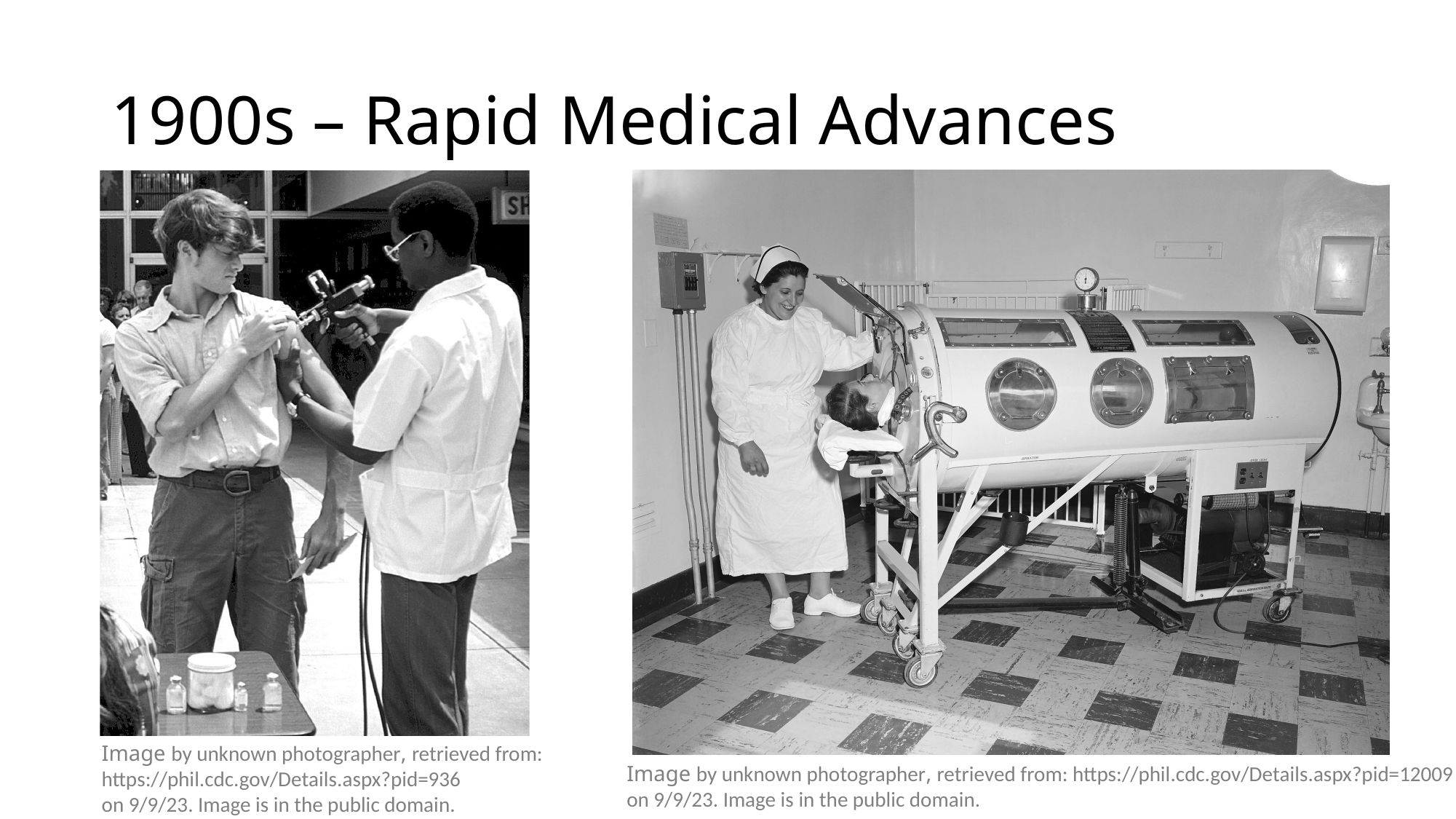

# 1900s – Rapid Medical Advances
Image by unknown photographer, retrieved from: https://phil.cdc.gov/Details.aspx?pid=936
on 9/9/23. Image is in the public domain.
Image by unknown photographer, retrieved from: https://phil.cdc.gov/Details.aspx?pid=12009
on 9/9/23. Image is in the public domain.

## Slide 19
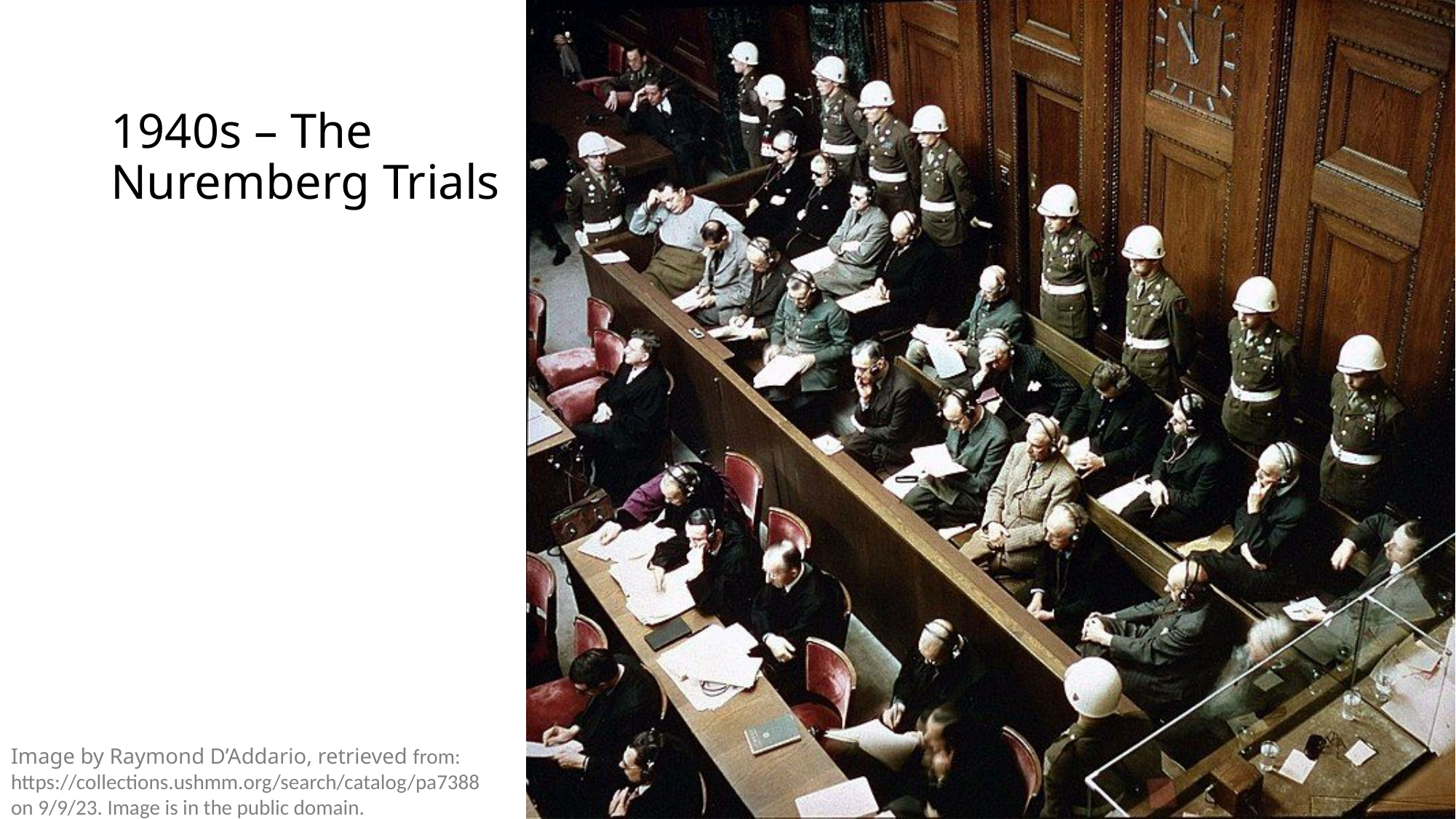

# 1940s – The Nuremberg Trials
Image by Raymond D’Addario, retrieved from: https://collections.ushmm.org/search/catalog/pa7388
on 9/9/23. Image is in the public domain.

## Slide 20
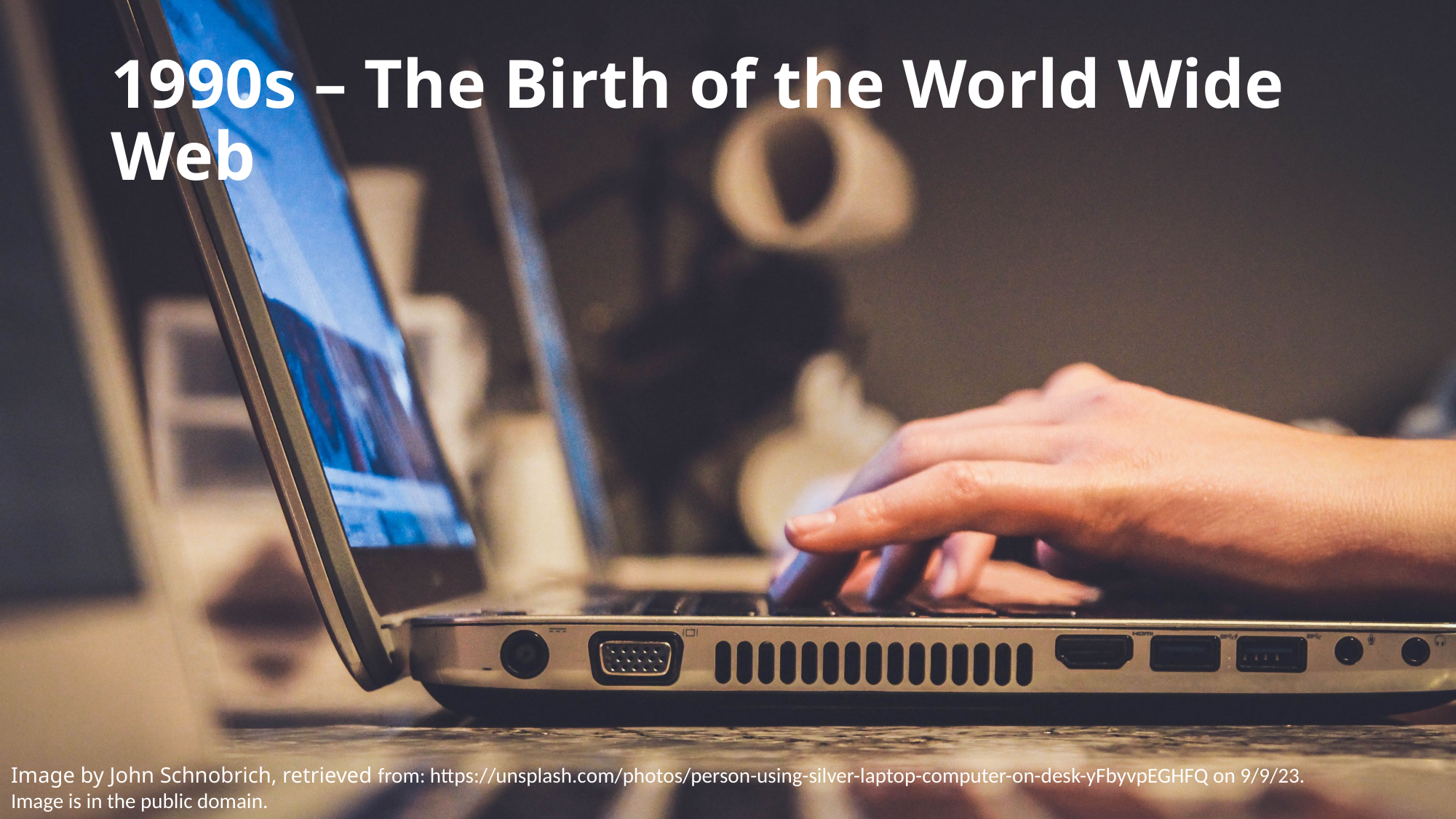

# 1990s – The Birth of the World Wide Web
Image by John Schnobrich, retrieved from: https://unsplash.com/photos/person-using-silver-laptop-computer-on-desk-yFbyvpEGHFQ on 9/9/23. Image is in the public domain.

## Slide 21
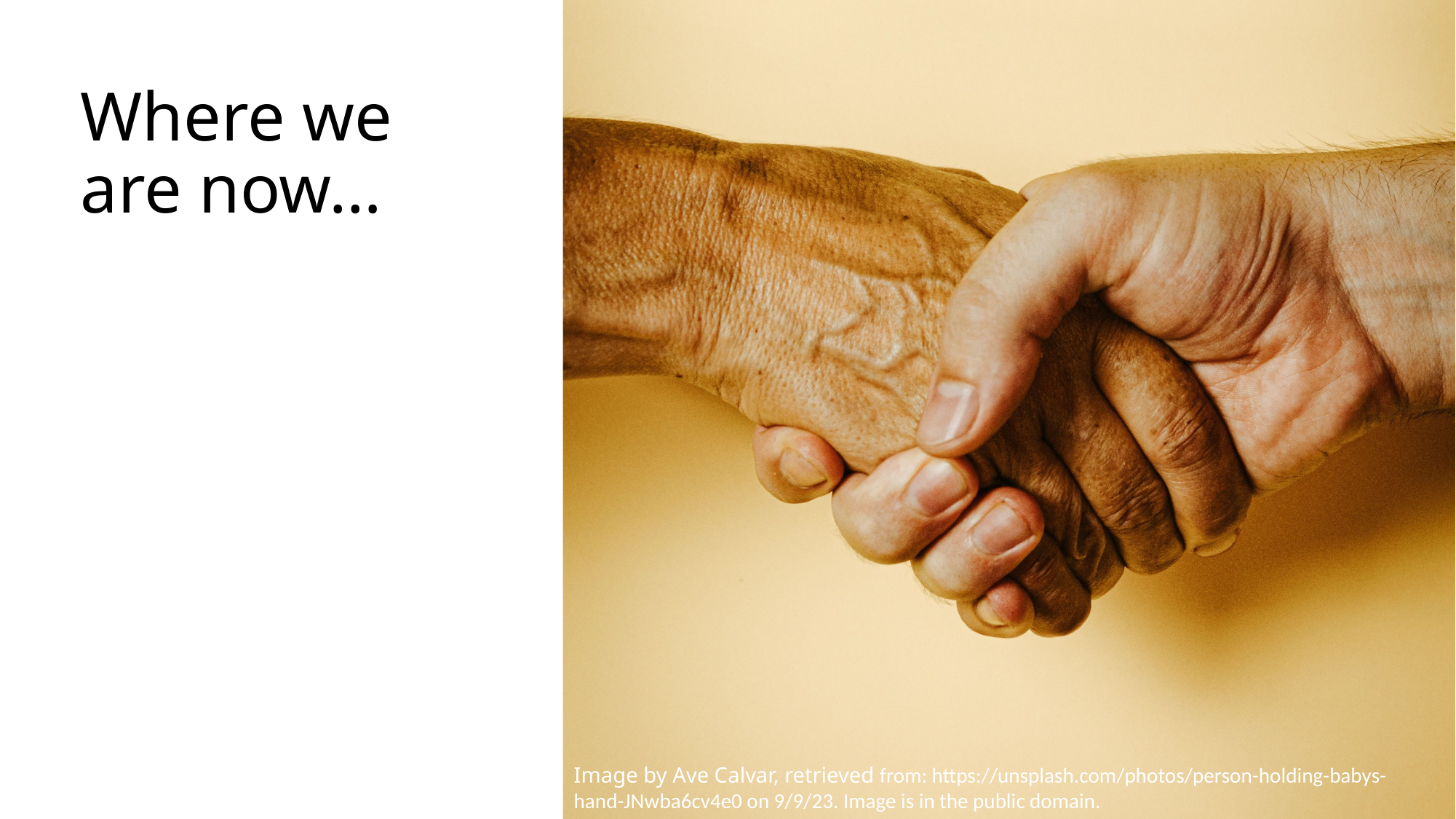

# Where we are now…
Image by Ave Calvar, retrieved from: https://unsplash.com/photos/person-holding-babys-hand-JNwba6cv4e0 on 9/9/23. Image is in the public domain.

## Slide 22
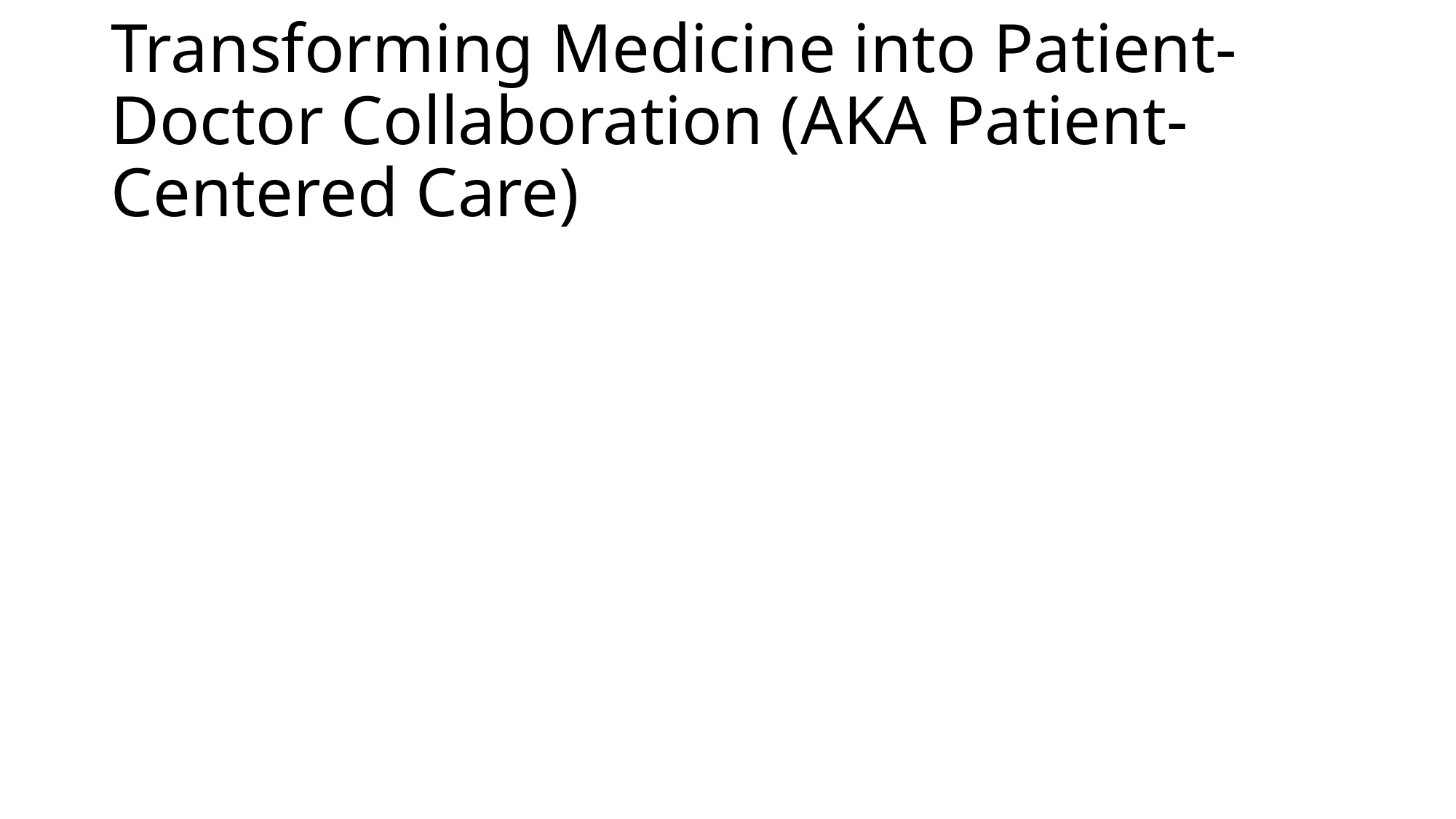

# Transforming Medicine into Patient-Doctor Collaboration (AKA Patient-Centered Care)

## Slide 23
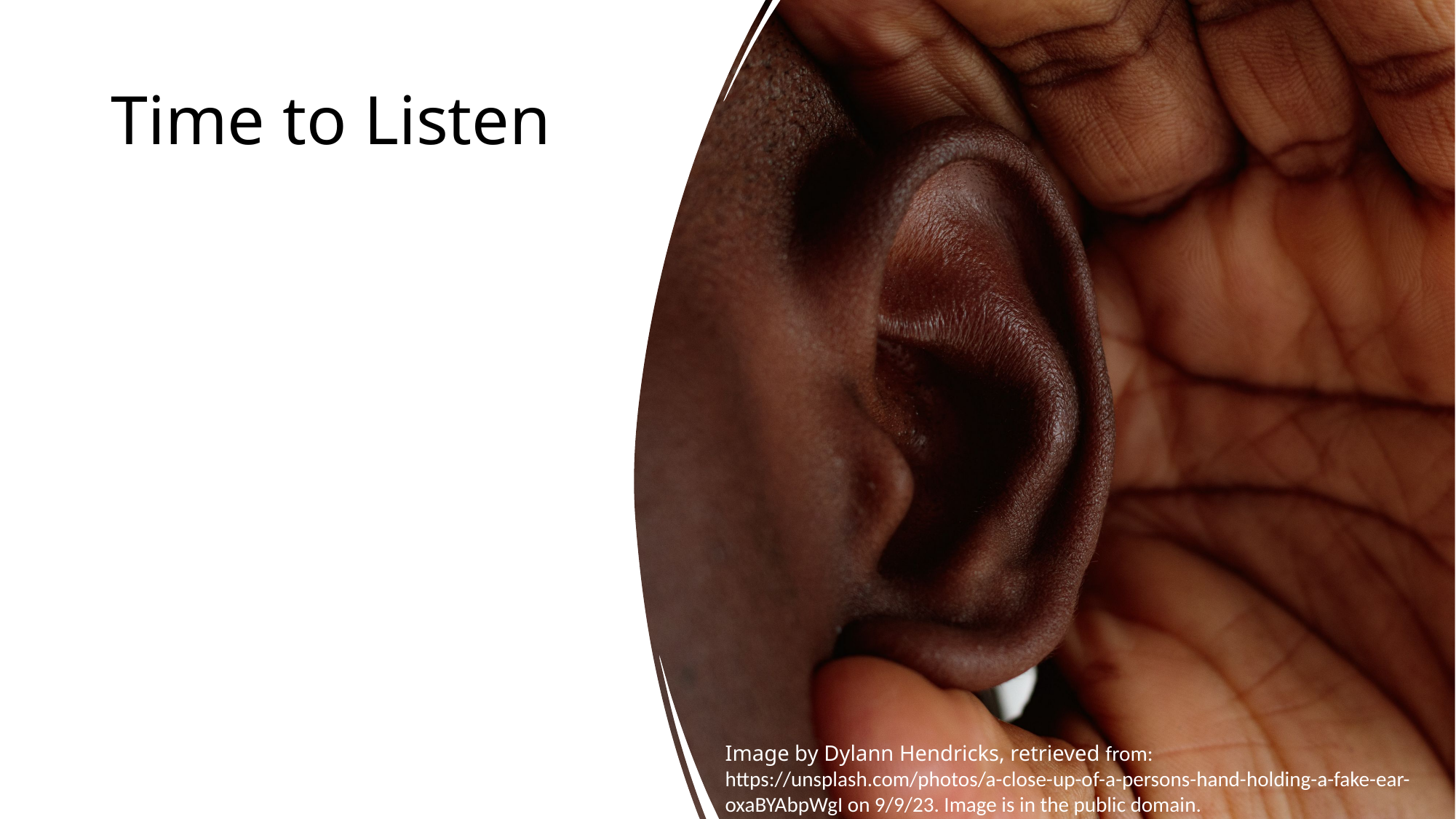

# Time to Listen
Image by Dylann Hendricks, retrieved from: https://unsplash.com/photos/a-close-up-of-a-persons-hand-holding-a-fake-ear-oxaBYAbpWgI on 9/9/23. Image is in the public domain.

## Slide 24
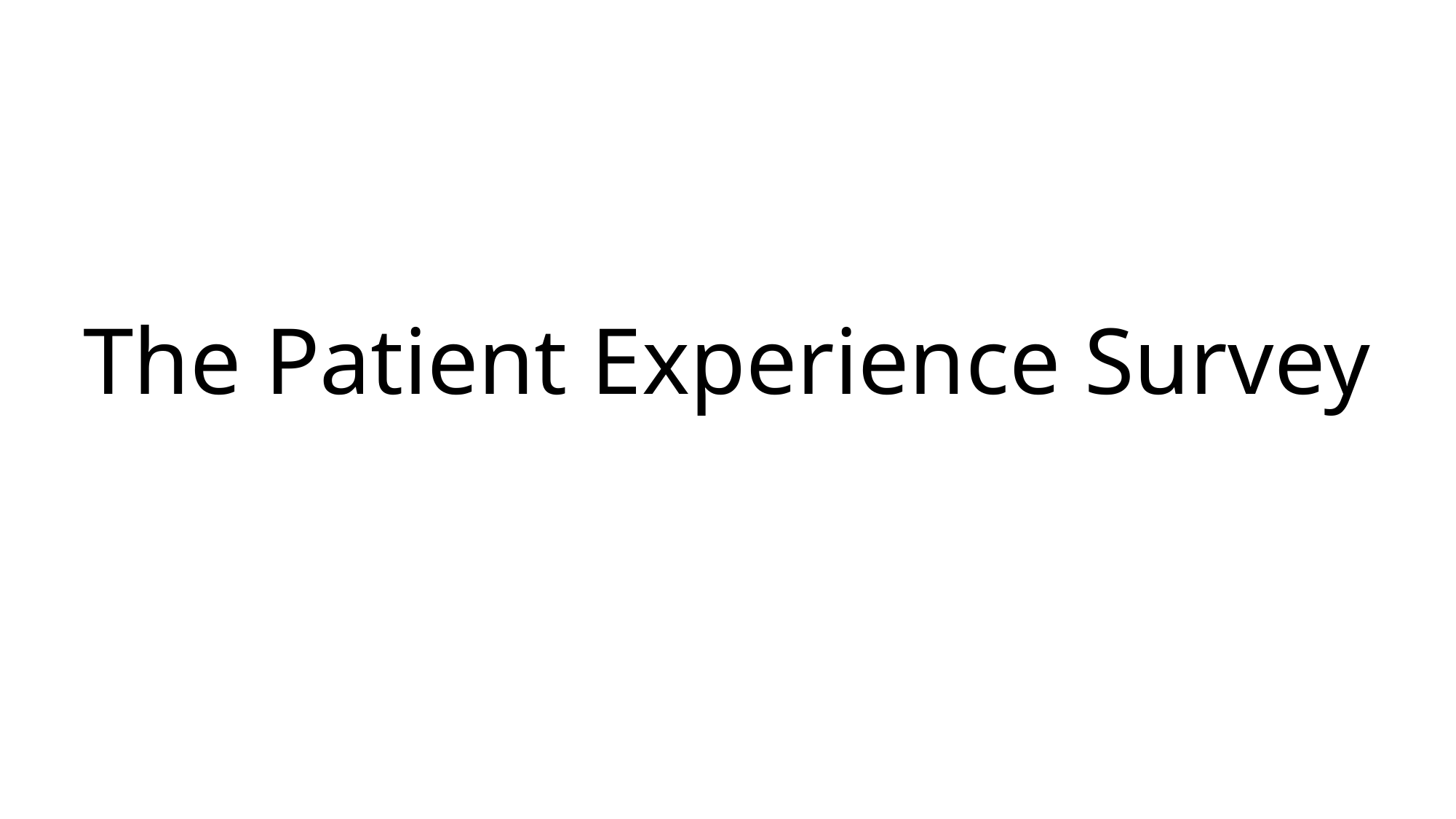

# The Patient Experience Survey

## Slide 25
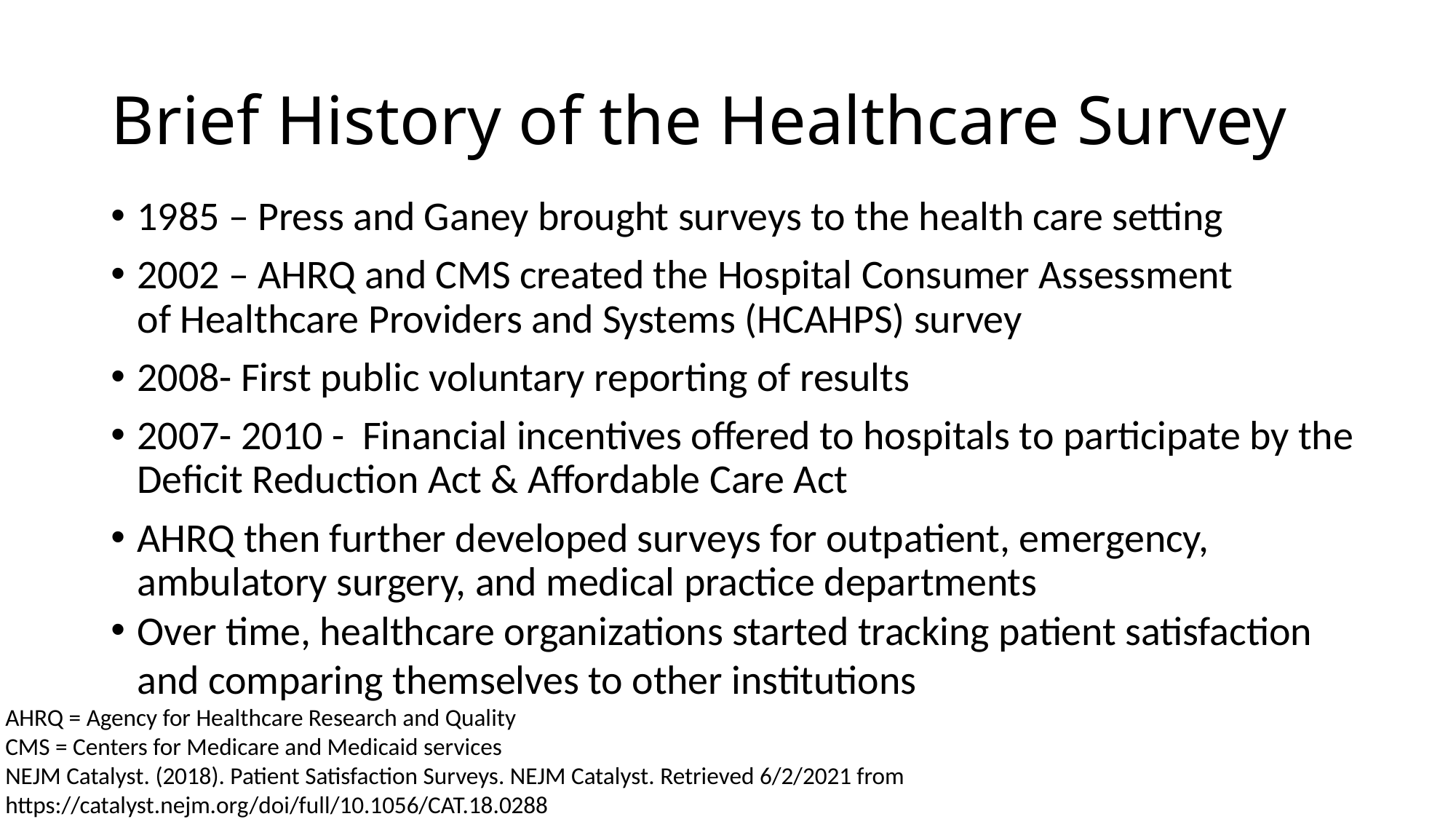

# Brief History of the Healthcare Survey
1985 – Press and Ganey brought surveys to the health care setting
2002 – AHRQ and CMS created the Hospital Consumer Assessment of Healthcare Providers and Systems (HCAHPS) survey
2008- First public voluntary reporting of results
2007- 2010 -  Financial incentives offered to hospitals to participate by the Deficit Reduction Act & Affordable Care Act
AHRQ then further developed surveys for outpatient, emergency, ambulatory surgery, and medical practice departments
Over time, healthcare organizations started tracking patient satisfaction and comparing themselves to other institutions
AHRQ = Agency for Healthcare Research and Quality
CMS = Centers for Medicare and Medicaid services
NEJM Catalyst. (2018). Patient Satisfaction Surveys. NEJM Catalyst. Retrieved 6/2/2021 from https://catalyst.nejm.org/doi/full/10.1056/CAT.18.0288

## Slide 26
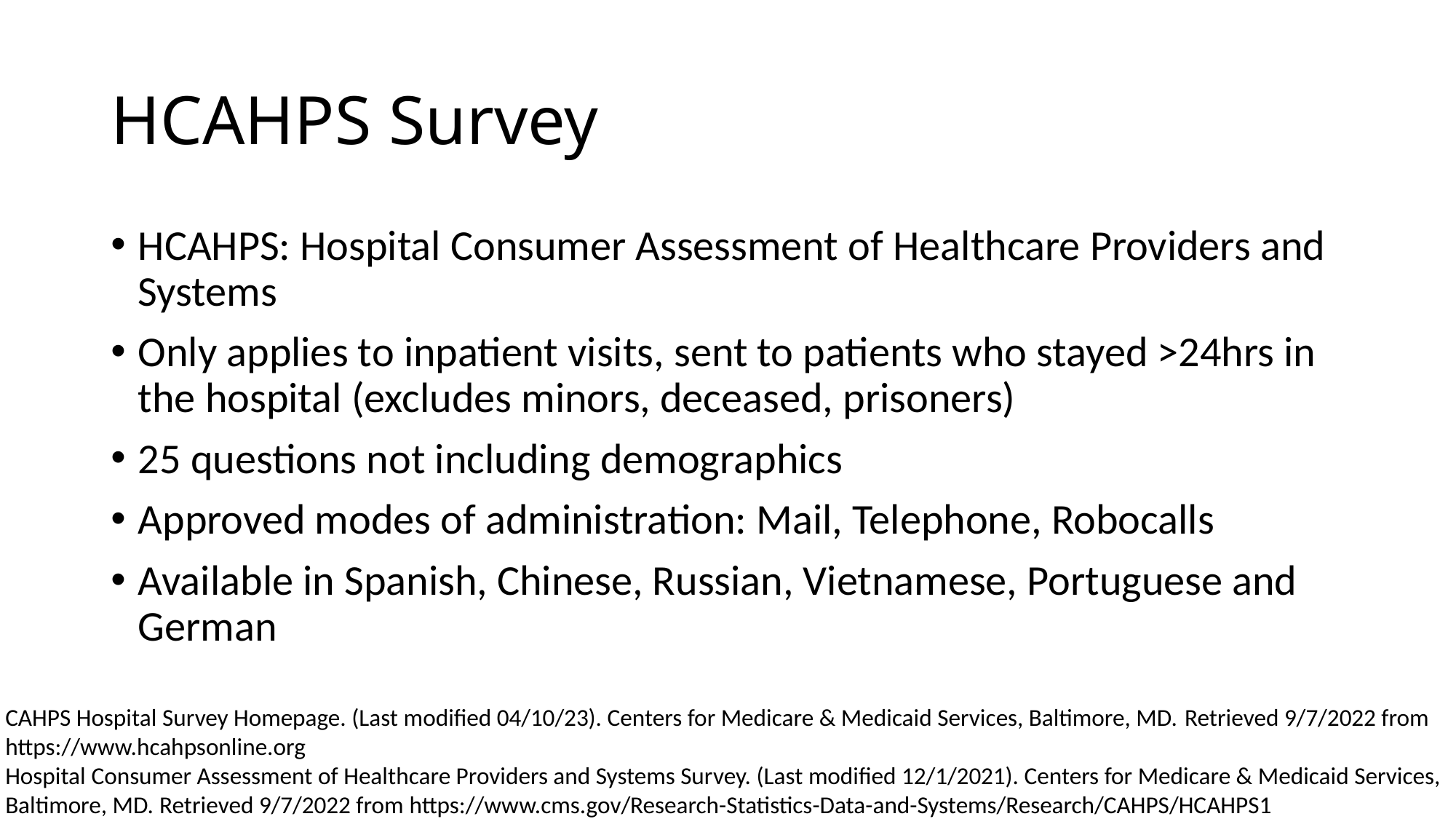

# HCAHPS Survey
HCAHPS: Hospital Consumer Assessment of Healthcare Providers and Systems
Only applies to inpatient visits, sent to patients who stayed >24hrs in the hospital (excludes minors, deceased, prisoners)
25 questions not including demographics
Approved modes of administration: Mail, Telephone, Robocalls
Available in Spanish, Chinese, Russian, Vietnamese, Portuguese and German
CAHPS Hospital Survey Homepage. (Last modified 04/10/23). Centers for Medicare & Medicaid Services, Baltimore, MD. Retrieved 9/7/2022 from https://www.hcahpsonline.org
Hospital Consumer Assessment of Healthcare Providers and Systems Survey. (Last modified 12/1/2021). Centers for Medicare & Medicaid Services, Baltimore, MD. Retrieved 9/7/2022 from https://www.cms.gov/Research-Statistics-Data-and-Systems/Research/CAHPS/HCAHPS1

## Slide 27
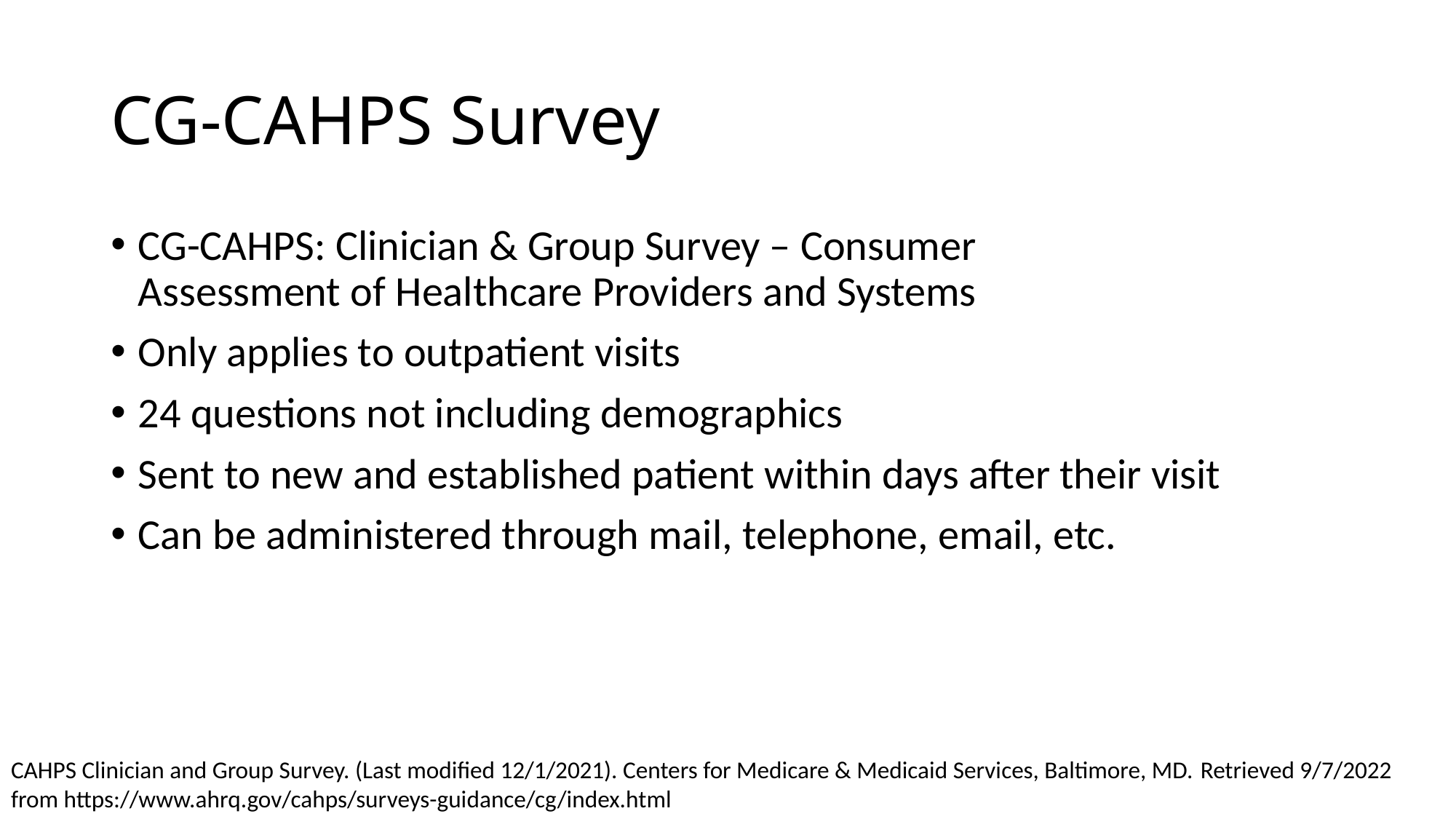

# CG-CAHPS Survey
CG-CAHPS: Clinician & Group Survey – Consumer Assessment of Healthcare Providers and Systems
Only applies to outpatient visits
24 questions not including demographics
Sent to new and established patient within days after their visit
Can be administered through mail, telephone, email, etc.
CAHPS Clinician and Group Survey. (Last modified 12/1/2021). Centers for Medicare & Medicaid Services, Baltimore, MD. Retrieved 9/7/2022 from https://www.ahrq.gov/cahps/surveys-guidance/cg/index.html

## Slide 28
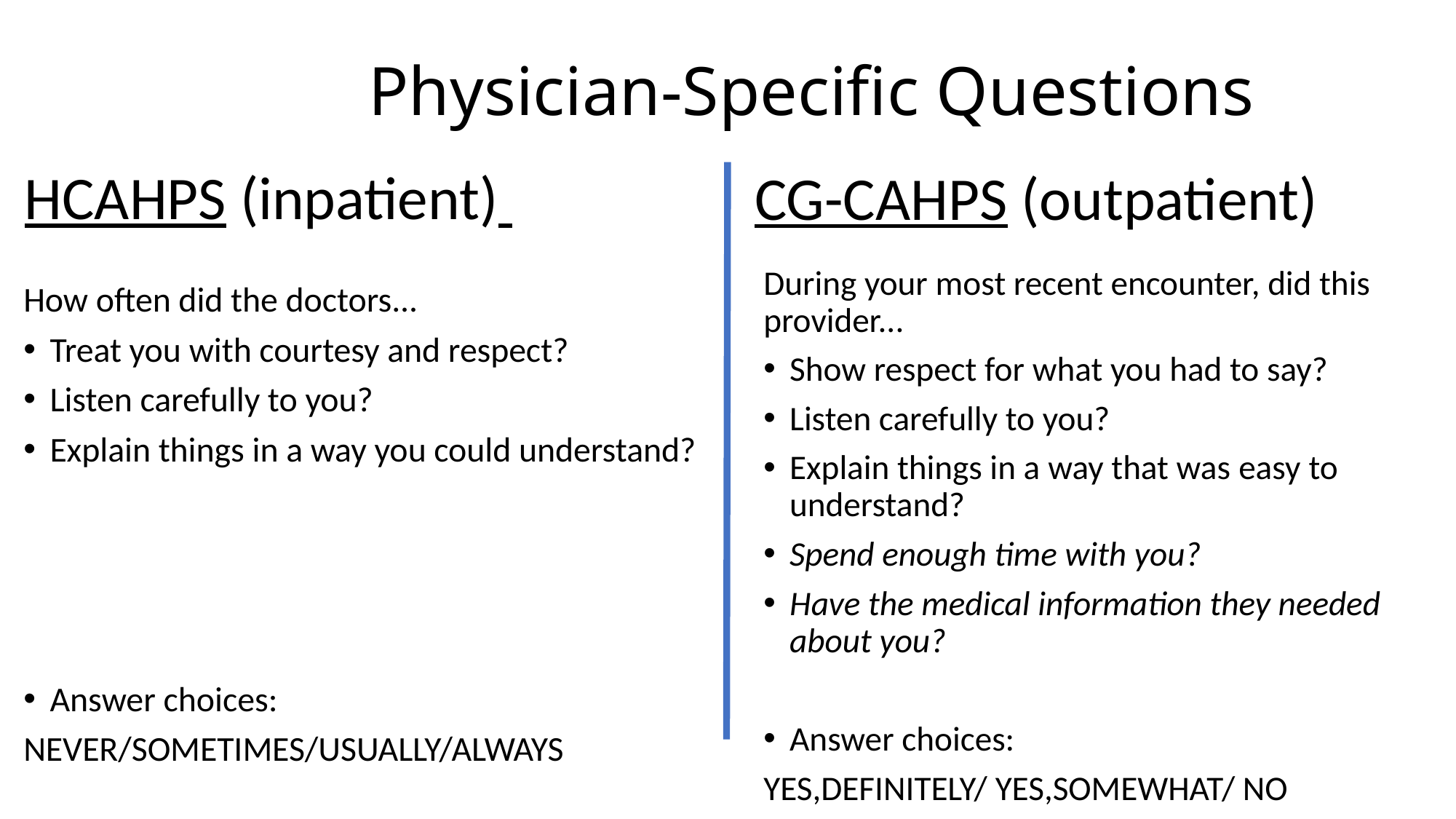

# Physician-Specific Questions
HCAHPS (inpatient)
CG-CAHPS (outpatient)
During your most recent encounter, did this provider...
Show respect for what you had to say?
Listen carefully to you?
Explain things in a way that was easy to understand?
Spend enough time with you?
Have the medical information they needed about you?
Answer choices:
YES,DEFINITELY/ YES,SOMEWHAT/ NO
How often did the doctors...
Treat you with courtesy and respect?
Listen carefully to you?
Explain things in a way you could understand?
Answer choices:
NEVER/SOMETIMES/USUALLY/ALWAYS

## Slide 29
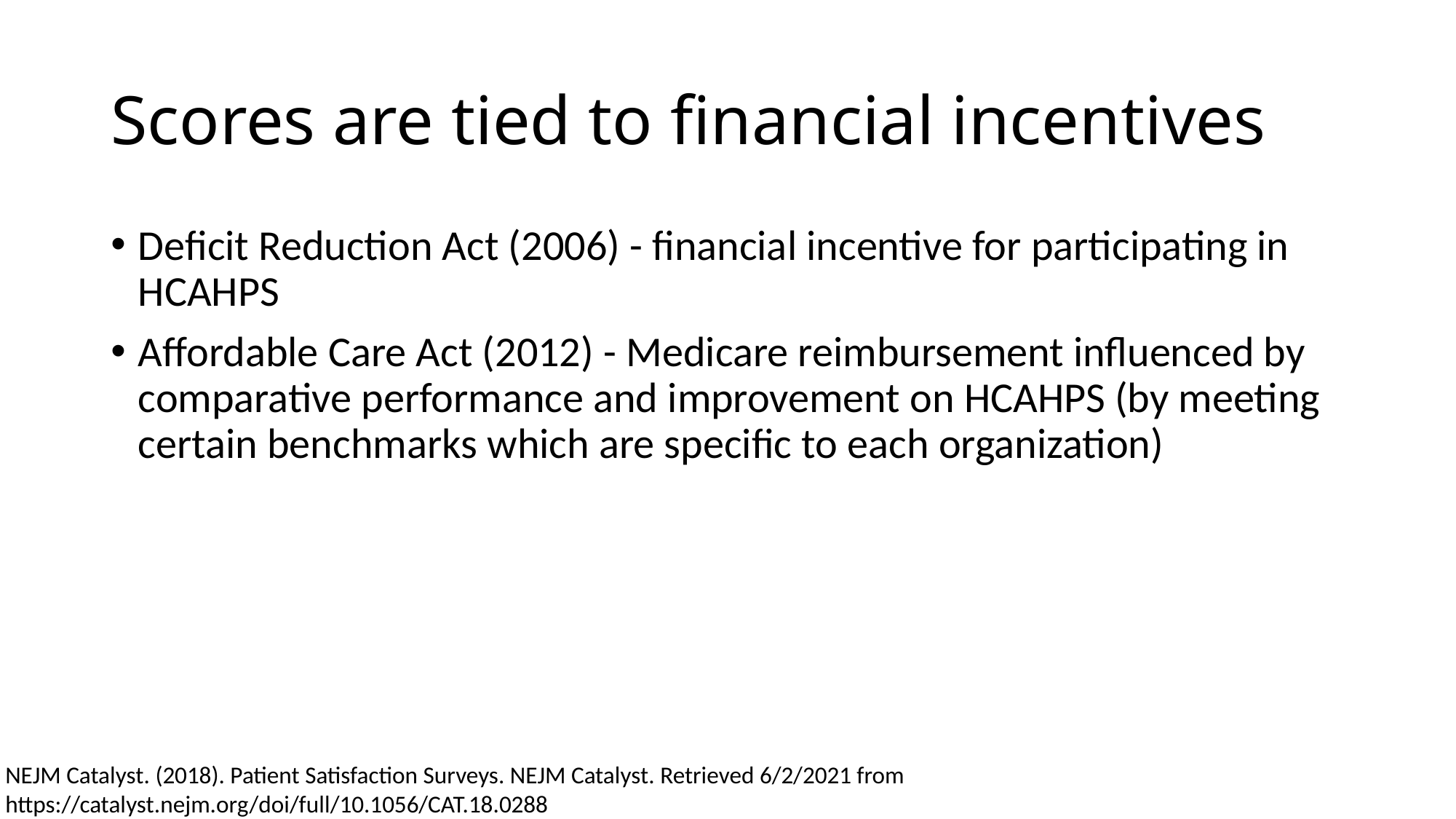

# Scores are tied to financial incentives
Deficit Reduction Act (2006) - financial incentive for participating in HCAHPS
Affordable Care Act (2012) - Medicare reimbursement influenced by comparative performance and improvement on HCAHPS (by meeting certain benchmarks which are specific to each organization)
NEJM Catalyst. (2018). Patient Satisfaction Surveys. NEJM Catalyst. Retrieved 6/2/2021 from https://catalyst.nejm.org/doi/full/10.1056/CAT.18.0288

## Slide 30
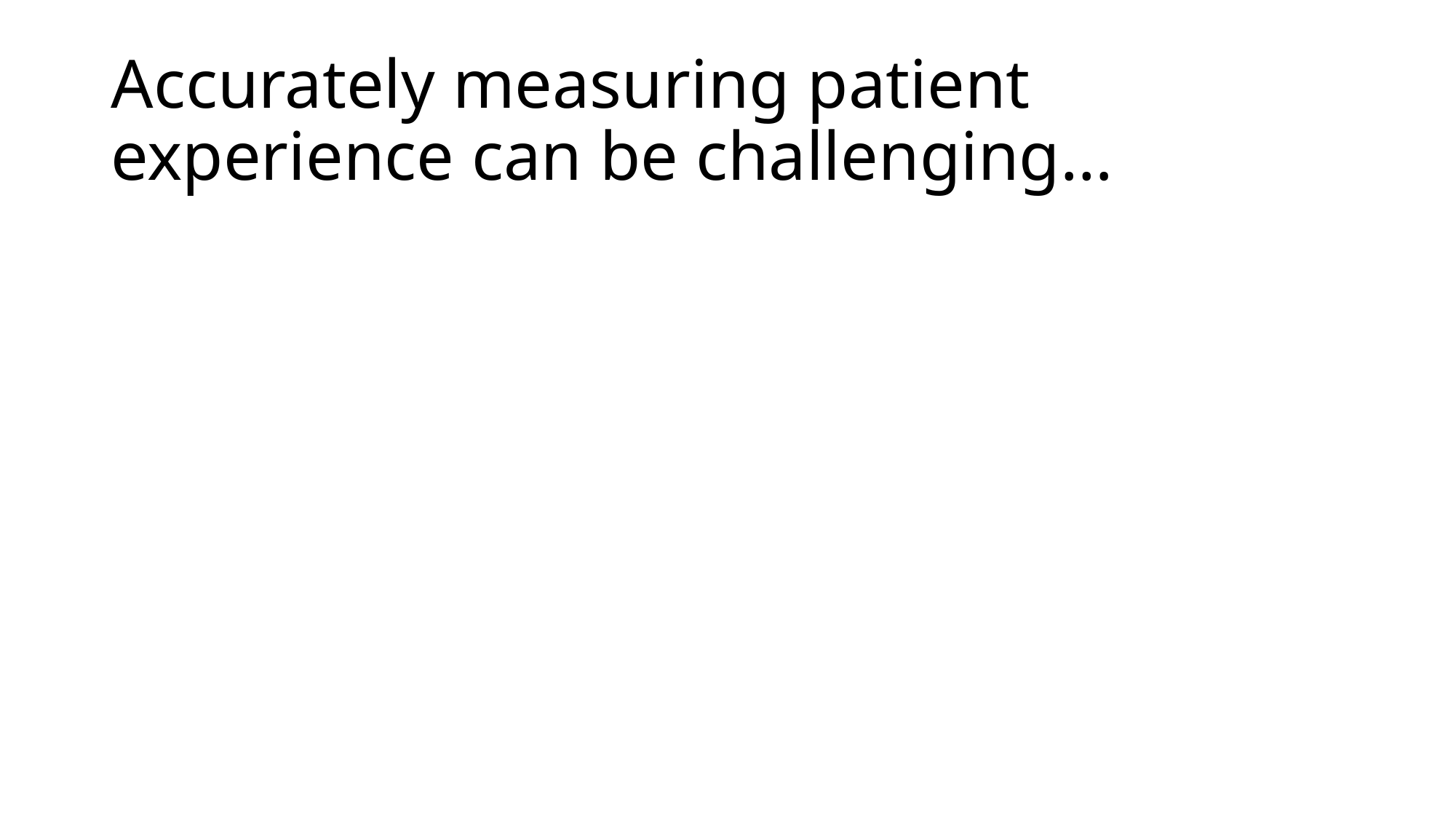

# Accurately measuring patient experience can be challenging…

## Slide 31
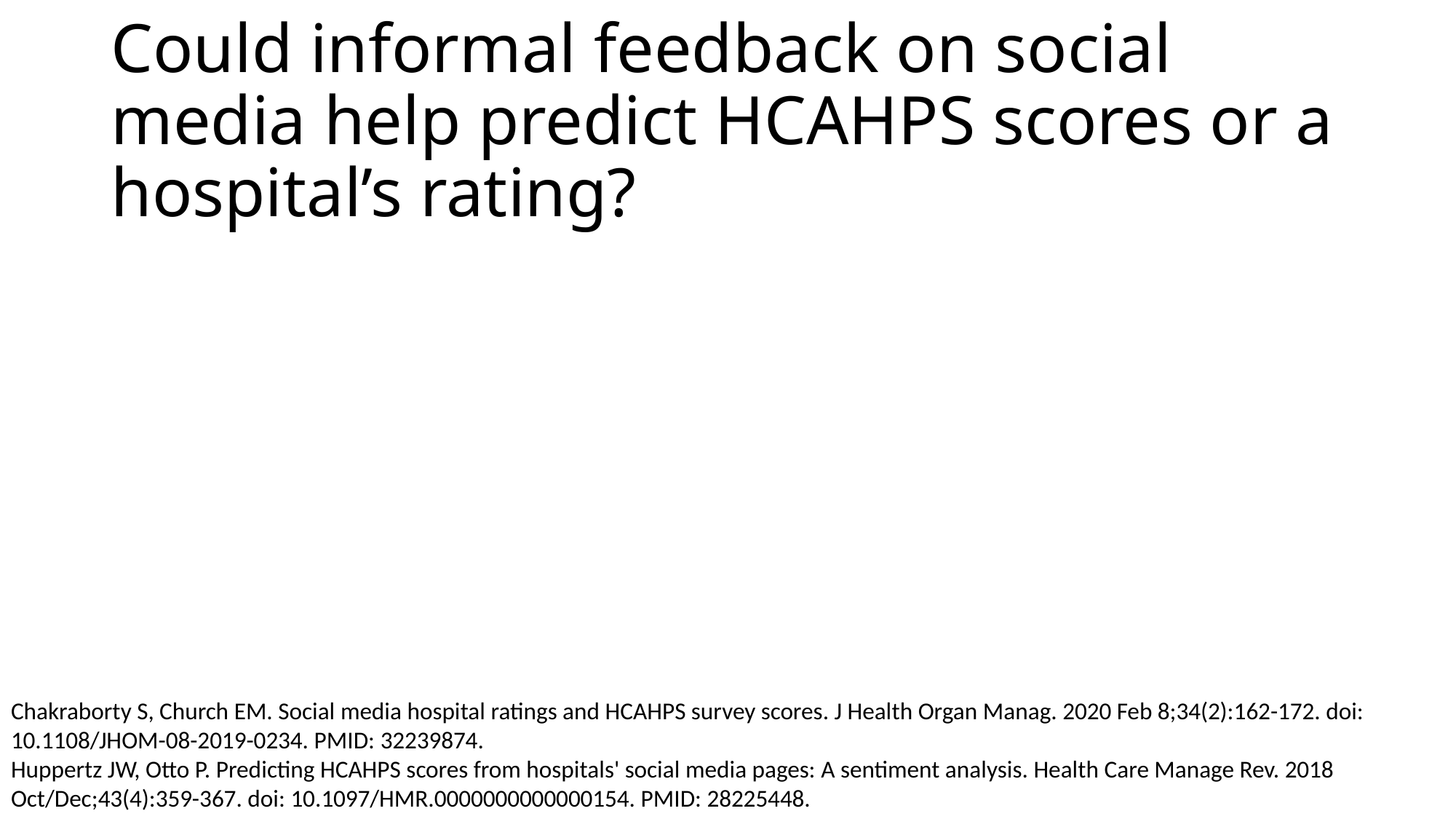

# Could informal feedback on social media help predict HCAHPS scores or a hospital’s rating?
Chakraborty S, Church EM. Social media hospital ratings and HCAHPS survey scores. J Health Organ Manag. 2020 Feb 8;34(2):162-172. doi: 10.1108/JHOM-08-2019-0234. PMID: 32239874.
Huppertz JW, Otto P. Predicting HCAHPS scores from hospitals' social media pages: A sentiment analysis. Health Care Manage Rev. 2018 Oct/Dec;43(4):359-367. doi: 10.1097/HMR.0000000000000154. PMID: 28225448.

## Slide 32
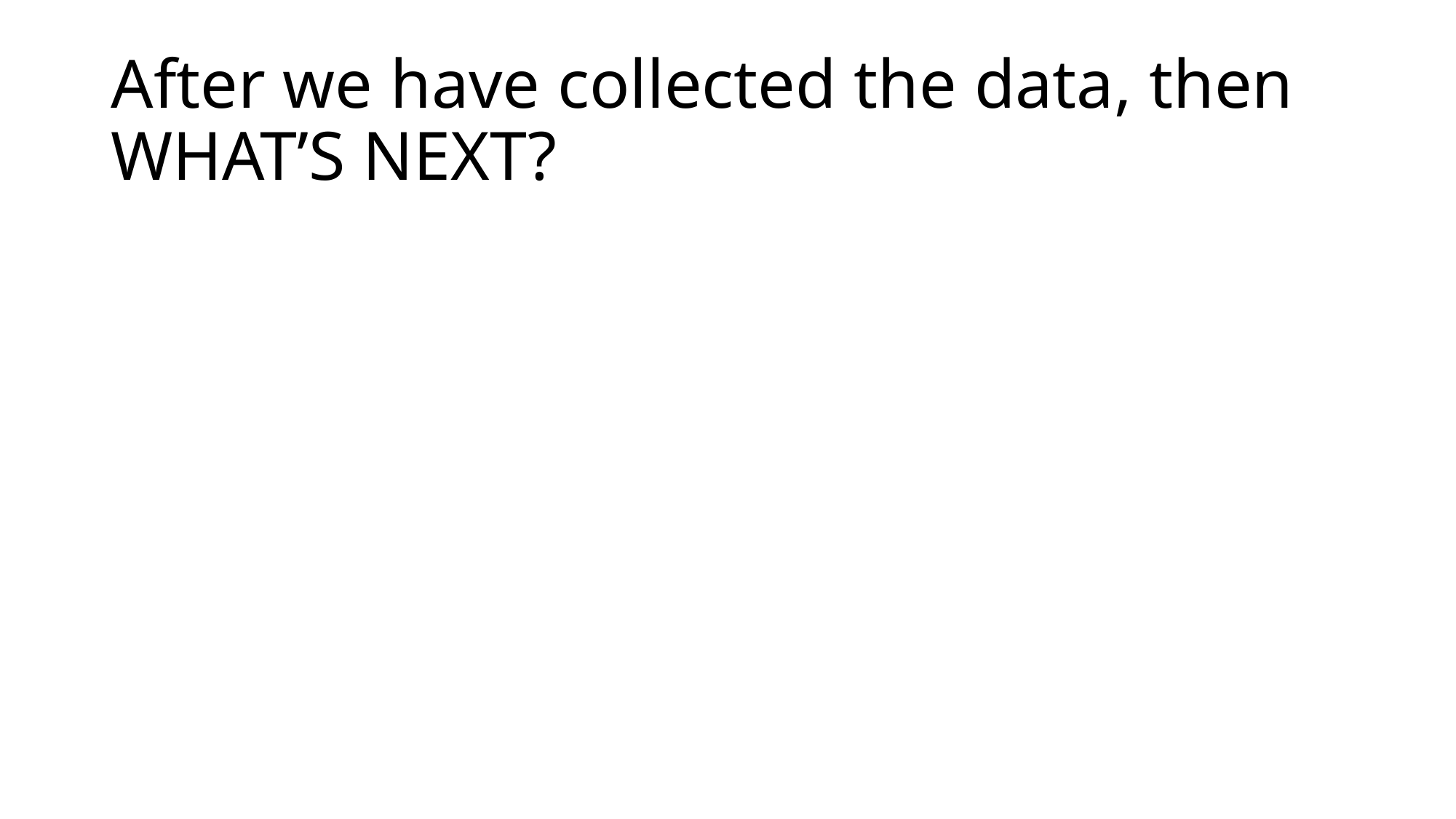

# After we have collected the data, then WHAT’S NEXT?

## Slide 33
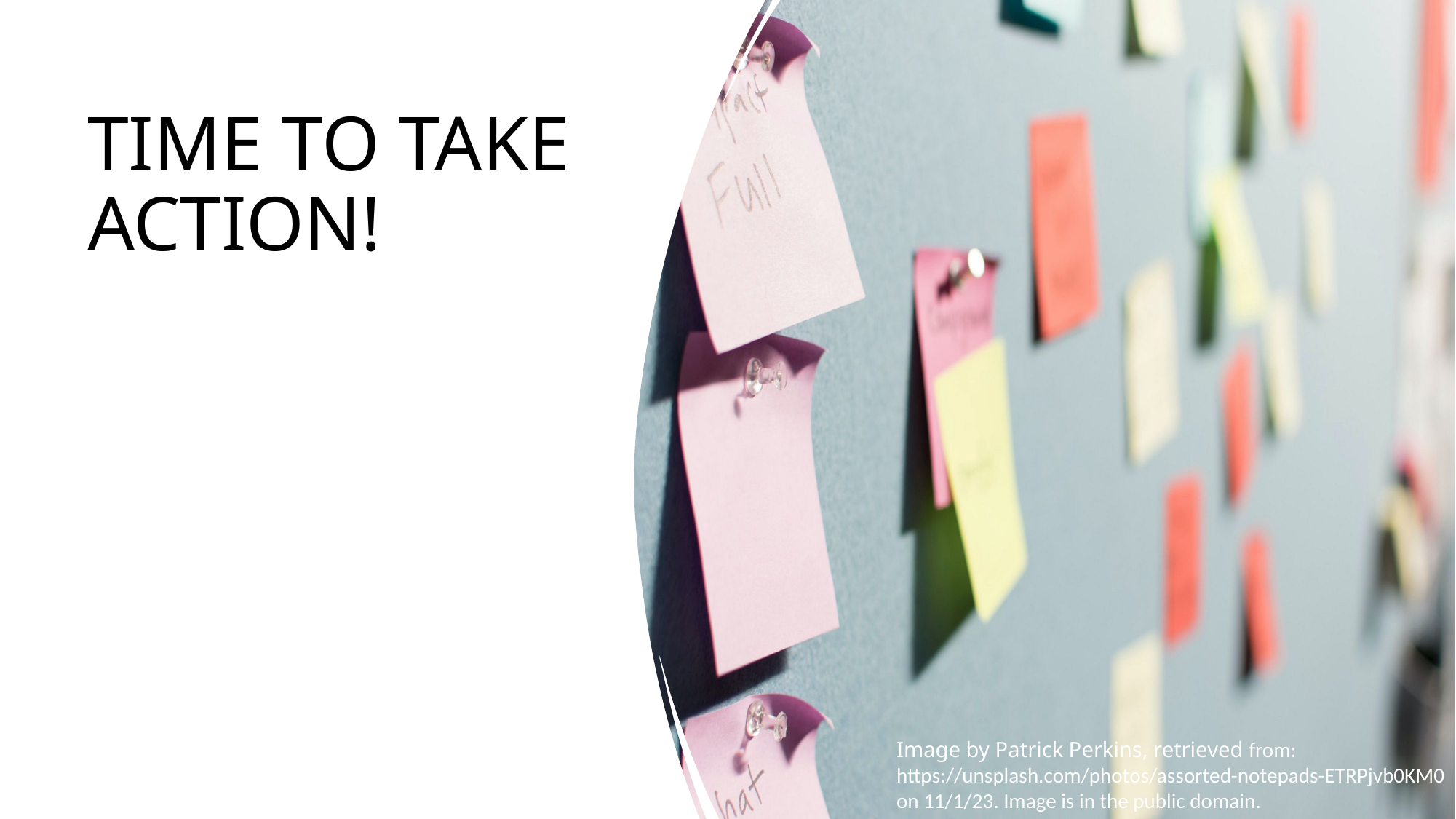

# TIME TO TAKE ACTION!
Image by Patrick Perkins, retrieved from: https://unsplash.com/photos/assorted-notepads-ETRPjvb0KM0 on 11/1/23. Image is in the public domain.

## Slide 34
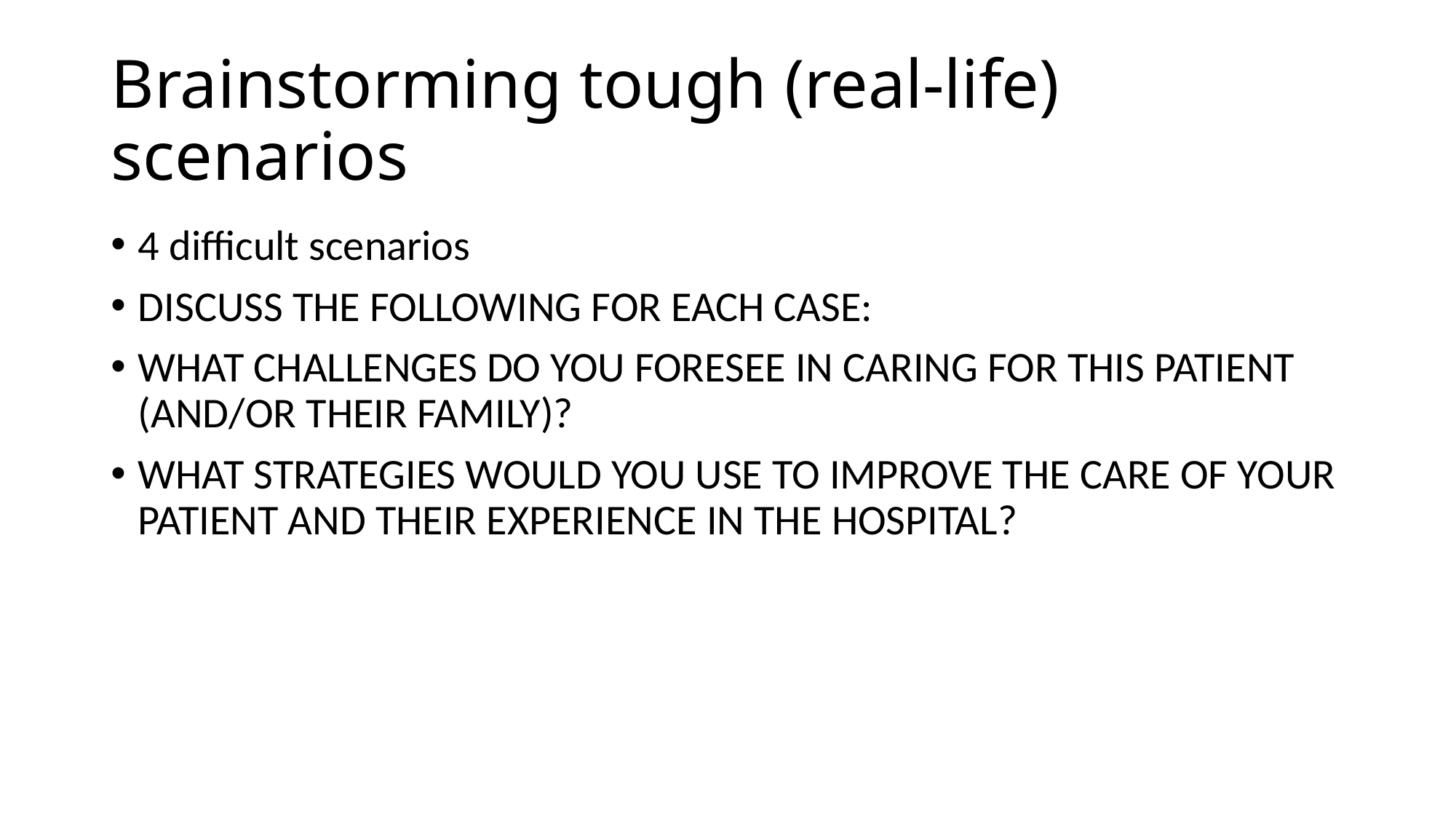

# Brainstorming tough (real-life) scenarios
4 difficult scenarios
DISCUSS THE FOLLOWING FOR EACH CASE:
WHAT CHALLENGES DO YOU FORESEE IN CARING FOR THIS PATIENT (AND/OR THEIR FAMILY)?
WHAT STRATEGIES WOULD YOU USE TO IMPROVE THE CARE OF YOUR PATIENT AND THEIR EXPERIENCE IN THE HOSPITAL?

## Slide 35
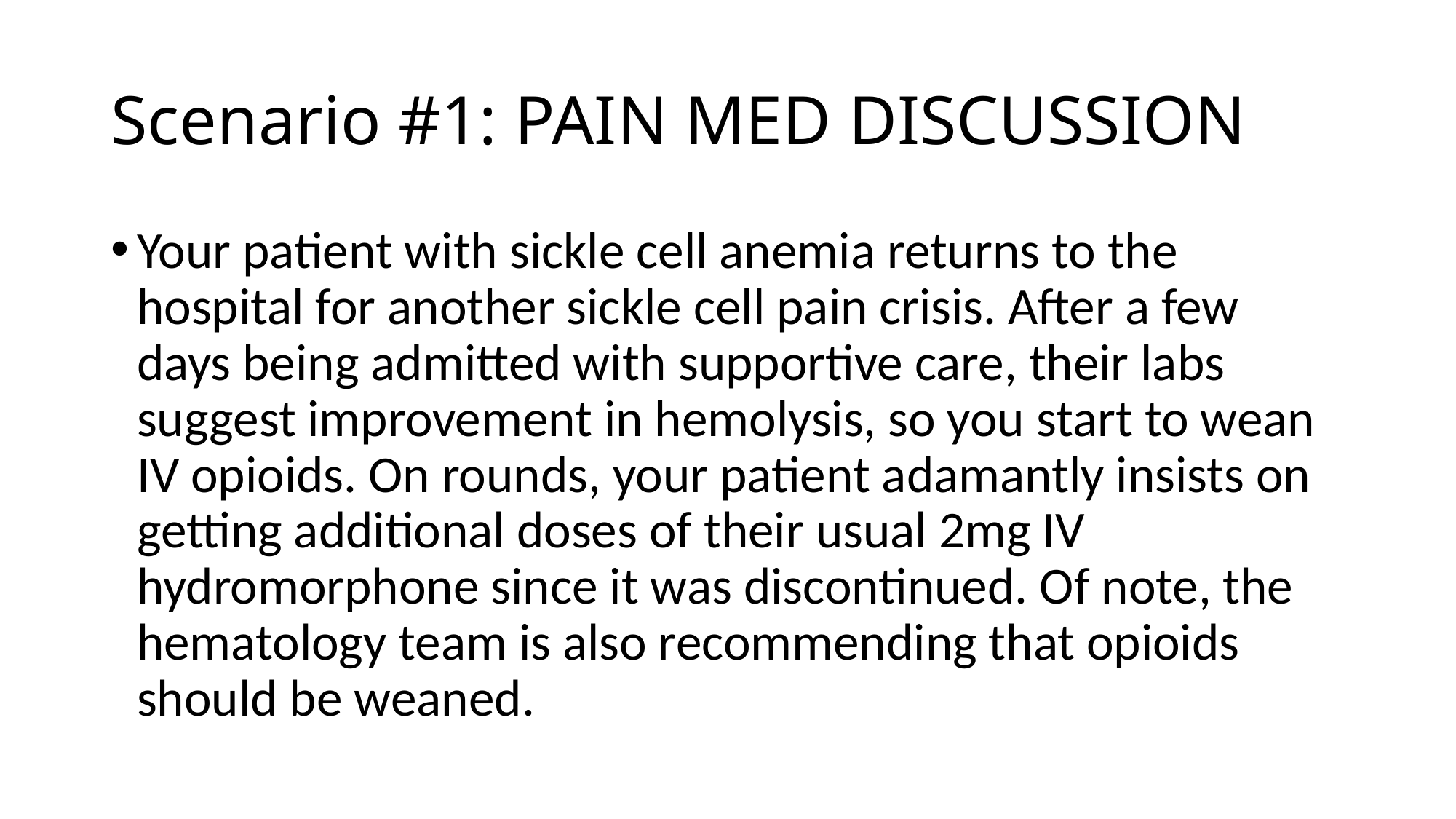

# Scenario #1: PAIN MED DISCUSSION
Your patient with sickle cell anemia returns to the hospital for another sickle cell pain crisis. After a few days being admitted with supportive care, their labs suggest improvement in hemolysis, so you start to wean IV opioids. On rounds, your patient adamantly insists on getting additional doses of their usual 2mg IV hydromorphone since it was discontinued. Of note, the hematology team is also recommending that opioids should be weaned.

## Slide 36
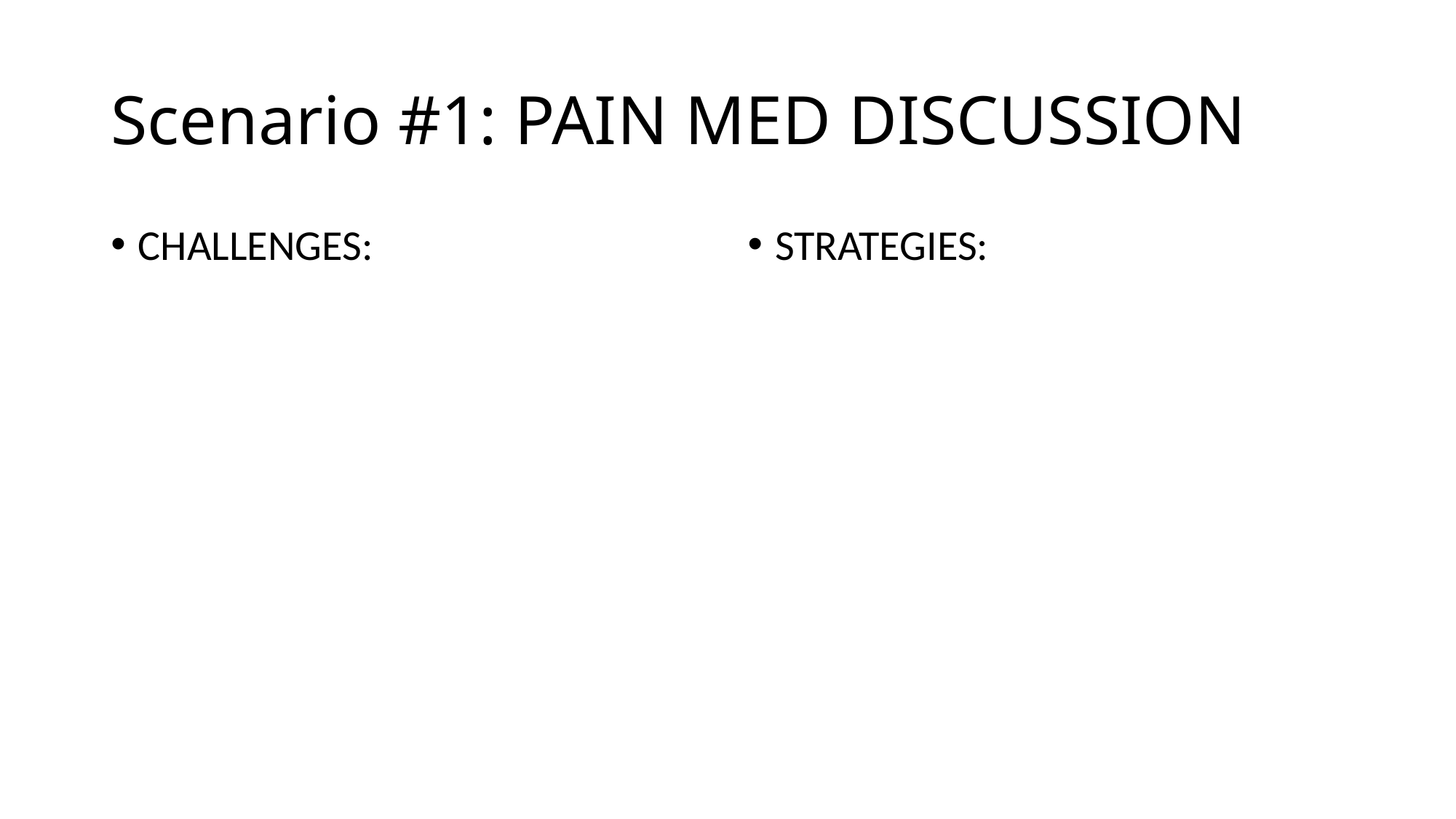

# Scenario #1: PAIN MED DISCUSSION
CHALLENGES:
STRATEGIES:

## Slide 37
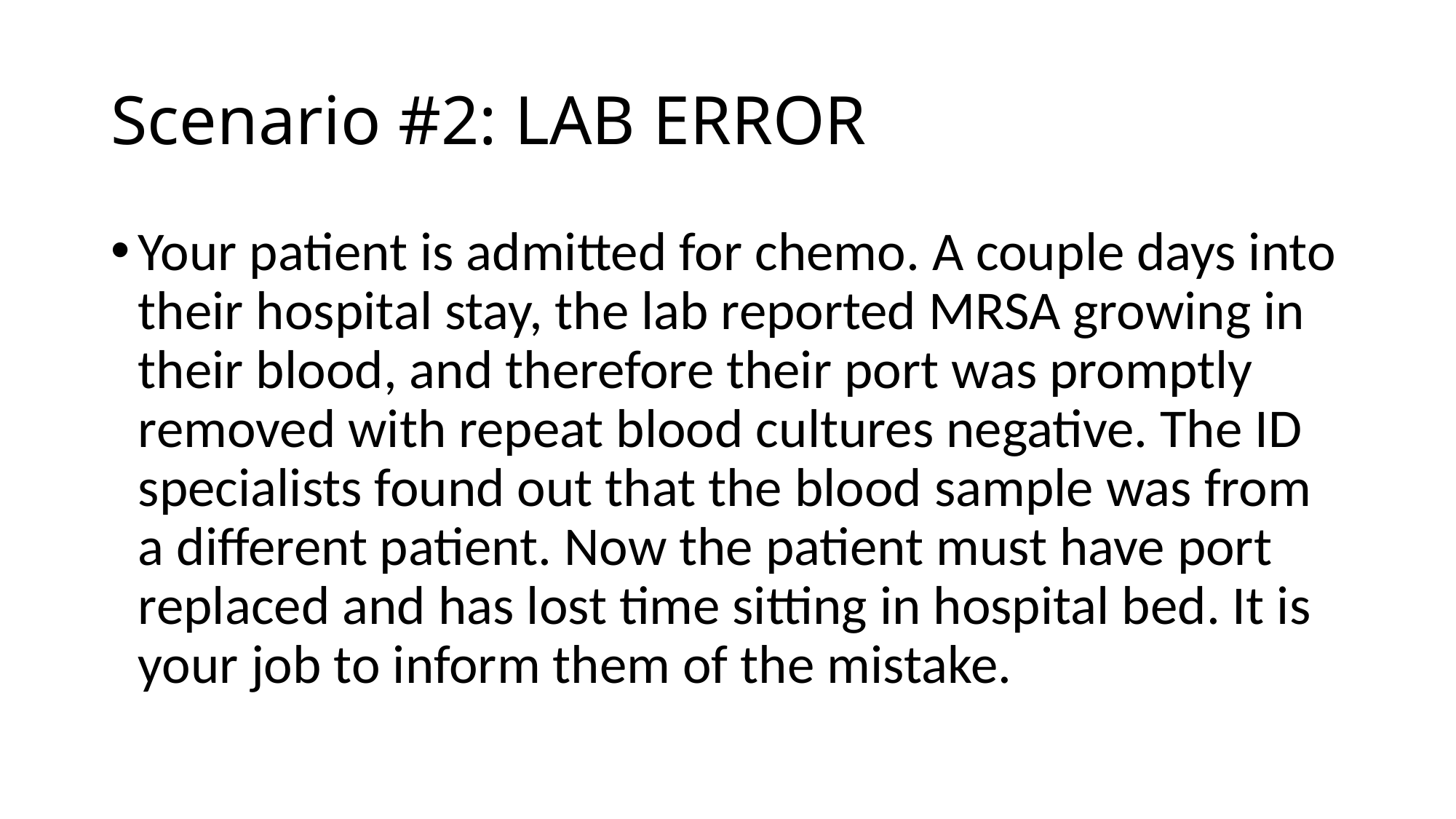

# Scenario #2: LAB ERROR
Your patient is admitted for chemo. A couple days into their hospital stay, the lab reported MRSA growing in their blood, and therefore their port was promptly removed with repeat blood cultures negative. The ID specialists found out that the blood sample was from a different patient. Now the patient must have port replaced and has lost time sitting in hospital bed. It is your job to inform them of the mistake.

## Slide 38
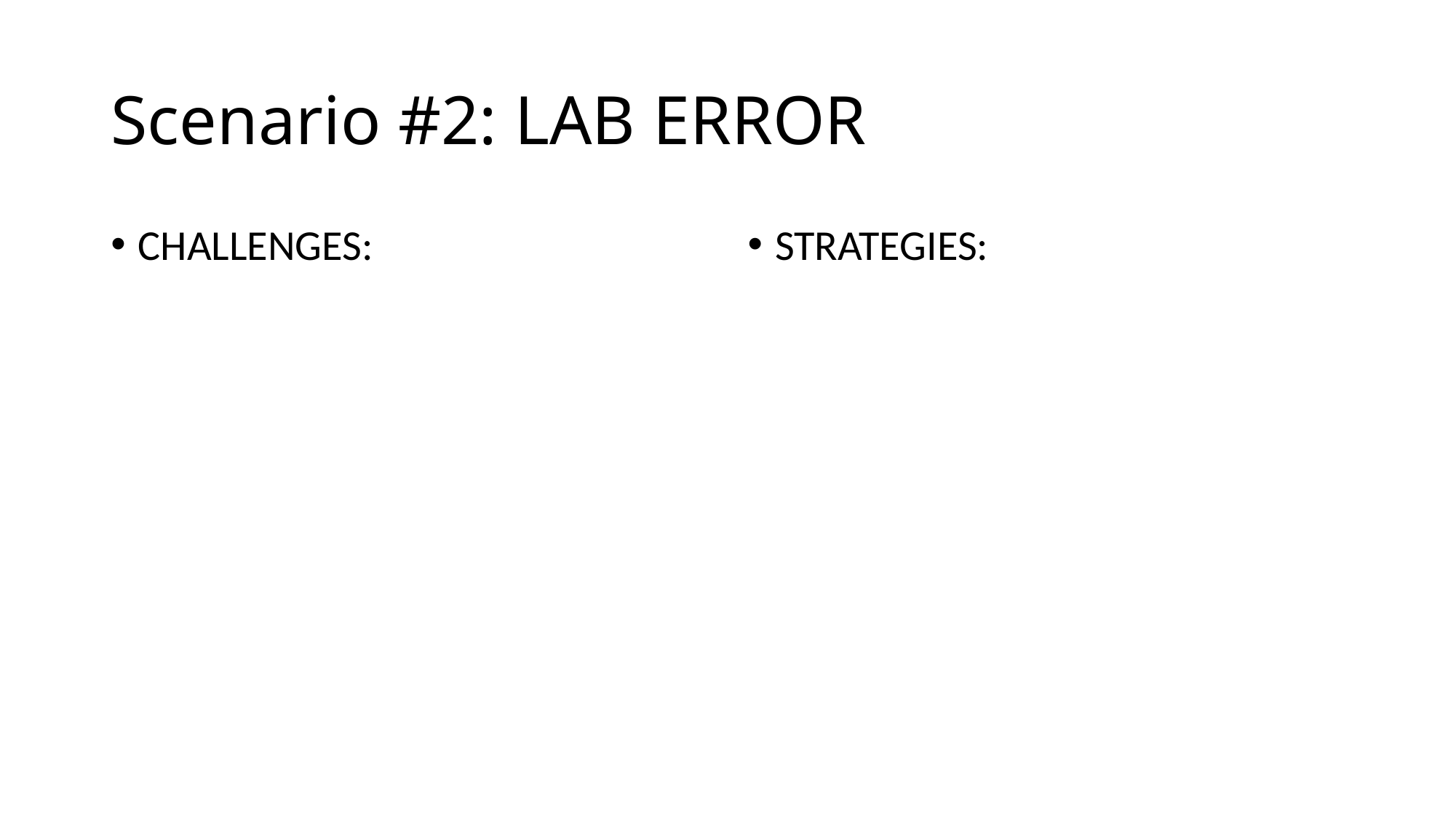

# Scenario #2: LAB ERROR
CHALLENGES:
STRATEGIES:

## Slide 39
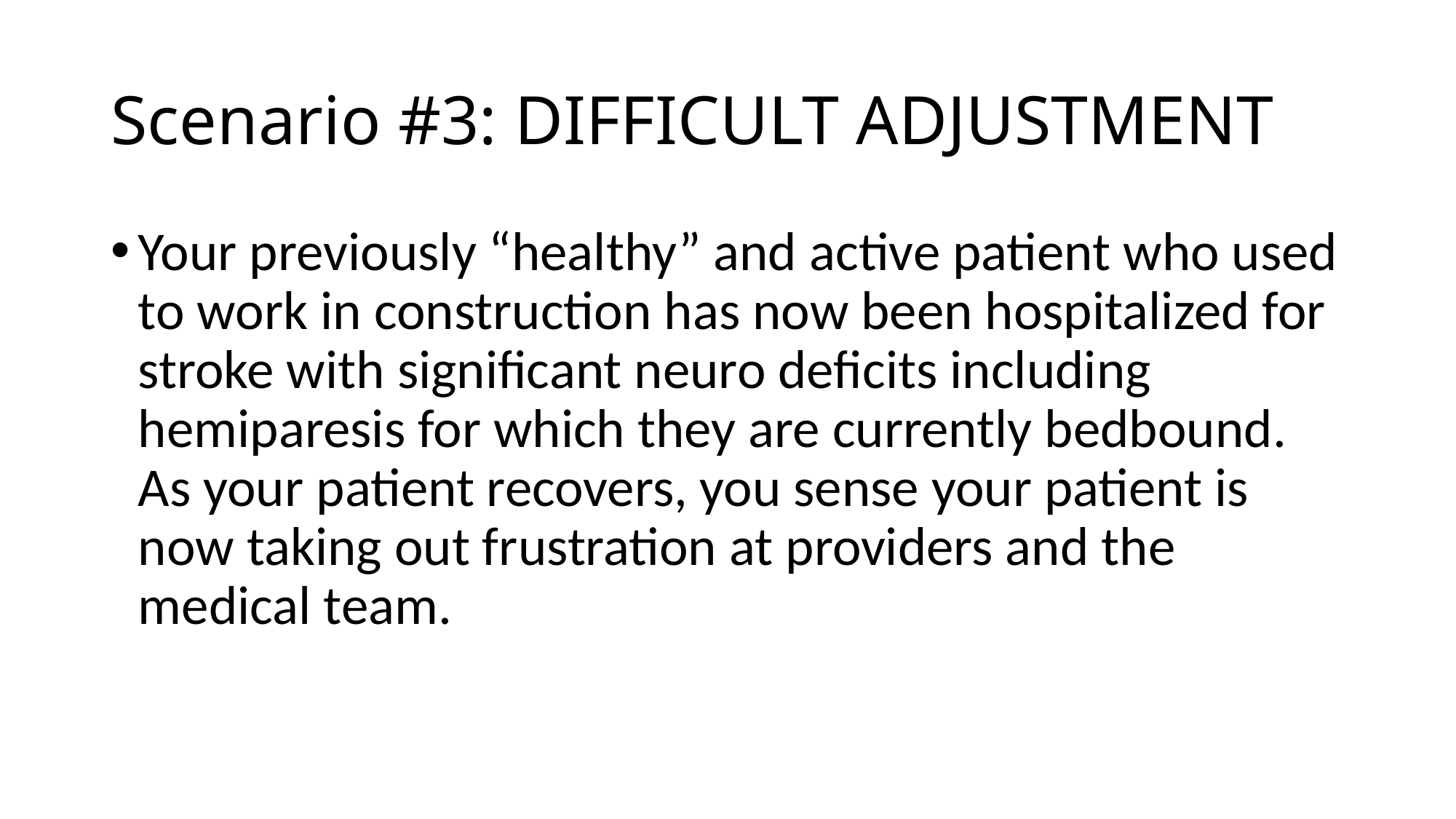

# Scenario #3: DIFFICULT ADJUSTMENT
Your previously “healthy” and active patient who used to work in construction has now been hospitalized for stroke with significant neuro deficits including hemiparesis for which they are currently bedbound. As your patient recovers, you sense your patient is now taking out frustration at providers and the medical team.

## Slide 40
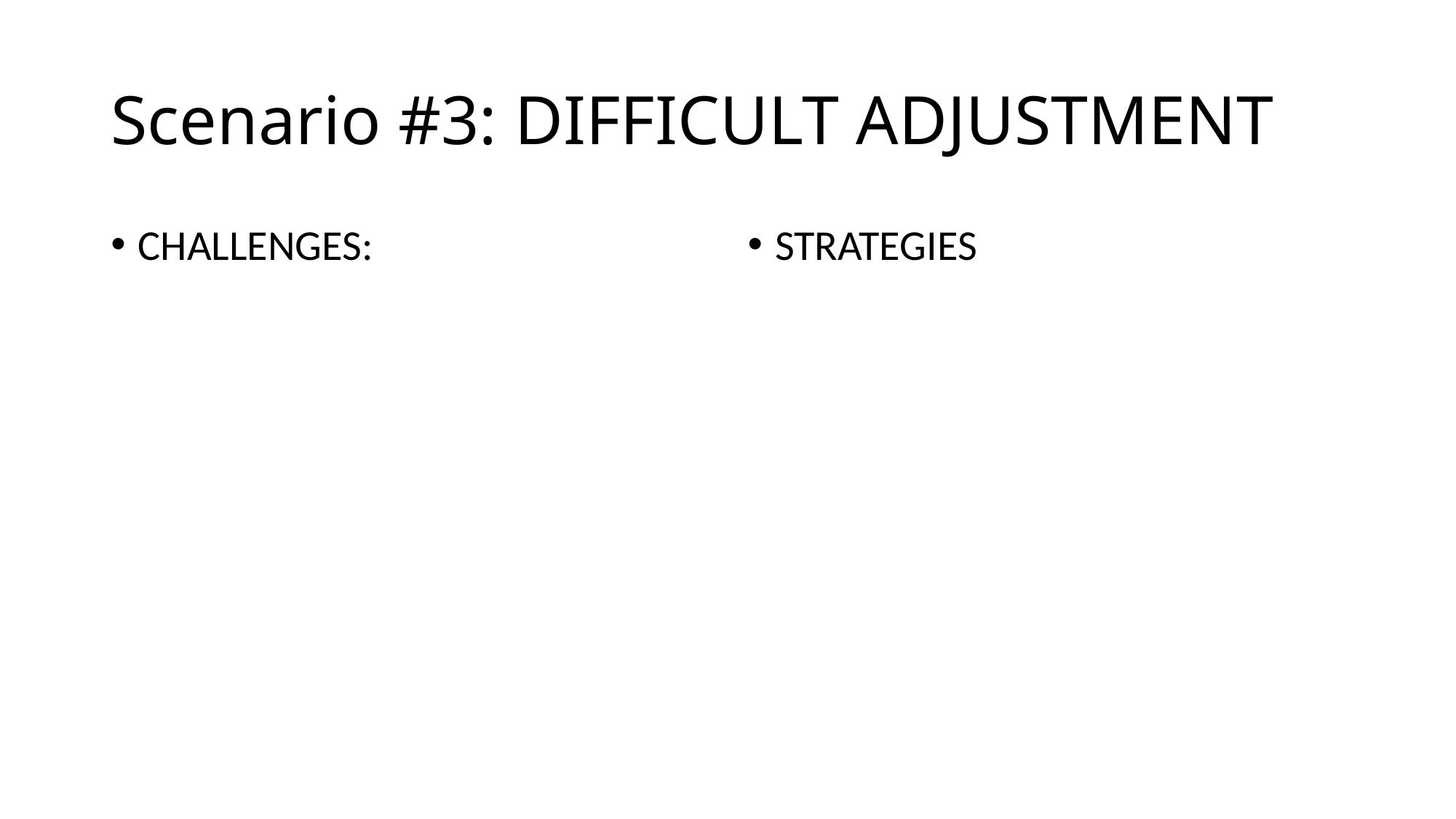

# Scenario #3: DIFFICULT ADJUSTMENT
CHALLENGES:
STRATEGIES

## Slide 41
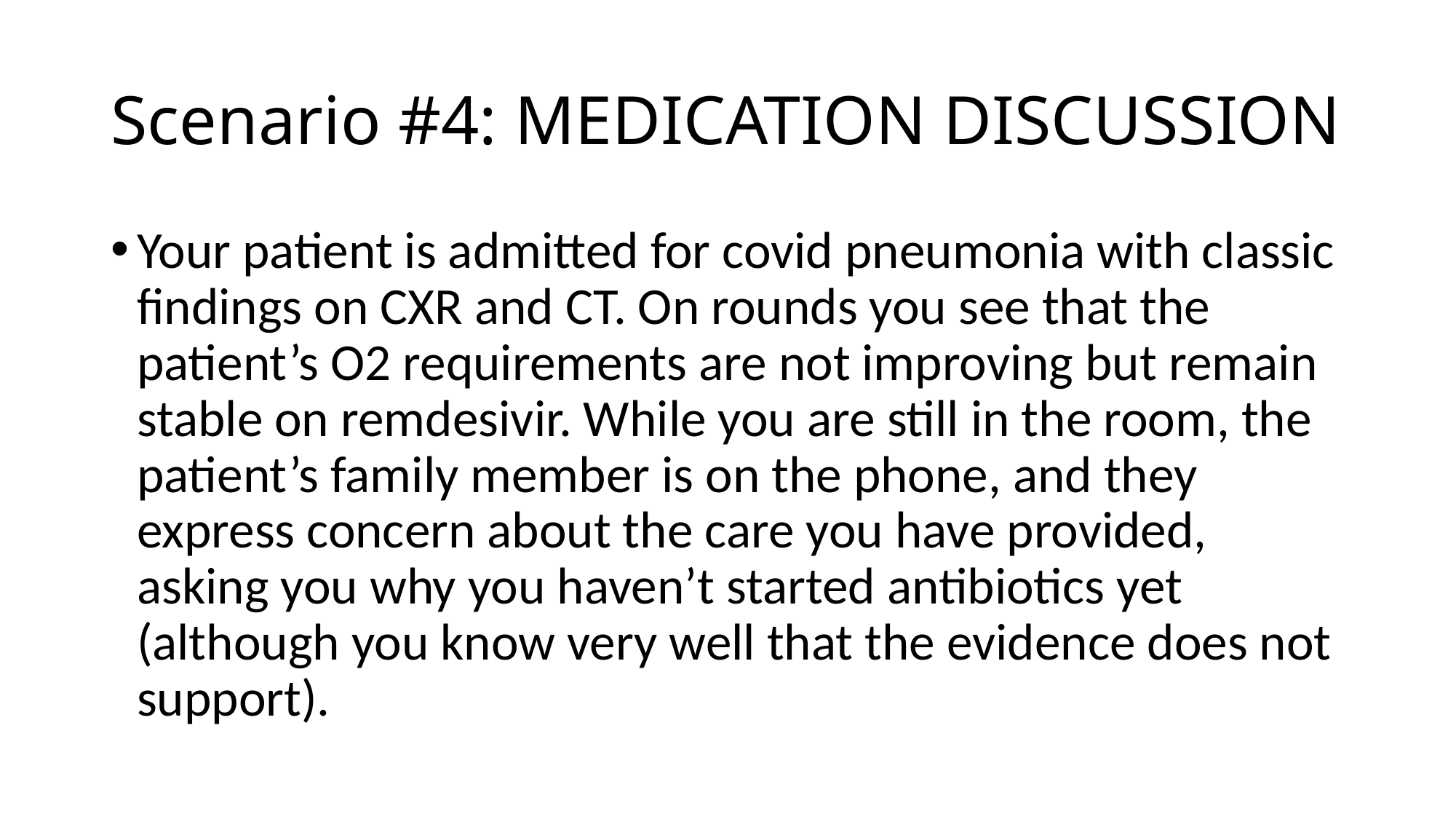

# Scenario #4: MEDICATION DISCUSSION
Your patient is admitted for covid pneumonia with classic findings on CXR and CT. On rounds you see that the patient’s O2 requirements are not improving but remain stable on remdesivir. While you are still in the room, the patient’s family member is on the phone, and they express concern about the care you have provided, asking you why you haven’t started antibiotics yet (although you know very well that the evidence does not support).

## Slide 42
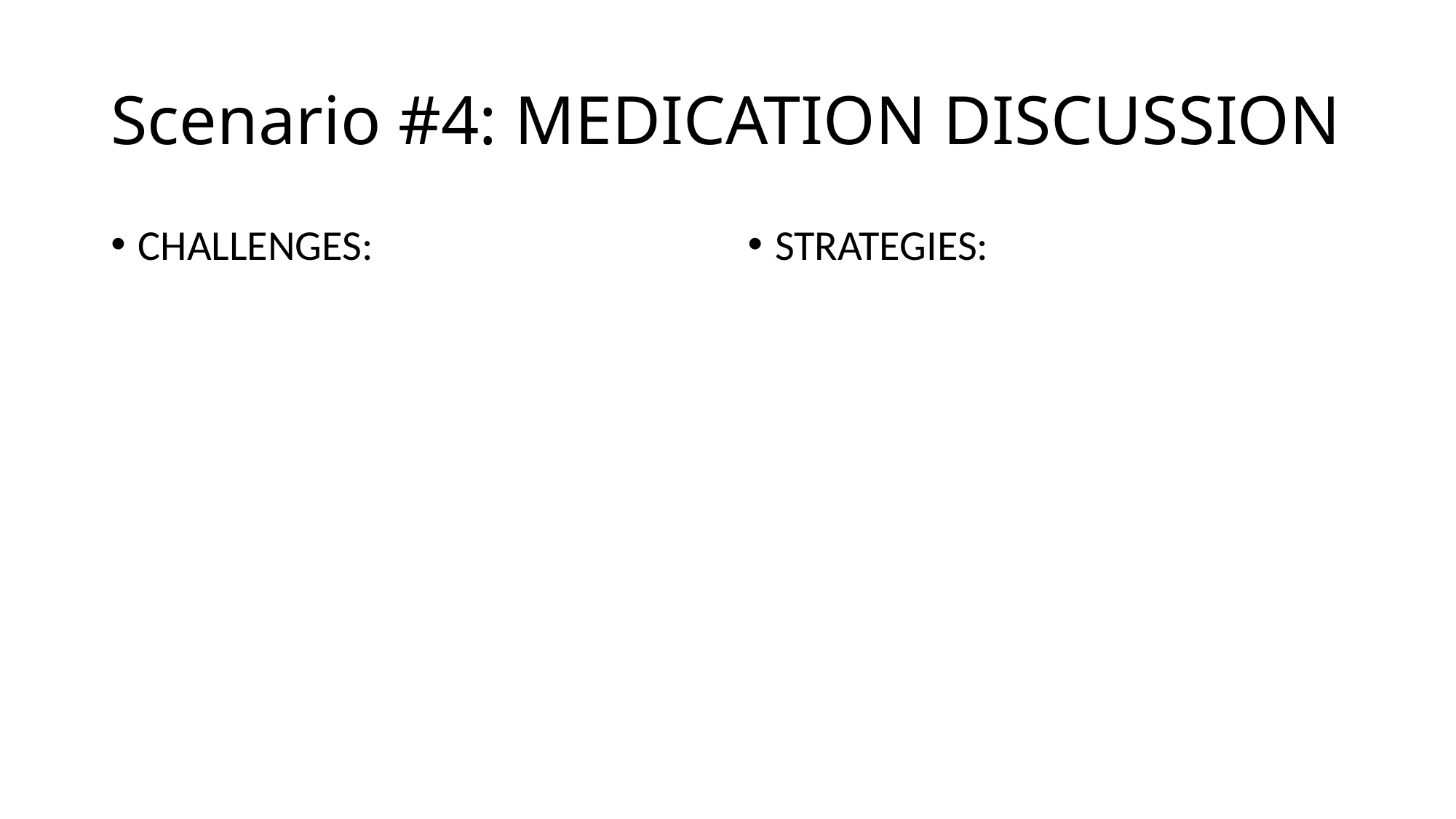

# Scenario #4: MEDICATION DISCUSSION
CHALLENGES:
STRATEGIES:

## Slide 43
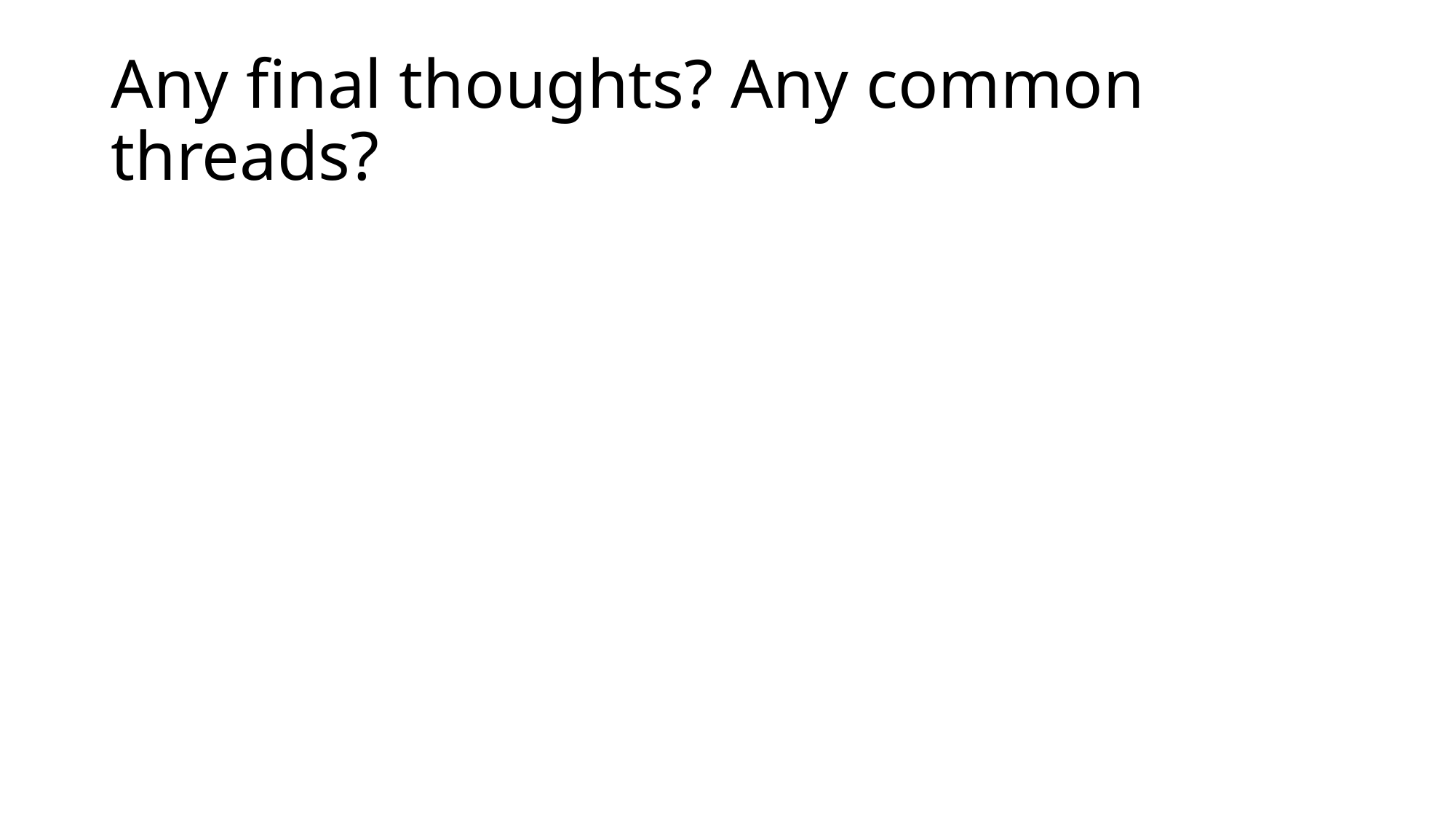

# Any final thoughts? Any common threads?

## Slide 44
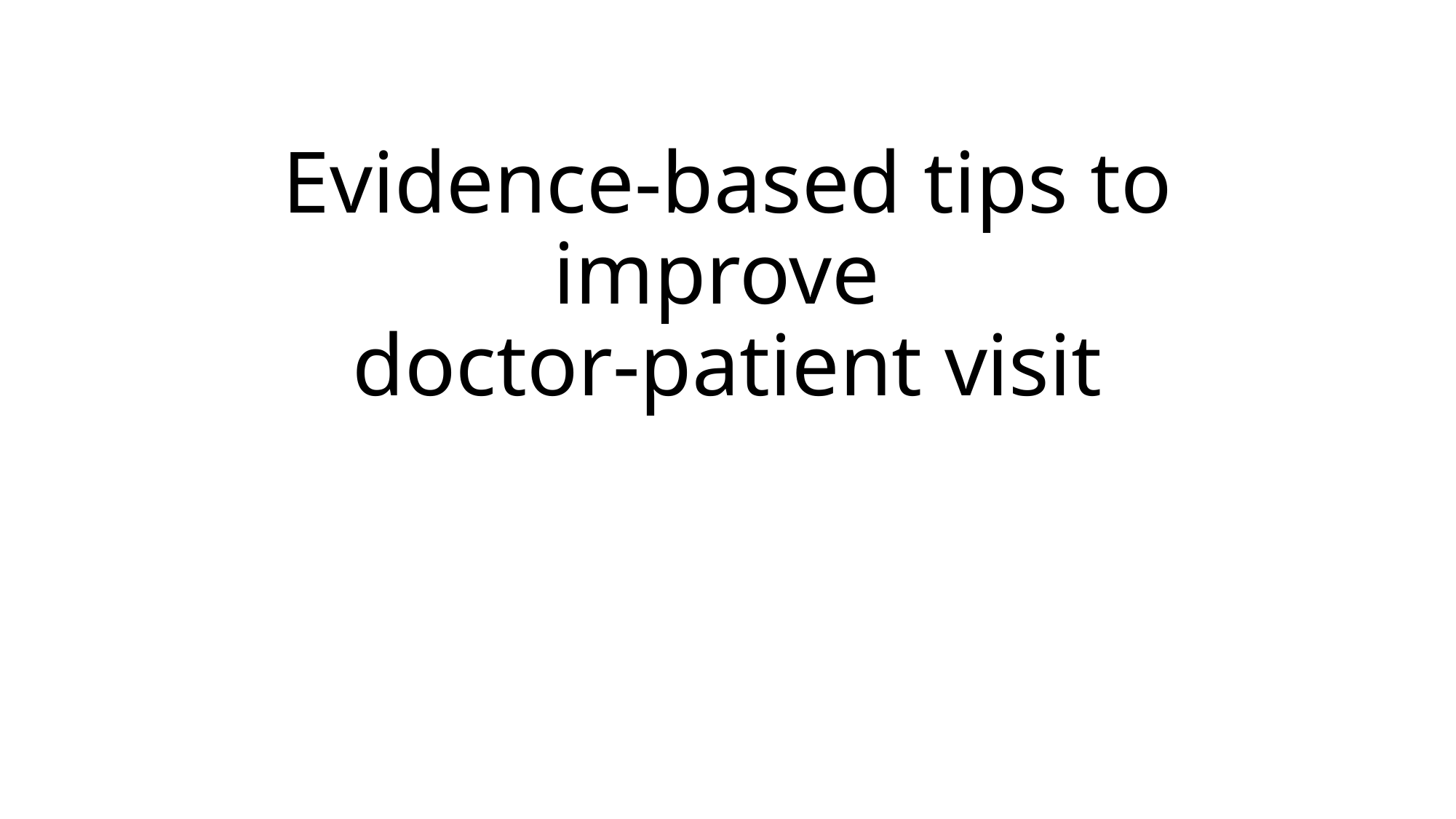

# Evidence-based tips to improve doctor-patient visit

## Slide 45
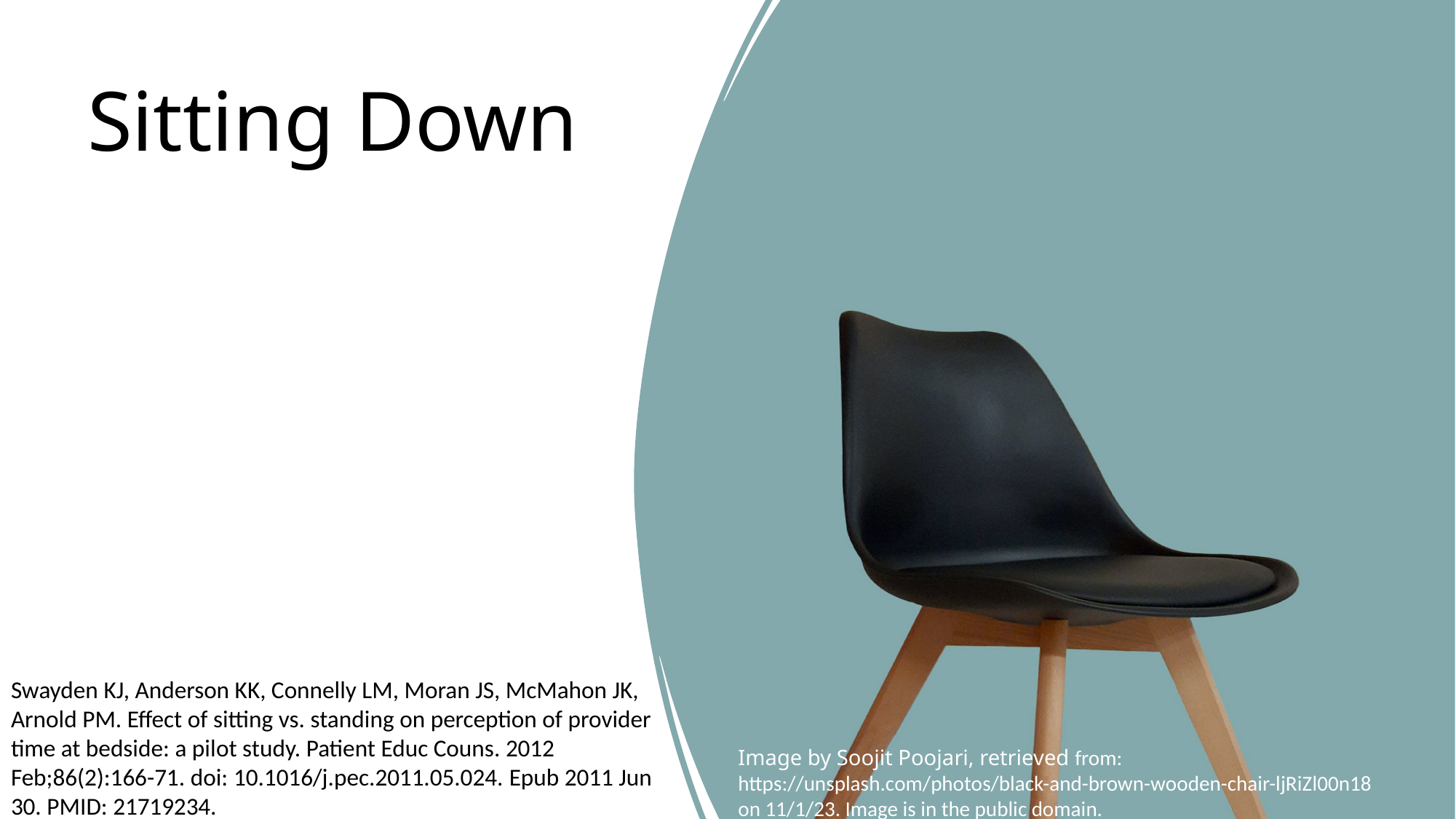

# Sitting Down
Swayden KJ, Anderson KK, Connelly LM, Moran JS, McMahon JK, Arnold PM. Effect of sitting vs. standing on perception of provider time at bedside: a pilot study. Patient Educ Couns. 2012 Feb;86(2):166-71. doi: 10.1016/j.pec.2011.05.024. Epub 2011 Jun 30. PMID: 21719234.
Image by Soojit Poojari, retrieved from: https://unsplash.com/photos/black-and-brown-wooden-chair-ljRiZl00n18 on 11/1/23. Image is in the public domain.

## Slide 46
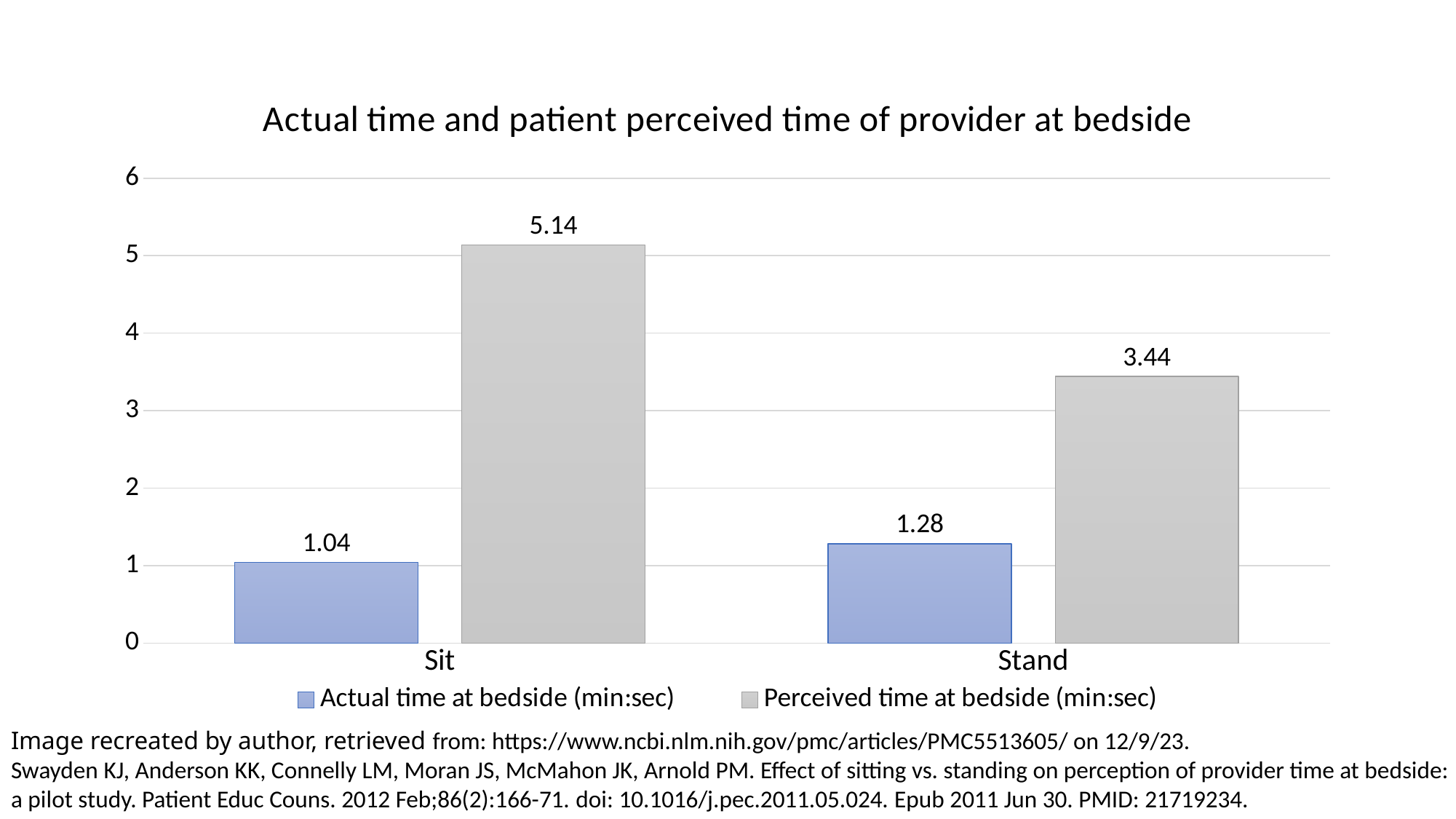

### Chart: Actual time and patient perceived time of provider at bedside
| Category | Actual time at bedside (min:sec) | Perceived time at bedside (min:sec) |
|---|---|---|
| Sit | 1.04 | 5.14 |
| Stand | 1.28 | 3.44 |Image recreated by author, retrieved from: https://www.ncbi.nlm.nih.gov/pmc/articles/PMC5513605/ on 12/9/23.
Swayden KJ, Anderson KK, Connelly LM, Moran JS, McMahon JK, Arnold PM. Effect of sitting vs. standing on perception of provider time at bedside: a pilot study. Patient Educ Couns. 2012 Feb;86(2):166-71. doi: 10.1016/j.pec.2011.05.024. Epub 2011 Jun 30. PMID: 21719234.

## Slide 47
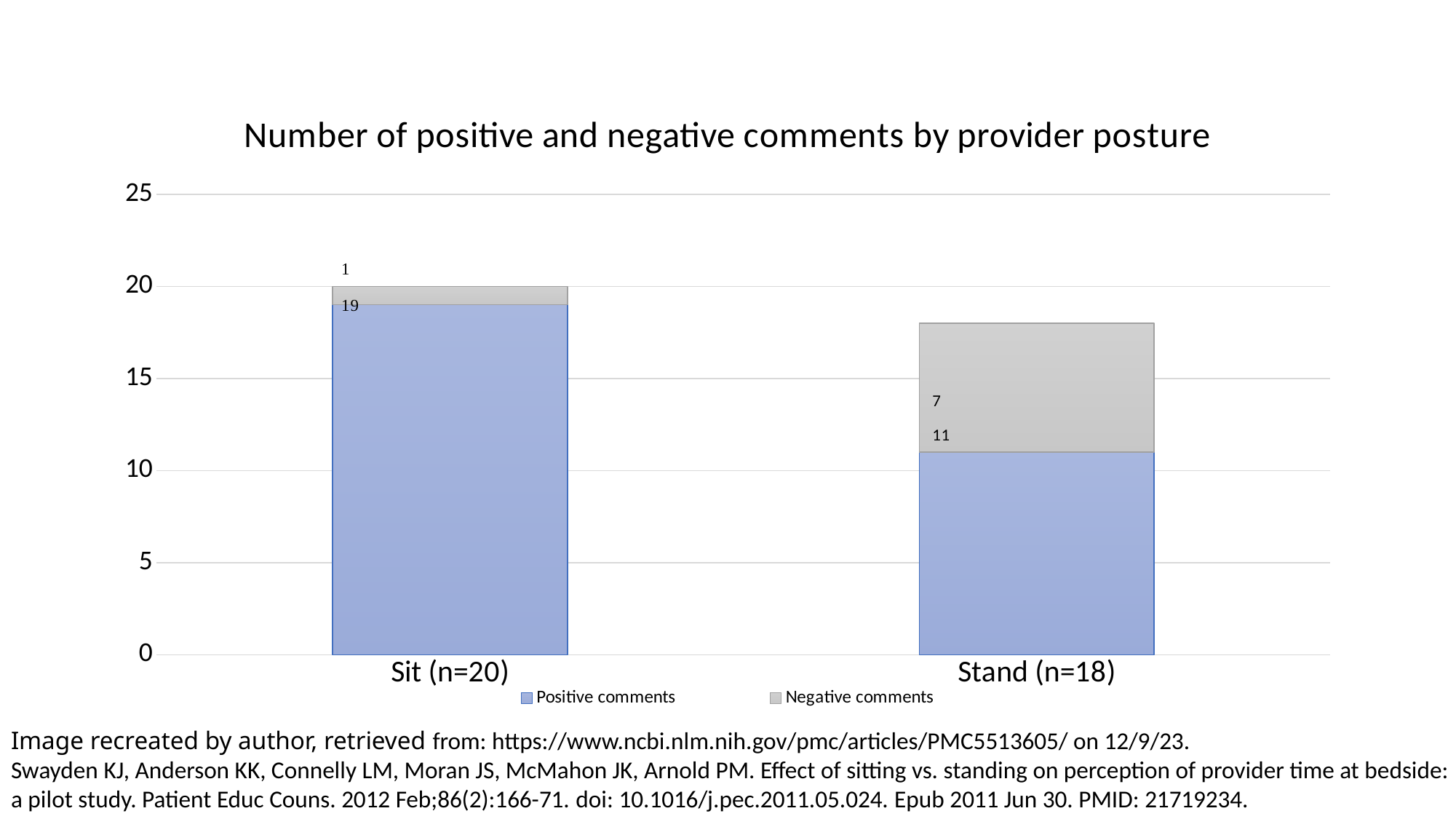

### Chart: Number of positive and negative comments by provider posture
| Category | Positive comments | Negative comments |
|---|---|---|
| Sit (n=20) | 19.0 | 1.0 |
| Stand (n=18) | 11.0 | 7.0 |Image recreated by author, retrieved from: https://www.ncbi.nlm.nih.gov/pmc/articles/PMC5513605/ on 12/9/23.
Swayden KJ, Anderson KK, Connelly LM, Moran JS, McMahon JK, Arnold PM. Effect of sitting vs. standing on perception of provider time at bedside: a pilot study. Patient Educ Couns. 2012 Feb;86(2):166-71. doi: 10.1016/j.pec.2011.05.024. Epub 2011 Jun 30. PMID: 21719234.

## Slide 48
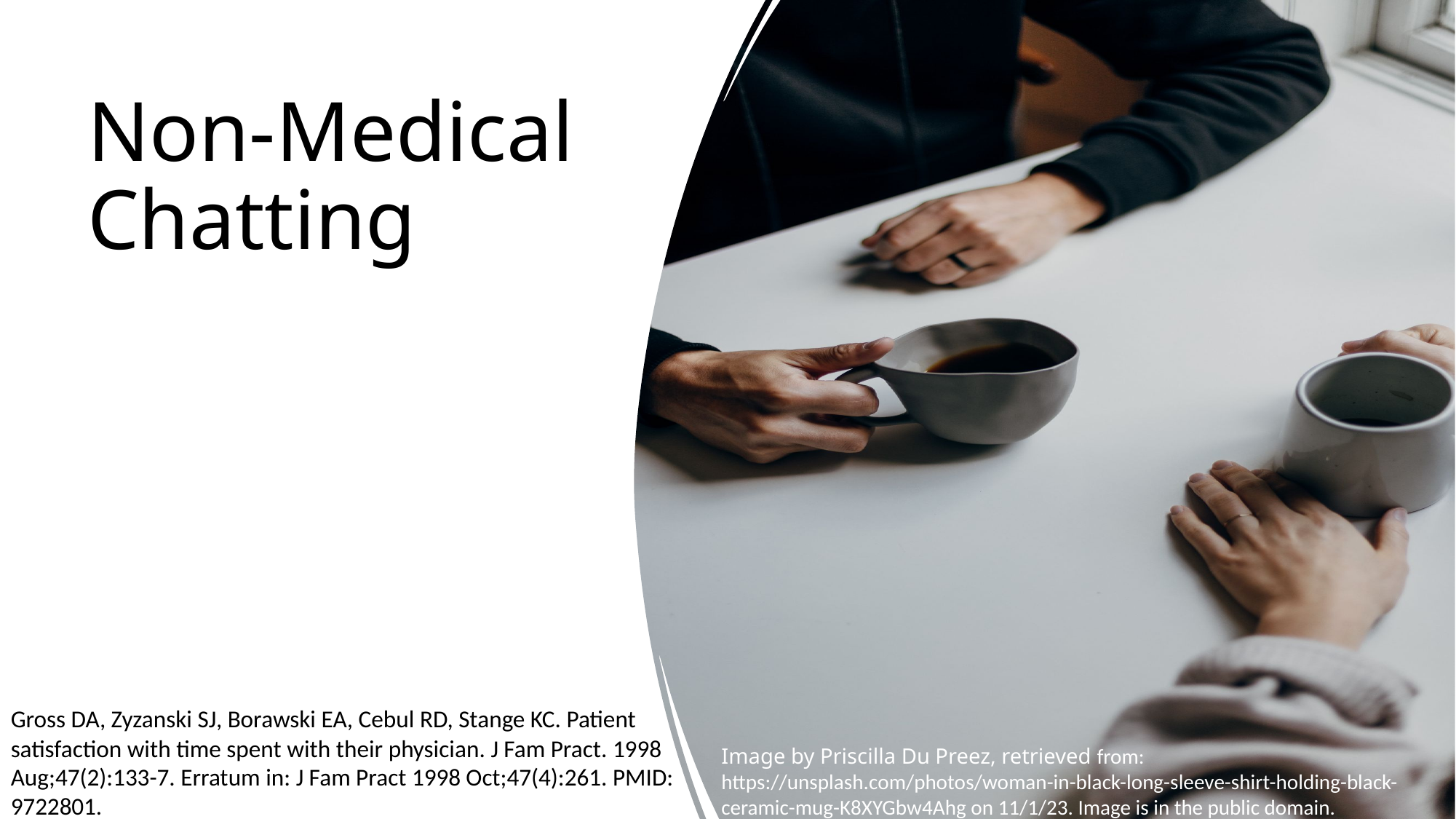

Non-Medical Chatting
Gross DA, Zyzanski SJ, Borawski EA, Cebul RD, Stange KC. Patient satisfaction with time spent with their physician. J Fam Pract. 1998 Aug;47(2):133-7. Erratum in: J Fam Pract 1998 Oct;47(4):261. PMID: 9722801.
Image by Priscilla Du Preez, retrieved from: https://unsplash.com/photos/woman-in-black-long-sleeve-shirt-holding-black-ceramic-mug-K8XYGbw4Ahg on 11/1/23. Image is in the public domain.

## Slide 49
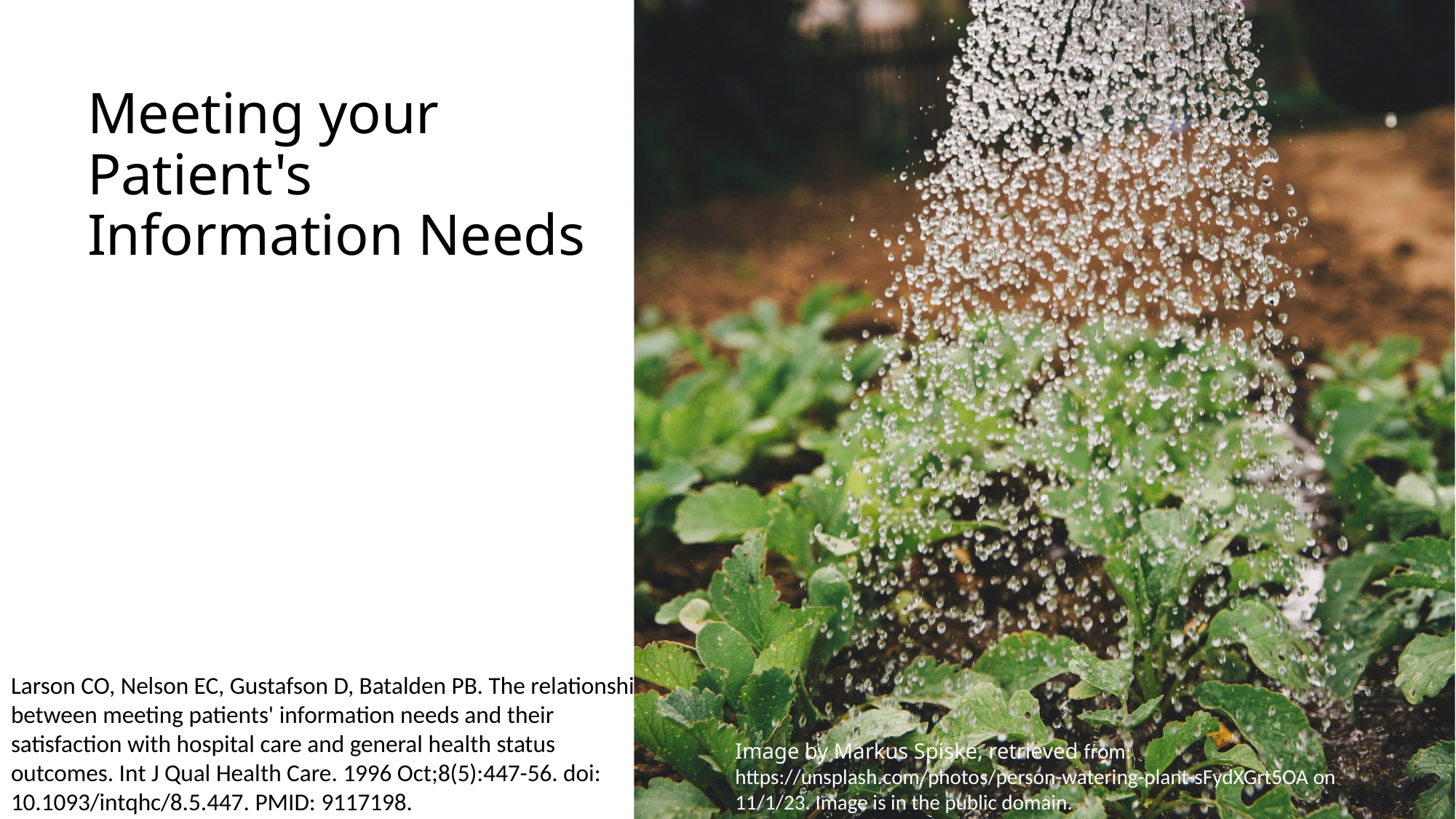

# Meeting your Patient's Information Needs
Larson CO, Nelson EC, Gustafson D, Batalden PB. The relationship between meeting patients' information needs and their satisfaction with hospital care and general health status outcomes. Int J Qual Health Care. 1996 Oct;8(5):447-56. doi: 10.1093/intqhc/8.5.447. PMID: 9117198.
Image by Markus Spiske, retrieved from: https://unsplash.com/photos/person-watering-plant-sFydXGrt5OA on 11/1/23. Image is in the public domain.

## Slide 50
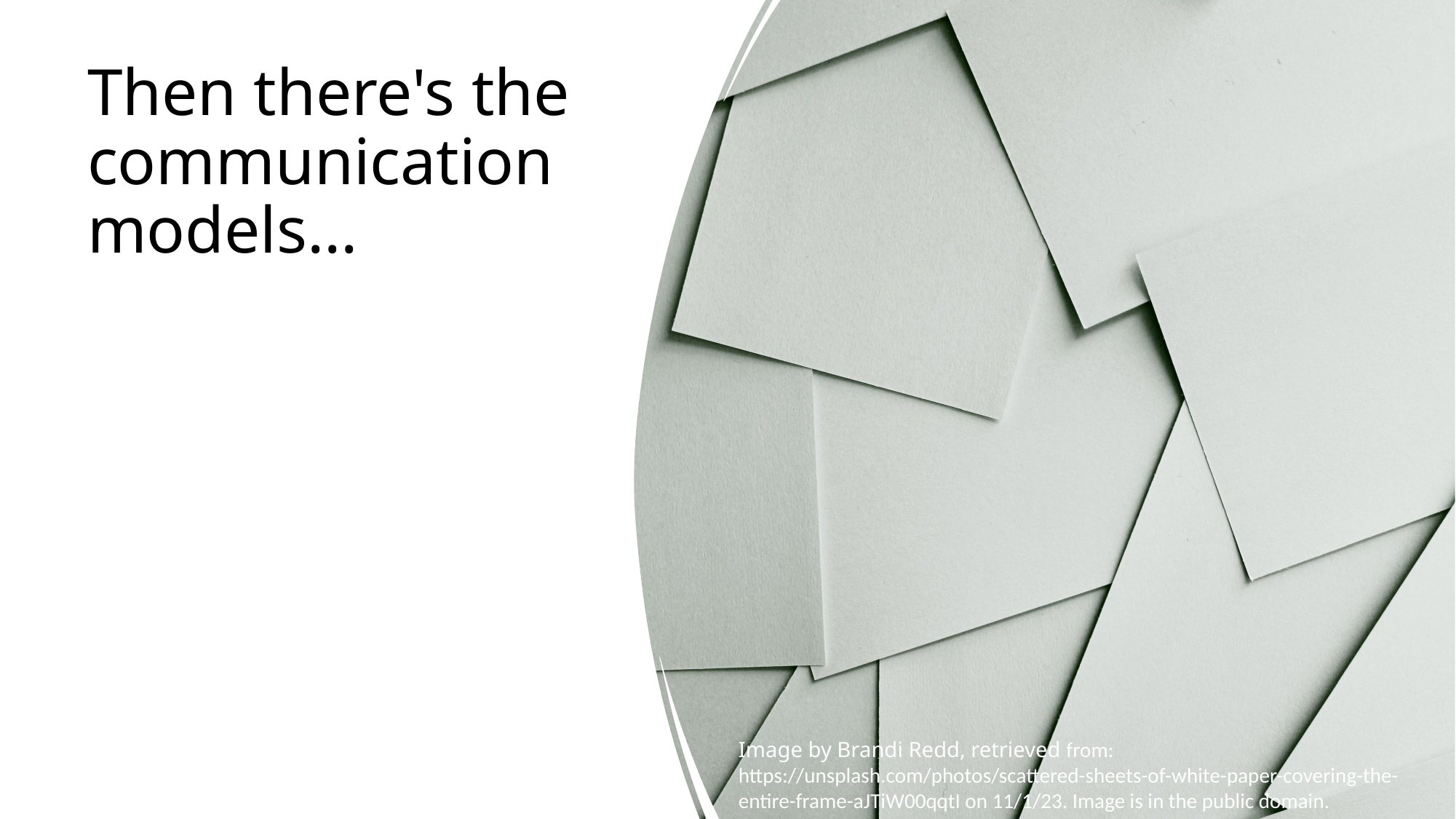

# Then there's the communication models...
Image by Brandi Redd, retrieved from: https://unsplash.com/photos/scattered-sheets-of-white-paper-covering-the-entire-frame-aJTiW00qqtI on 11/1/23. Image is in the public domain.

## Slide 51
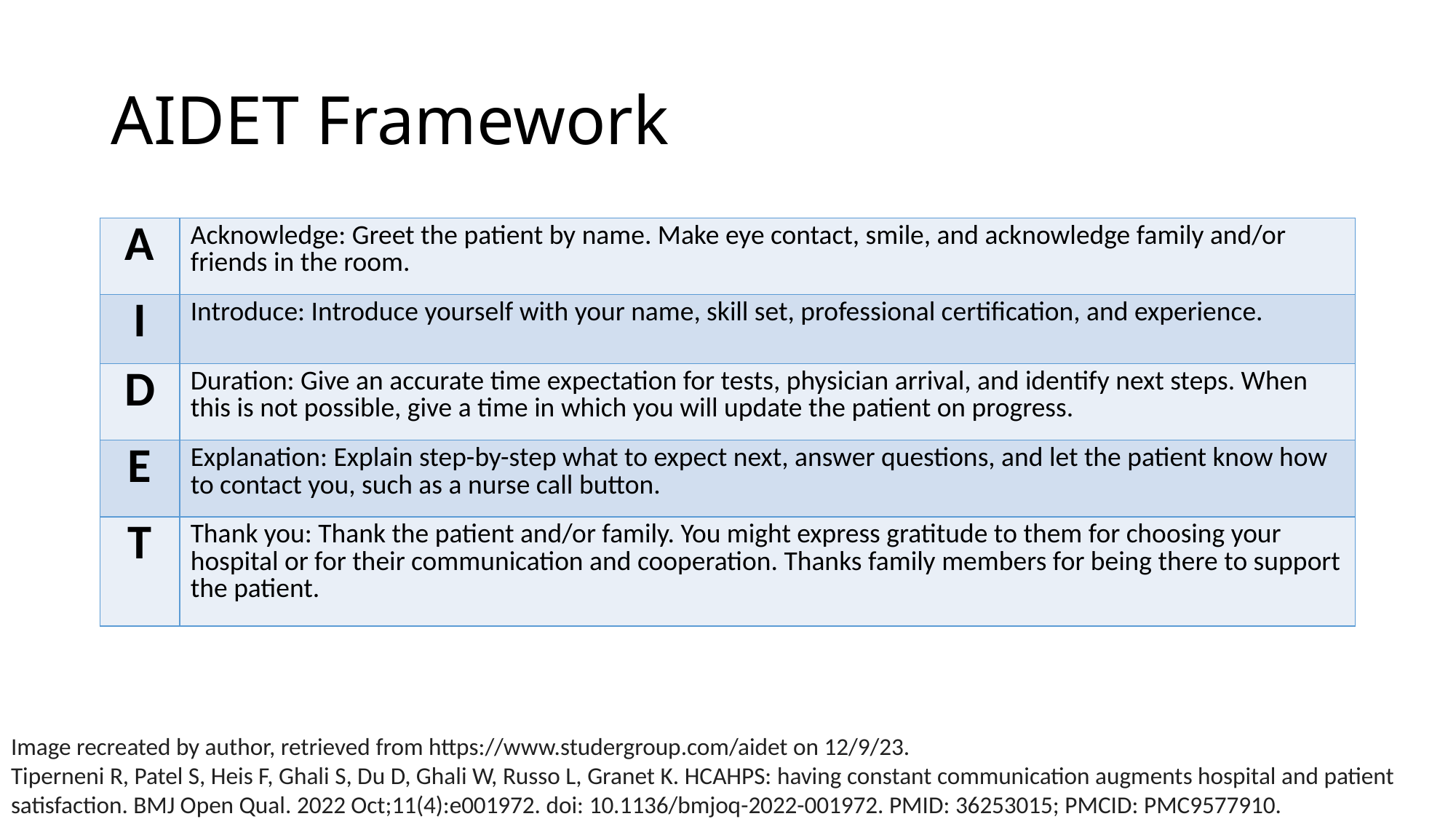

# AIDET Framework
| A | Acknowledge: Greet the patient by name. Make eye contact, smile, and acknowledge family and/or friends in the room. |
| --- | --- |
| I | Introduce: Introduce yourself with your name, skill set, professional certification, and experience. |
| D | Duration: Give an accurate time expectation for tests, physician arrival, and identify next steps. When this is not possible, give a time in which you will update the patient on progress. |
| E | Explanation: Explain step-by-step what to expect next, answer questions, and let the patient know how to contact you, such as a nurse call button. |
| T | Thank you: Thank the patient and/or family. You might express gratitude to them for choosing your hospital or for their communication and cooperation. Thanks family members for being there to support the patient. |
Image recreated by author, retrieved from https://www.studergroup.com/aidet on 12/9/23.
Tiperneni R, Patel S, Heis F, Ghali S, Du D, Ghali W, Russo L, Granet K. HCAHPS: having constant communication augments hospital and patient satisfaction. BMJ Open Qual. 2022 Oct;11(4):e001972. doi: 10.1136/bmjoq-2022-001972. PMID: 36253015; PMCID: PMC9577910.

## Slide 52
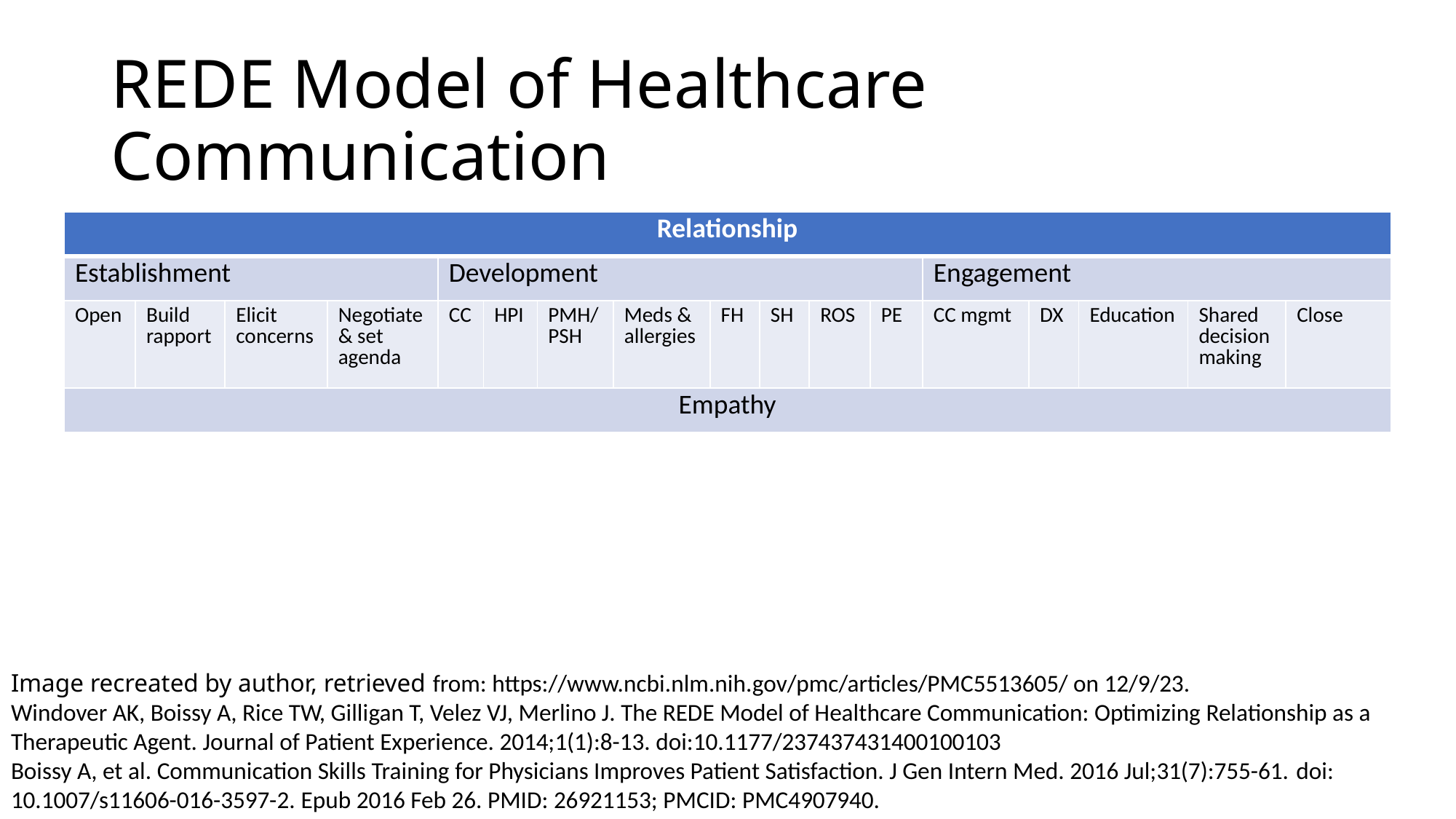

# REDE Model of Healthcare Communication
| Relationship | | | | | | | | | | | | | | | | |
| --- | --- | --- | --- | --- | --- | --- | --- | --- | --- | --- | --- | --- | --- | --- | --- | --- |
| Establishment | | | | Development | | | | | | | | Engagement | | | | |
| Open | Build rapport | Elicit concerns | Negotiate & set agenda | CC | HPI | PMH/ PSH | Meds & allergies | FH | SH | ROS | PE | CC mgmt | DX | Education | Shared decision making | Close |
| Empathy | | | | | | | | | | | | | | | | |
Image recreated by author, retrieved from: https://www.ncbi.nlm.nih.gov/pmc/articles/PMC5513605/ on 12/9/23.
Windover AK, Boissy A, Rice TW, Gilligan T, Velez VJ, Merlino J. The REDE Model of Healthcare Communication: Optimizing Relationship as a Therapeutic Agent. Journal of Patient Experience. 2014;1(1):8-13. doi:10.1177/237437431400100103
Boissy A, et al. Communication Skills Training for Physicians Improves Patient Satisfaction. J Gen Intern Med. 2016 Jul;31(7):755-61. doi: 10.1007/s11606-016-3597-2. Epub 2016 Feb 26. PMID: 26921153; PMCID: PMC4907940.

## Slide 53
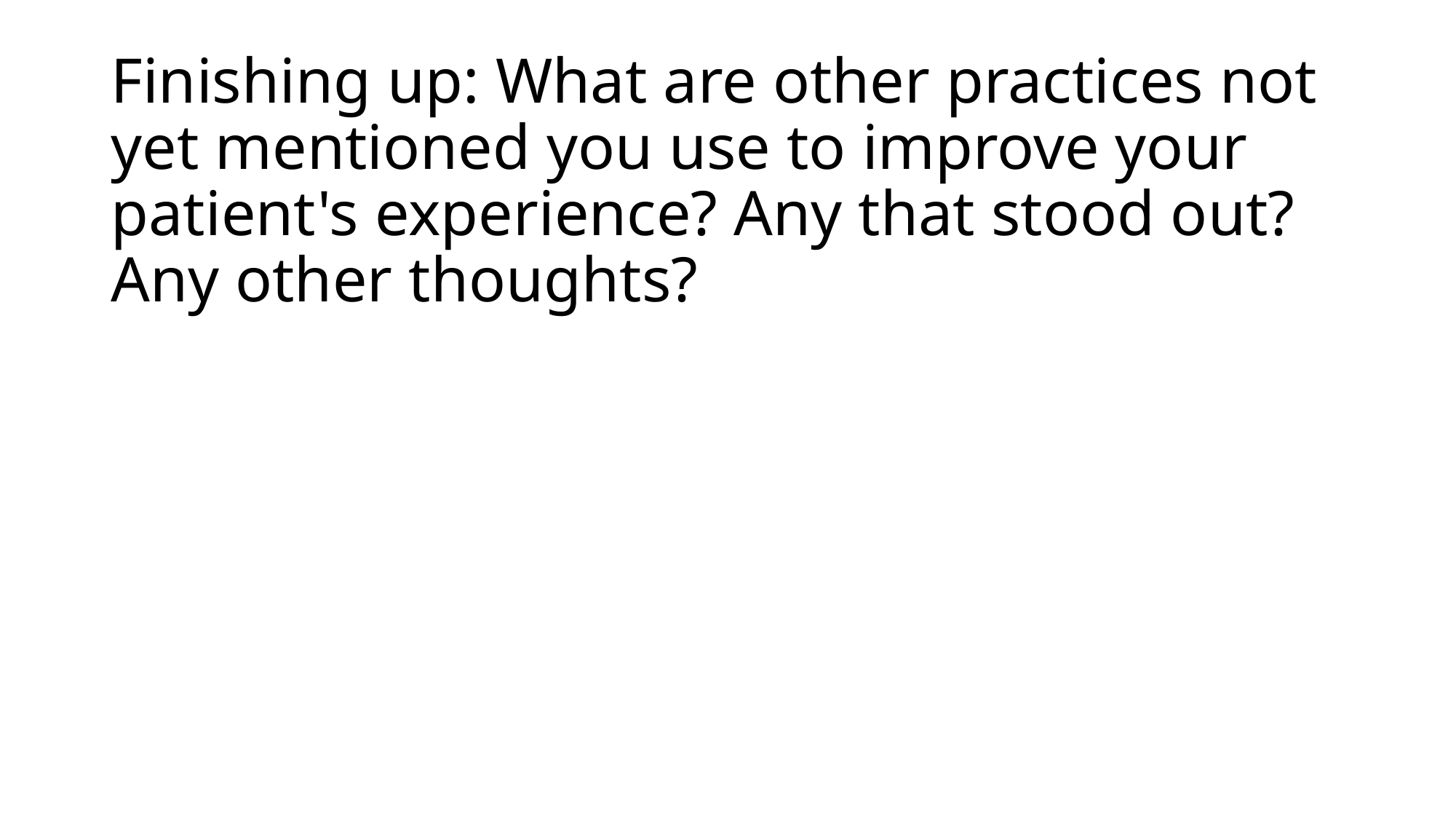

# Finishing up: What are other practices not yet mentioned you use to improve your patient's experience? Any that stood out? Any other thoughts?

## Slide 54
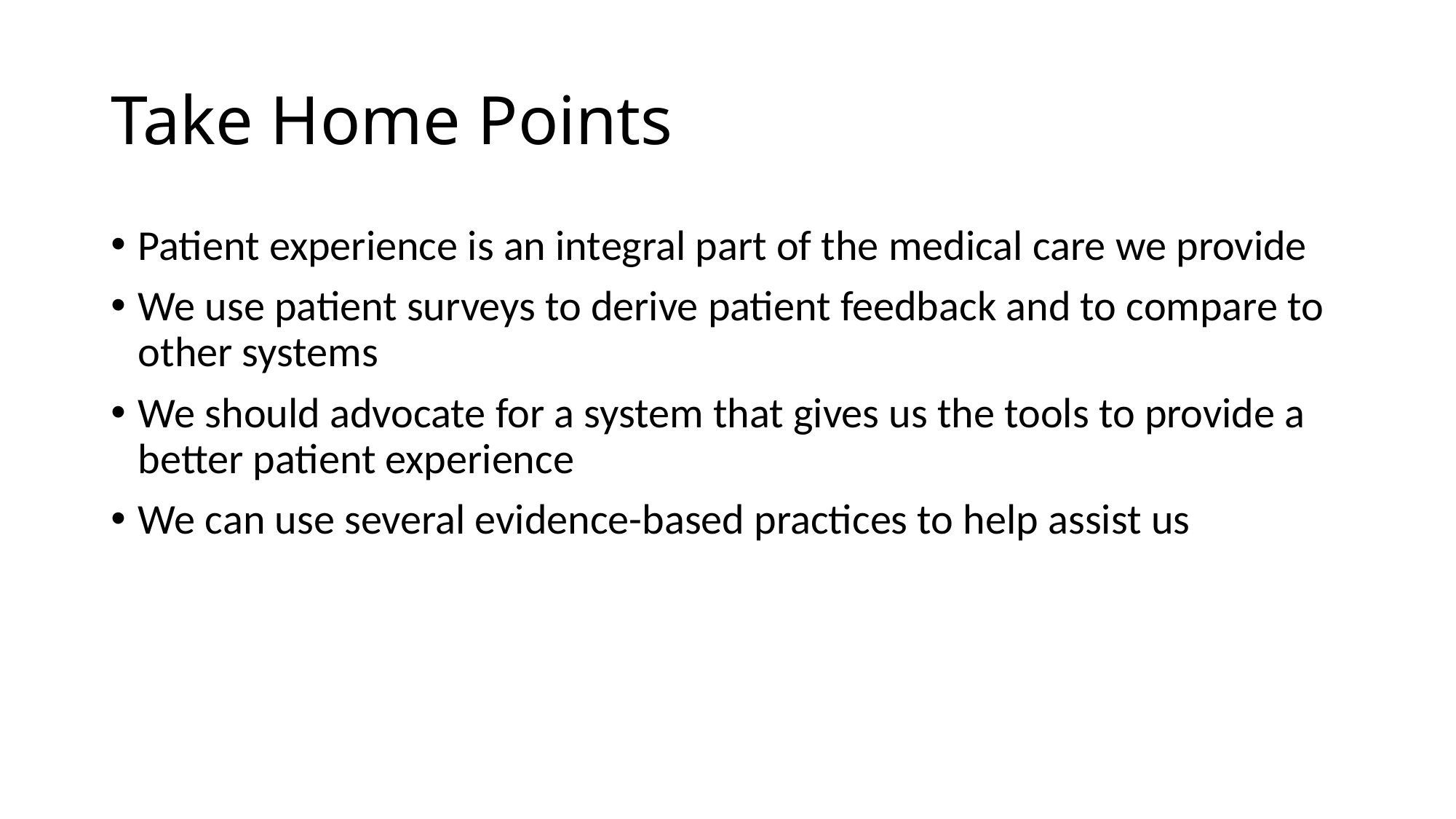

# Take Home Points
Patient experience is an integral part of the medical care we provide
We use patient surveys to derive patient feedback and to compare to other systems
We should advocate for a system that gives us the tools to provide a better patient experience
We can use several evidence-based practices to help assist us

## Slide 55
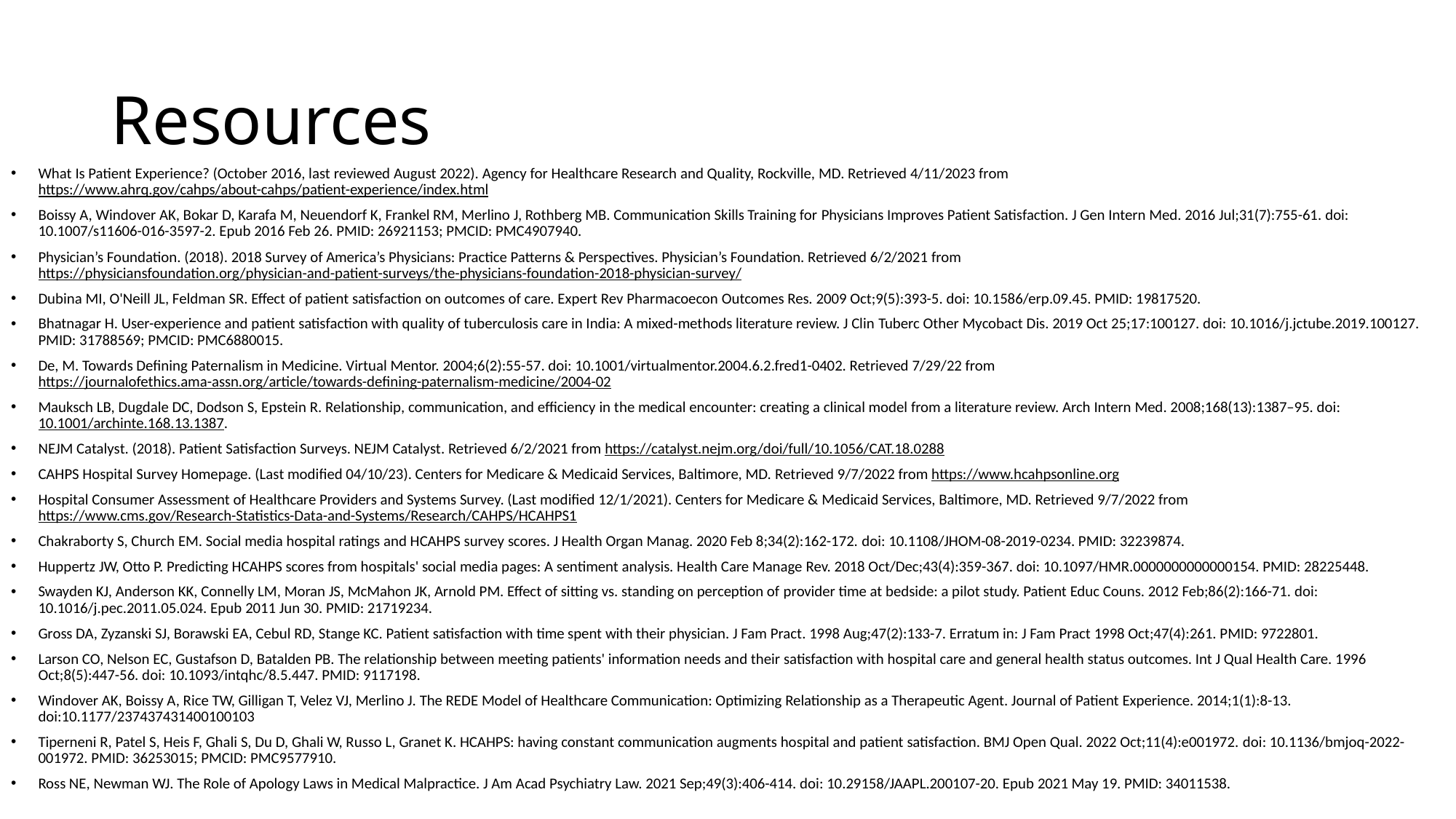

# Resources
What Is Patient Experience? (October 2016, last reviewed August 2022). Agency for Healthcare Research and Quality, Rockville, MD. Retrieved 4/11/2023 from https://www.ahrq.gov/cahps/about-cahps/patient-experience/index.html
Boissy A, Windover AK, Bokar D, Karafa M, Neuendorf K, Frankel RM, Merlino J, Rothberg MB. Communication Skills Training for Physicians Improves Patient Satisfaction. J Gen Intern Med. 2016 Jul;31(7):755-61. doi: 10.1007/s11606-016-3597-2. Epub 2016 Feb 26. PMID: 26921153; PMCID: PMC4907940.
Physician’s Foundation. (2018). 2018 Survey of America’s Physicians: Practice Patterns & Perspectives. Physician’s Foundation. Retrieved 6/2/2021 from https://physiciansfoundation.org/physician-and-patient-surveys/the-physicians-foundation-2018-physician-survey/
Dubina MI, O'Neill JL, Feldman SR. Effect of patient satisfaction on outcomes of care. Expert Rev Pharmacoecon Outcomes Res. 2009 Oct;9(5):393-5. doi: 10.1586/erp.09.45. PMID: 19817520.
Bhatnagar H. User-experience and patient satisfaction with quality of tuberculosis care in India: A mixed-methods literature review. J Clin Tuberc Other Mycobact Dis. 2019 Oct 25;17:100127. doi: 10.1016/j.jctube.2019.100127. PMID: 31788569; PMCID: PMC6880015.
De, M. Towards Defining Paternalism in Medicine. Virtual Mentor. 2004;6(2):55-57. doi: 10.1001/virtualmentor.2004.6.2.fred1-0402. Retrieved 7/29/22 from https://journalofethics.ama-assn.org/article/towards-defining-paternalism-medicine/2004-02
Mauksch LB, Dugdale DC, Dodson S, Epstein R. Relationship, communication, and efficiency in the medical encounter: creating a clinical model from a literature review. Arch Intern Med. 2008;168(13):1387–95. doi:10.1001/archinte.168.13.1387.
NEJM Catalyst. (2018). Patient Satisfaction Surveys. NEJM Catalyst. Retrieved 6/2/2021 from https://catalyst.nejm.org/doi/full/10.1056/CAT.18.0288
CAHPS Hospital Survey Homepage. (Last modified 04/10/23). Centers for Medicare & Medicaid Services, Baltimore, MD. Retrieved 9/7/2022 from https://www.hcahpsonline.org
Hospital Consumer Assessment of Healthcare Providers and Systems Survey. (Last modified 12/1/2021). Centers for Medicare & Medicaid Services, Baltimore, MD. Retrieved 9/7/2022 from https://www.cms.gov/Research-Statistics-Data-and-Systems/Research/CAHPS/HCAHPS1
Chakraborty S, Church EM. Social media hospital ratings and HCAHPS survey scores. J Health Organ Manag. 2020 Feb 8;34(2):162-172. doi: 10.1108/JHOM-08-2019-0234. PMID: 32239874.
Huppertz JW, Otto P. Predicting HCAHPS scores from hospitals' social media pages: A sentiment analysis. Health Care Manage Rev. 2018 Oct/Dec;43(4):359-367. doi: 10.1097/HMR.0000000000000154. PMID: 28225448.
Swayden KJ, Anderson KK, Connelly LM, Moran JS, McMahon JK, Arnold PM. Effect of sitting vs. standing on perception of provider time at bedside: a pilot study. Patient Educ Couns. 2012 Feb;86(2):166-71. doi: 10.1016/j.pec.2011.05.024. Epub 2011 Jun 30. PMID: 21719234.
Gross DA, Zyzanski SJ, Borawski EA, Cebul RD, Stange KC. Patient satisfaction with time spent with their physician. J Fam Pract. 1998 Aug;47(2):133-7. Erratum in: J Fam Pract 1998 Oct;47(4):261. PMID: 9722801.
Larson CO, Nelson EC, Gustafson D, Batalden PB. The relationship between meeting patients' information needs and their satisfaction with hospital care and general health status outcomes. Int J Qual Health Care. 1996 Oct;8(5):447-56. doi: 10.1093/intqhc/8.5.447. PMID: 9117198.
Windover AK, Boissy A, Rice TW, Gilligan T, Velez VJ, Merlino J. The REDE Model of Healthcare Communication: Optimizing Relationship as a Therapeutic Agent. Journal of Patient Experience. 2014;1(1):8-13. doi:10.1177/237437431400100103
Tiperneni R, Patel S, Heis F, Ghali S, Du D, Ghali W, Russo L, Granet K. HCAHPS: having constant communication augments hospital and patient satisfaction. BMJ Open Qual. 2022 Oct;11(4):e001972. doi: 10.1136/bmjoq-2022-001972. PMID: 36253015; PMCID: PMC9577910.
Ross NE, Newman WJ. The Role of Apology Laws in Medical Malpractice. J Am Acad Psychiatry Law. 2021 Sep;49(3):406-414. doi: 10.29158/JAAPL.200107-20. Epub 2021 May 19. PMID: 34011538.

## Slide 56
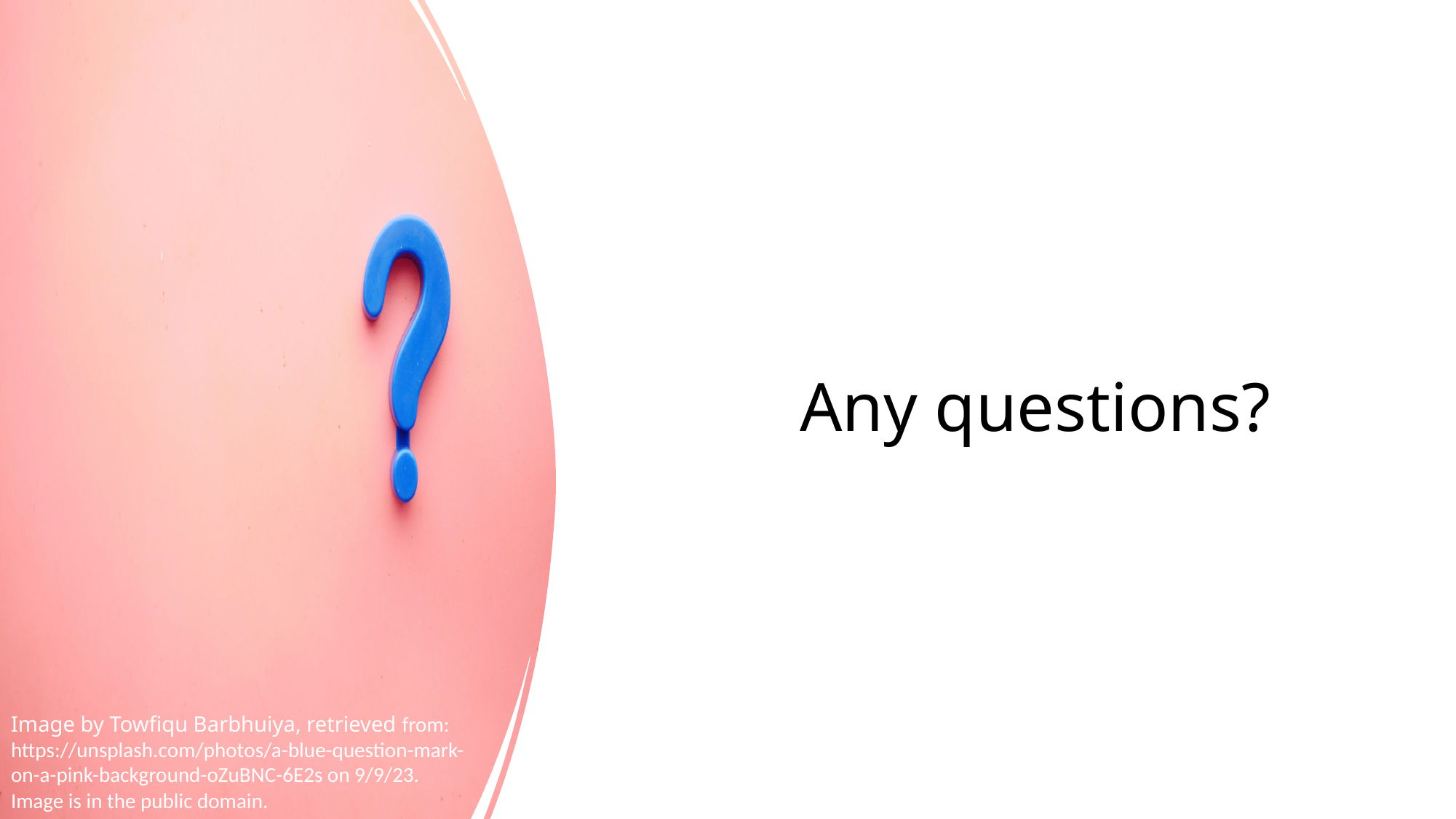

# Any questions?
Image by Towfiqu Barbhuiya, retrieved from: https://unsplash.com/photos/a-blue-question-mark-on-a-pink-background-oZuBNC-6E2s on 9/9/23. Image is in the public domain.

## Slide 57
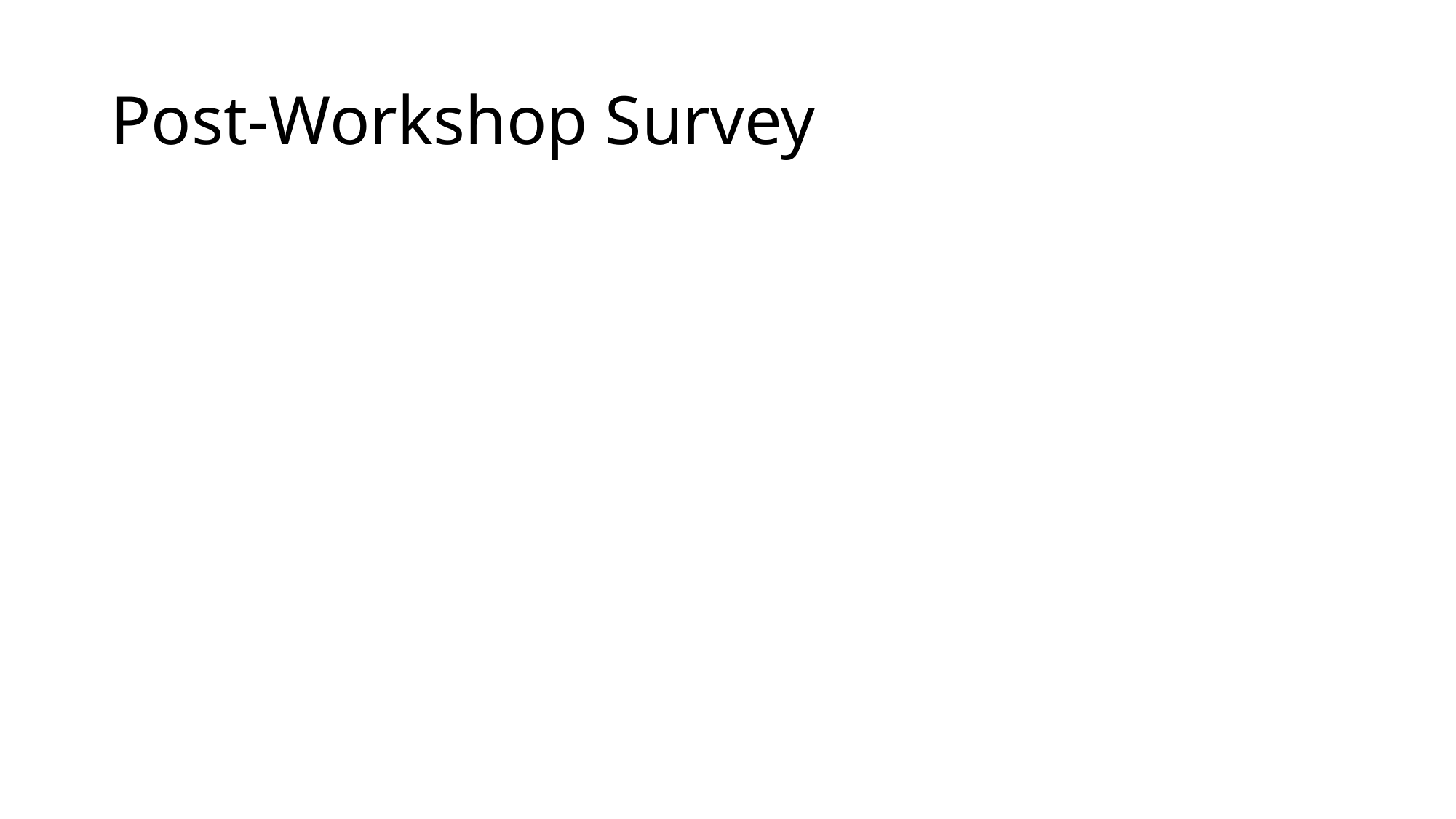

# Post-Workshop Survey
