# Supplementary material for: An Interactive, Case-Based Workshop on the Patient Experience for Internal Medicine Residents
Source: MedEdPORTAL. 2024 Oct 1;20:11442. doi: 10.15766/mep_2374-8265.11442 (PMC11442592; doi:10.15766/mep_2374-8265.11442)
Supplement: Supplementary file 1 — Preworkshop Survey.docxPostworkshop Survey.docxPatient Experience Workshop.pptxClinical Scenarios.docx [file mep_2374-8265.11442-s001.zip › D. Clinical Scenarios.docx]

**Appendix D. Clinical Scenarios for Discussion**

Please print out several copies of these scenarios (so that there are enough scenarios for distribution for each pair) and cut at dotted lines. Distribute one of the four clinical scenarios to each pair of residents for discussion. Ask them to discuss challenges faced and to suggest strategies in improving the patient experience. Allow 2 minutes for pair share followed by 3 minutes of whole-group discussion for each case (following the powerpoint slides). During whole group discussion first ask a volunteer to read the scenario out loud for everyone, then pairs who were given that scenario share suggestions on how to improve the patient experience in each case, followed by an open forum for others to chime in with their thoughts and input.

**Scenario 1 -** Your patient with sickle cell anemia returns to the hospital for another sickle cell pain crisis. After a few days being admitted with supportive care, their labs suggest improvement in hemolysis, so you start to wean IV opioids. On rounds, your patient asks for their usual 2mg IV hydromorphone since it was discontinued. Of note, the hematology team is also recommending that opioids should be weaned.

**Scenario 2 -** Your patient is admitted for chemo. A couple days into their hospital stay, the lab reported MRSA growing in their blood, and therefore their port was promptly removed with repeat blood cultures negative. The ID specialists found out that the blood sample was from a different patient. Now the patient must have port replaced and has lost time sitting in hospital bed. It is your job to inform them of the mistake.

**Scenario 3 -** Your previously “healthy” and active patient who used to work in construction has now been hospitalized for stroke with significant neuro deficits including hemiparesis for which they are currently bedbound. As your patient recovers, you sense your patient is now taking out frustration at providers and the medical team.

**Scenario 4 -** Your patient is admitted for Covid pneumonia with classic findings on CXR and CT. On rounds you see that the patient’s O2 requirements are not improving but remain stable on remdesivir. While you are still in the room, the patient’s family member is on the phone, and they express concern about the care you have provided, asking you why you haven’t started antibiotics yet (although you know very well that the evidence does not support).
