# Supplementary material for: An Interactive, Case-Based Workshop on the Patient Experience for Internal Medicine Residents
Source: MedEdPORTAL. 2024 Oct 1;20:11442. doi: 10.15766/mep_2374-8265.11442 (PMC11442592; doi:10.15766/mep_2374-8265.11442)
Supplement: Supplementary file 1 — Preworkshop Survey.docxPostworkshop Survey.docxPatient Experience Workshop.pptxClinical Scenarios.docx [file mep_2374-8265.11442-s001.zip › A. Preworkshop Survey.docx]

Presurvey to Patient Satisfaction Workshop

(Administer this presurvey on slide #2, recommended time to complete ~2 minutes)

Please complete the survey below. Thank you!

1. Date of Workshop:
2. Please enter your middle initial followed by the last four digits of your phone number (for statistical comparison purposes only):
3. Please select your level of training: PGY1 PGY2 PGY3 PGY4
4. Prior to today, how many times have you had a formal 0

lecture/didactic on this topic in your medical 1


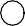

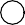

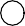

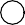


education/training? 2

3 or more

**Please choose your level of agreement with each of the following statements:**

1. I believe patient experience is an integral part of health care.
2. I know how hospitals track patient experience.
3. I know methods I can implement to improve the patient experience.

Strongly Disagree


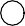

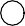

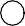


Somewhat Disagree


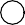

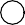

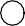


Neither Agree Nor Disagree


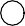

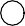

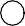


Somewhat Agree Strongly Agree


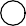

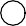

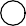

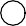

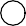

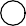


1.
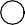

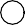

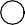

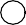

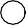
I understand how improving the patient experience can improve health outcomes.
2.
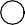

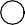

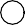

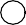

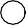
I believe patient experience training is important for my future as a practicing physician.

**A few days after a patient is discharged from the hospital, they receive the Hospital Consumer Assessment of Healthcare Providers and Systems (HCAHPS) survey via telephone call or by**

**mail. Please choose your level of agreement with each of the following statements:**

1. I know general questions that patients are asked on the HCAHPS survey.

Strongly

Disagree


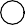


Somewhat

Disagree


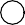


Neither Agree

Nor Disagree


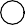


Somewhat Agree Strongly Agree


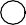

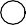


1.
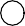

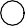

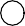

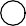

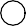
I know how hospitals use HCAHPS survey data to compare to one another.
2. Knowledge check: Please select the questions patients
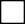
 During this hospital stay, how often did doctors are asked on the post-discharge HCAHPS survey listen carefully to you?

specifically related to their interaction with the
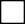
 During the hospital stay, how often did doctors physician. (SELECT ALL THAT APPLY) ask if you understood the treatment plan?


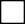
 During this hospital stay, how often did doctors explain things in a way you could understand?


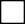
 During this hospital stay, how often did doctors clearly introduce themselves during visits?


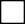
 During this hospital stay, how often did doctors treat you with courtesy and respect?

1. Knowledge check: Please select the methods you, as the
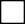
 During your visit turn on the lights, it is always physician, can do to improve patient satisfaction. better to have a patient interview with the lights (SELECT ALL THAT APPLY) on.


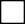
 Start with a warm greeting and introduce yourself to patient and family at bedside.


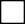
 Sit down to have a discussion because patients perceive that you spend more time with them with if you sit down.


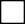
 Do not touch or examine your patient unless you have first put on gloves.


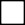
 Do not give your patient an expected length of stay in order to avoid disappointment.
