# Supplementary material for: An Interactive, Case-Based Workshop on the Patient Experience for Internal Medicine Residents
Source: MedEdPORTAL. 2024 Oct 1;20:11442. doi: 10.15766/mep_2374-8265.11442 (PMC11442592; doi:10.15766/mep_2374-8265.11442)
Supplement: Supplementary file 1 — Preworkshop Survey.docxPostworkshop Survey.docxPatient Experience Workshop.pptxClinical Scenarios.docx [file mep_2374-8265.11442-s001.zip › B. Postworkshop Survey.docx]

Post-Survey to Patient Experience Workshop

(Administer this post-survey on last slide, recommended time to complete ~5 minutes)

Please complete the survey below. Thank you!

Date of Workshop:

Please enter your middle initial followed by the last four digits of your phone number (for statistical comparison purposes only):

Please select your level of training: PGY1 PGY2 PGY3 PGY4


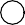

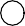

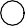

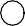


**Please choose your level of agreement with each of the following statements**

I believe patient experience is an integral part of health care.

I know how hospitals track patient experience.

I know methods I can implement to improve the patient experience.

Strongly

Disagree


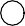

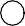

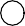


Somewhat

Disagree


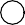

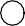

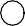


Neither Agree

Nor Disagree


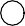

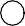

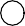


Somewhat Agree Strongly Agree


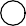

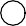

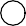

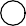

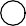

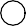


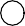

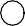

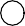

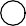

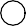
I understand how improving the patient experience can improve health outcomes.


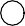

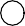

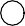

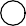

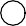
I believe patient experience training is important for my future as a practicing physician.

**A few days after a patient is discharged from the hospital, they receive the Hospital Consumer Assessment of Healthcare Providers and Systems (HCAHPS) survey via telephone call or by**

**mail. Please choose your level of agreement with each of the following statements:**

I know general questions that patients are asked on the HCAHPS survey.

Strongly

Disagree


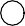


Somewhat

Disagree


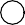


Neither Agree

Nor Disagree


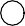


Somewhat Agree Strongly Agree


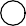

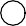


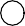

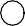

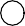

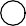

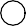
I know how hospitals use HCAHPS survey data to compare to one another.

Knowledge check: Please select the questions patients
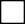
 During this hospital stay, how often did doctors are asked on the post-discharge HCAHPS survey listen carefully to you?

specifically related to their interaction with the
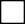
 During the hospital stay, how often did doctors physician. (SELECT ALL THAT APPLY) ask if you understood the treatment plan?


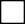
 During this hospital stay, how often did doctors explain things in a way you could understand?


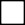
 During this hospital stay, how often did doctors clearly introduce themselves during visits?


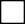
 During this hospital stay, how often did doctors treat you with courtesy and respect?

(the correct answers are the 1st, 3rd, and 5th options)

Knowledge check: Please select the methods you, as the
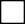
 During your visit turn on the lights, it is always physician, can do to improve patient experience. better to have a patient interview with the lights (SELECT ALL THAT APPLY) on.


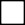
 Start with a warm greeting and introduce yourself to patient and family at bedside.


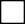
 Sit down to have a discussion because patients perceive that you spend more time with them with if you sit down.


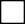
 Do not touch or examine your patient unless you have first put on gloves.


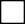
 Do not give your patient an expected length of stay in order to avoid disappointment.

(the correct answers are the 2nd and 3rd options)

**Rate this patient experience workshop and its components (1=not useful at all, 5=extremely**

**useful)**

1 2 3 4 5


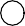

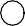

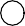

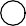

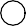
How would you rate the usefulness of the workshop in your clinical practice?


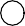

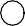

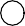

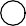

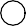
How would you rate the usefulness of the presentation/slide content?


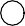

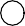

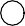

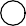

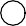
How would you rate the usefulness of the post discharge survey activity?


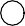

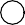

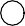

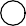

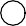
How would you rate the usefulness of the brainstorming through tough scenarios activity?


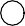

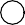

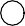

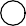

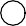
How would you rate the usefulness of the discussion with a hospital patient experience project manager?

If applicable, list a change you would make in your clinical practice as a result of things you learned in this workshop.

FREE TEXT FEEDBACK: What did you like about the workshop? What would you improve?
